# Uptake of Aβ by OATPs might be a new pathophysiological mechanism of Alzheimer disease

**DOI:** 10.1186/s12868-021-00658-9

**Published:** 2021-09-14

**Authors:** Jinhua Wen, Menghua Zhao, Wenxiong Sun, Xiaohua Cheng, Luyi Yu, Duanwen Cao, Pu Li

**Affiliations:** 1 Departmentof GCP/Psychosomatic Medicine, The First Affiliated Hospital of Nanchang University, Nanchang, China; 2 School of Pharmacy, Nanchang University, Nanchang, China

**Keywords:** OATPs, Alzheimer disease, Beta-amyloid

## Abstract

**Background:**

The accumulation of neurotoxic amyloid-beta (Aβ) in the brain is a characteristic of Alzheimer's disease (AD), at the same time, it is possible alterations of liver function could affect brain Aβ levels through changes in blood Aβ concentration. Over the last decade, a number of reports have shown that P-glycoprotein (encoded by *ABC1B1*) actively mediates the efflux transport of Aβ peptides. However, the mechanism by which Aβ peptides enter the cells is not clear. In the preliminary study, we found that the protein expression of organic anion transporting Polypeptide 1a4 (OATP1B1) in the liver tissue of mice with AD was significantly higher than that in the normal mice. In contrast, the protein expression of Oatp1a4 in the brain significantly decreased in mice with AD. OATP1B1, an important drug transporter might be related to the pathophysiology of AD.

**Results:**

In this study, we established an OATP1B1-GFP-HEK293T cell model to confirm the OATP1B1 mediated transport of Aβ_1-42_. Compared to the control group of GFP-HEK293Tcells, the uptake of Aβ_1-42_ protein in the OATP1B1-GFP-HEK293T group increased significantly with the increase in concentration of Aβ_1-42_, and also increased significantly with an increase in the duration of incubation. Similar results were observed in the flow cytometry experiment, and the uptake of Aβ_1-42_in HEK293T-OATP1B1 cells was almost twice that in the control group. These results indicate that OATPs may act as an important “carrier” for the transport of Aβ1-42 from the blood to the tissues, including liver and brain.

**Conclusions:**

This is a novel and interesting finding and OATP1B1 can be investigated as a new treatment target for AD.

**Supplementary Information:**

The online version contains supplementary material available at 10.1186/s12868-021-00658-9.

## Introduction

Alzheimer's disease (AD) is a neurodegenerative disease characterized by progressive loss of memory and acquired knowledge, until the complete loss of activities of daily life [1]. Alzheimer's disease endangers the health of the elderly following cardiovascular disease, cerebrovascular disease, and tumor [2]. According to the data released by the International Alzheimer's Association (ADI) in 2018, there are currently about 44.40 × 10^6^ patients with AD in the world, with an average of one new case detected every 3 s [3]. According to the statistics of the World Health Organization, AD will become the fourth highest cause of disease burden in China by 2020. This could lead to a heavy healthcare burden on the society and families, and develop into a serious social and health problem.

Although many scholars think that neurotoxicity caused by amyloid-beta (Aβ) is one of the main causes of AD, the exact pathogenesis of AD remains elusive. Currently, there is no effective method to treat or prevent the progression of AD. Therefore, it is imperative to understand the etiology, pathogenesis, and treatment target for AD as soon as possible, and to discover safe and effective treatment approaches in the field of AD research. It has been suggested that the imbalance between the clearance of Aβ and its accumulation in the peripheral and central systems is at the core of the development of AD [4]. However, the mechanism for correcting this imbalance is not clear. In recent years, drug transporters have played a very important role in the development of drugs, endogenous substances, and disease pathophysiology. Are these transporters related to the occurrence and development of AD? It has been shown that P-glycoprotein (P-gp, ABCB1) is an important barrier for the peripheral and central clearance of Aβ [5, 6], and P-gp can reduce the accumulation of Aβ in the tissues. However, a study showed that the ATPase activity measured in the vesicles of the plasma membrane of K562 cells overexpressing P-gp was not increased by the presence of Aβ_42_ [7], suggesting that Aβ_42_ is not a P-gp substrate [7]. Similarly, P-gp of pirarubicin was unaffected by the expression of Aβ_42_. Moreover, the overexpression of P-gp does not protect the cells against Aβ_42_ toxicity [7]. Considered together, these results indicate that Aβ_42_ is not transported by P-gp [7]. Therefore, although much evidence from human, animal, and in vitro studies has examined the contribution of P-gp in the clearance of Aβ, the role of P-gp in AD is still contentious [8].

All these previous studies focused on the efflux transporter of P-gp, but whether uptake transporters play the role in the pathophysiology of AD is unknown. As an important member of the solute delivery protein family, OATP has a wide range of substrates, including a variety of internal and external substances, especially the process of drugs in vivo, and its coding genes are collectively referred to as *SLCO* genes [9]. Among them, the specific expression of OATP1B1 (mouse hepatocyte expression homologous gene Oatp1a4) in the basolateral membrane of human hepatocytes has been widely studied, and its mediated substrate transport (drug) is very extensive [10]. Our research group tried to explore whether there is a relationship between OATP levels and AD. In a previous study, we analyzed the brain and liver tissue of mice with AD, and found that the protein expression of Oatp1a4 in the brain significantly decreased in the affected mice [10]. In contrast, the protein expression of Oatp1a4 in the liver tissue of mice with AD was significantly higher than that in the normal mice [10]. Results for the mRNA expression of Oatp1a4 showed that compared to that in the normal mice, it was significantly lower in the brain but significantly higher in the liver tissue in mice with AD [10]. However, the study could not confirm the relationship between Oatp1a4 and AD. A study showed that impaired hepatic Aβ degradation could be a factor contributing to increased brain Aβ accumulation and AD [11]. Therefore, it is significant to study the clearance or transport mechanism of Aβ in liver. OATP1B1 is expressed specifically in the liver, so we hope to explore whether it play a role in hepatic Aβ degradation. This is a very novel and interesting study that might open a new door for research on the pathophysiology of AD.

## Materials and methods

### Materials and main instruments

0.45 μm PVDF membrane, Millipore Inc. (Massachusetts, USA); Skimmed milk powder, Yili Industrial Group Co., Ltd. (Hohhot, China). BEYOCOLOR color pre-dyed protein molecular weight standard: Fermentas Inc (Canada); ECL plus luminescent kit, SDS-PAGE protein sample buffer (5 ×), Western and IP cell lysate, PMSF and BCA protein concentration determination kit were purchased from Beyotime Institute of Biotechnology (Shanghai, China); Tris HCl/SDS (1.5 mm, pH 8.8) and Tris HCl/SDS (0.5 mm, pH 6.8) were purchased from Shanghai Biotechnology Co., Ltd.; 30% acrylamide/bis solution, glycine were purchased from Bio-RAD (California, USA); Tris alkali, SDS and ammonium persulfate were purchased from BiosharpInc.; Aβ_1-42_ monomer, BiosharpInc (bs0076R, Hefei, China); Anti-OATP1B1 antibody (abcam, UK, ab224610); Anti- Aβ_1-42_ antibody (Bioss, Beijing, bs0076R); Aβ_1-42_ (Jier, Shanghai, JR10010085); Goat anti mouse IgG, Allied biology company (GAM007, Shenzheng, China); Goat anti rabbit IgG, Allied biology company (GAR007, Shenzheng, China); β-actin, Allied biology company (ab008, Beijing, China); HEK293T cells (single clones) and OATP1B1 virus, Hangzhou HibioTechnology Co., Ltd. (Hangzhou, China); fetal bovine serum, GibcoInc. (California, USA); Rapid total RNA Extraction Kit, Shanghai Jierui Bioengineering Co., Ltd. (Shanghai, China); The reverse transcription kit (HiScript II Q RT SuperMix for qPCR) and Quantitative PCR kit (ChamQTM SYBR Color Qpcr Master Mix), Vazyme Biotech Co., Ltd. (Nanjing, China).

Flow cytometer: Becton, Dickinson and Company (New Jersey, USA); Cell incubator, Thermo Fisher Scientific (Massachusetts, USA); Inverted microscope (Olympus company, Japan). Desktop low-speed centrifuge, Shanghai medical equipment (Group) Co., Ltd. (Shanghai, China); Mini-Proten Tetra System, Bio-RAD, (California, USA); ChemiDoc XRS + System, Bio-RAD (California, USA); Low light spectrophotometer, Beijing Meilin Hengtong Technology Co., Ltd. (Beijing, China).

### Methods

HEK293T cells and OATP1B1 virus were obtained from Hangzhou HibioTechnology Co., Ltd. (Hangzhou, China). The OATP1B1-GFP-HEK293T and GFP-HEK293T cell models were established as previously described [9]. The OATP1B1 sequence was synthesized into a pEGFP-N1 vehicle [9]. Then, the vehicle was transfected into DH5α competent cells to generate more pEGFP-N1-OATP1B1 plasmids [9]. Finally, the pEGFP-N1-OATP1B1 plasmid was transfected into HEK293 cells [9]. Then, qPCR and western blot testing were used to detect the expression of OATP1B1 in the cells The OATP1B1-GFP-HEK293T and GFP-HEK293T cell models were established as previously described [9]. Western Blotting was used to detect the expression of OATP1B1. The process of the Western blot analysis includes protein extraction, gel making, sample loading, sodium dodecyl sulfate–polyacrylamide gel electrophoresis, membrane transfer, immune response (Primary antibody was Anti-OATP1B1 1: 1000; secondary antibodies were Goat anti-Mouse IgG and Goat anti-Rabbit IgG 1: 5000), chemiluminescence, and gel image analysis [10]. The complete RNA was extracted using a Trizol centrifugal column, and reverse transcription was carried out to obtain the cDNA, then PCR amplification was done. Primers for OATP1B1 were OATP1B1-F: AACTCCTACTGATTCTCGATGGG; OATP1B1-R: GTTTCCAGCACATGCAAAGAC; actin-F: TGACGTGGACATCCGCAAAG; actin-R: CTGGAAGGTGGACAGCGAGG. Finally, a PCR system was established, and the mRNA levels of OATP1B1-GFP-HEK293T cells and GFP-HEK293T cells were analyzed using a real-time quantitative PCR detection system [10]. OATP1B1-GFP-HEK293T cells and the control cell line GFP-HEK293T were used to explore the uptake features of Aβ_1-42_. All HEK293 stable cell lines were maintained in dulbecco's modified eagle medium (DMEM) containing 10% fetal bovine serum, 1% antibiotic, and antimycotic solution, and 600 µg ml^−1^ geneticin [9]. The cell lines were cultured in a humidified atmosphere (95% O_2_, 5% CO_2_) at 37 °C [9]. Aβ_1-42_ peptide powder was dissolved in phosphate-buffered saline (DMSO) to a final concentration of 1 mM. Then, the peptides were snapping frozen in liquid nitrogen. The aliquoted peptide were incubated for 1 week at 37 °C before use, then were dissolved in culture medium to working concentration for treatment. Cell toxicity was performed to select reasonable concentrations for the uptake experiments. CCK8 assay was used to detect cell viability. Next, a series of concentrations of Aβ_1-42_ (0, 0.4, 1.0, and 2.5 µM) were added to the OATP1B1-GFP-HEK293T and GFP-HEK293 cells and incubated for approximately 24, 48, and 72 h, respectively. Then, the cells were washed with ice-cold phosphate buffer saline (PBS) 3 × and lysed with cell lysis buffer. Cell lysis buffer was collected and centrifuged at 1.4 × 10^4^ rpm for 20 min. The supernatants were used to analyze the Aβ_1-42_ by Western Blotting (Anti- Aβ_1-42_ antibody, 1: 1000). At the same time, the cells were collected to detect the uptake of Aβ_1-42_ in both OATP1B1-GFP-HEK293T and GFP-HEK293 cells by flow cytometry. In the flow cytometry process, GFP fluorescence of cells was detected by transverse FL1A channel, and Aβ_1-42_ Label fluorescence was detected by longitudinal FL4A channel. The double positive cells in the upper right quadrant were HEK293T cells successfully carrying GFP and ingesting Aβ_1-42_. By calculating the proportion of double positive cells in the upper right quadrant, the proportion of cells in each group that ingested Aβ_1-42_ was obtained [12]. By calculating the fluorescence intensity of Aβ_1-42_ in the double positive cells in the upper right quadrant, the relative amount of Aβ_1-42_ ingested in each group of cells was obtained [12]. All these methods been adhered to biosecurity and institutional safety procedures of the First Affiliated Hospital of Nanchang University see Additional files 1–13.

### Statistical analysis

All statistical analyses were performed using Student’s t-test and one-way ANOVA, with SPASS 13.0. Data are presented as mean ± standard deviation from at least three separate experiments (n = 3). * and ** indicate p < 0.05 and p < 0.01, respectively. P value less than 0.05 was considered statistically significant. Western Blot bands were calculated using Image J software (Java 1.6.0_20, NIH, USA).

## Results

**Fig. 1:**
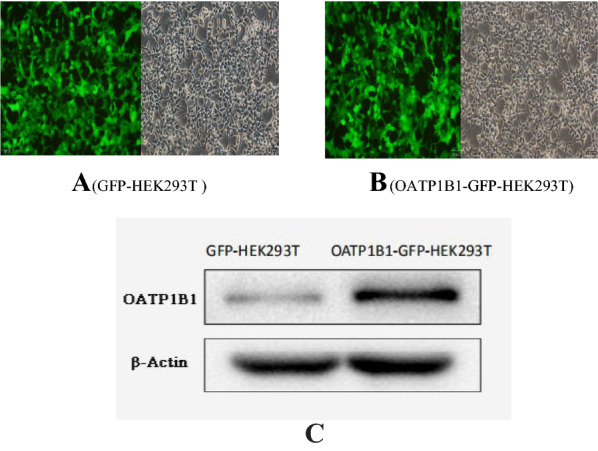
Successfully established OATP1B1-GFP-HEK293T cell model (**A**) in which OATP1B1 was highly expressed. Compared to the control GFP-HEK293T cells (**B**), OATP1B1 expression increased about 214% in the OATP1B1-GFP-HEK293T cells. Both, GFP-HEK293T cells and OATP1B1-GFP-HEK293T cells were cultured in regular DMEM with 10% FBS. OATP1B1expression in the GFP-HEK293 cells and HEK293T-OATP1B1 cells were detected by western blot (**C**), and showed significant difference between the two cell models. β-actin served as an internal control

**Fig. 2:**
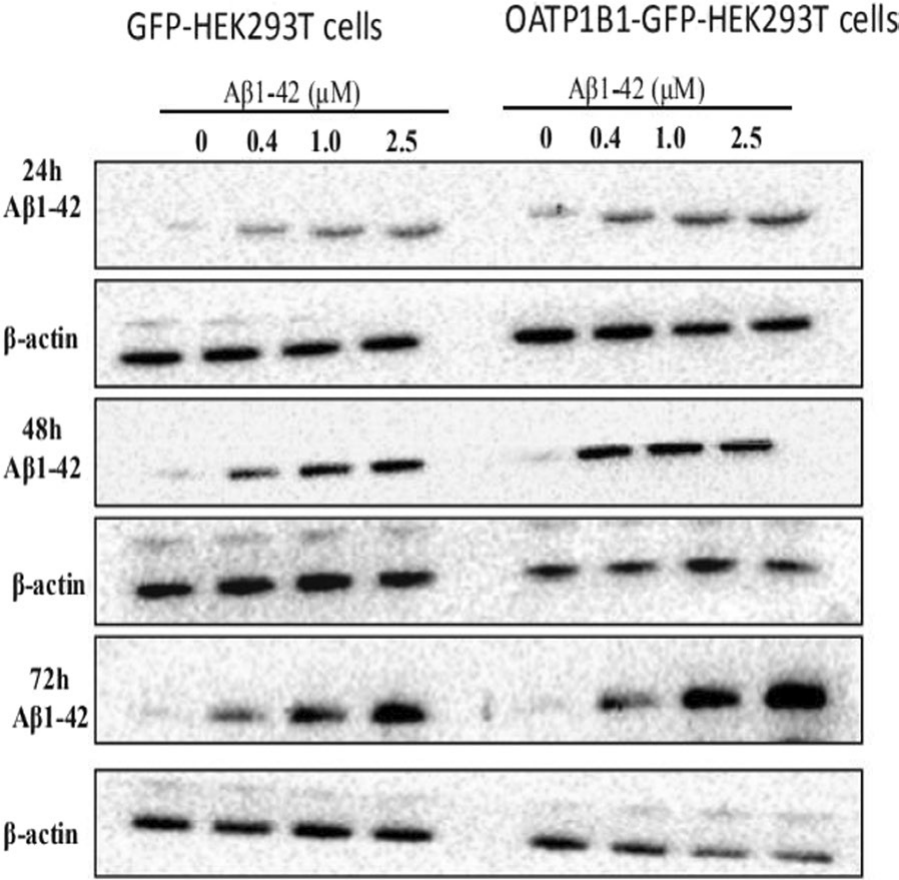
GFP-HEK293T cells and OATP1B1-GFP-HEK293T cells were cultured in 6-well plates at 2.5 × 10^5^ cells/well and after adherence, were treated withAβ_1-42_ (0, 0.4, 1, 2.5 μM) for 24, 48, and 72 h. Following treatment, Aβ_1-42_ was determined in the cell lysates by western blotting (WB). Compared to the GFP-HEK293T cells group, the uptake of Aβ_1-42_ protein in the OATP1B1-GFP-HEK293T group increased with the increase in Aβ_1-42_ concentration. The increase was significant with the increase in incubation time. Aβ_1-42_ bands to oligomer was about 64KD. β-actin served as an internal control

**Fig. 3:**
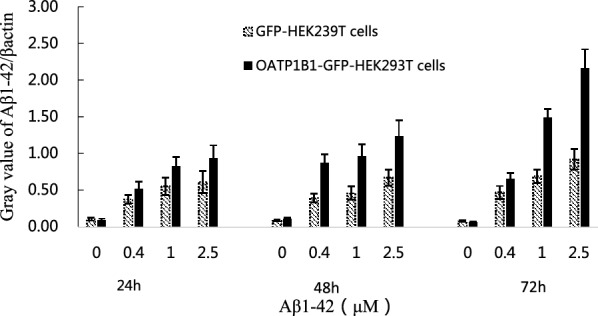
Western Blot bands were calculated and Aβ_1-42_/β-actin ratio value represented the indirect uptake of Aβ_1-42_ in the cells. The results showed that Aβ_1-42_ uptake in GFP-HEK293T cells and OATP1B1-GFP-HEK293T cells was 0.11 ± 0.02 vs 0.10 ± 0.02, 0.38 ± 0.06 vs 0.52 ± 0.10, 0.56 ± 0.12 vs 0.83 ± 0.13, 0.62 ± 0.15 vs 0.93 ± 0.18 when the cells were treated with Aβ_1-42_ (0, 0.4, 1, 2.5 μM) for 24 h, while they were 0.09 ± 0.01 vs 0.12 ± 0.01, 0.40 ± 0.06 vs 0.87 ± 0.12, 0.46 ± 0.09 vs 0.97 ± 0.16, 0.68 ± 0.11 vs 1.24 ± 0.21 for 48 h, and 0.08 ± 0.01 vs 0.07 ± 0.01,0.47 ± 0.09 vs 0.66 ± 0.08,0.69 ± 0.09 vs 1.49 ± 0.11,0.92 ± 0.14 vs 2.16 ± 0.26 for 72 h

We successfully created an OATP1B1-GFP-HEK293T cell model and compared it to the control. OATP1B1 increased by approximately 214% in the OATP1B1-GFP-HEK293T cells (Fig. 1). The qPCR results also showed that the mRNA expression of OATP1B1 in the OATP1B1-GFP-HEK293T cells was significantly higher than that in the GFP-HEK293T cells (1.03 ± 0.22 vs 0.00 ± 0.00). After treatment with Aβ_1-42_ (0, 0.4, 1, 2.5 μM) for 24, 48, and 72 h, by using Western Blotting, the uptake of Aβ_1-42_ inOATP1B1-GFP-HEK293T cells significantly increased with increasing Aβ_1-42_ concentration and the duration of incubation. Similar results were also seen in the HEK293T cells; however, OATP1B1-GFP-HEK293T cells mediated uptake of Aβ_1-42_ was higher than that of GFP-HEK293 cells, especially when the incubation time was 72 h (Fig. 2). From the gray value of the Western Blot, the results showed that Aβ_1-42_ uptake in GFP-HEK293T cells and OATP1B1-GFP-HEK293T cells was 0.11 vs 0.10, 0.38 vs 0.52, 0.56 vs. 0.83, 0.62 vs 0.93 when the cells were treated with Aβ_1-42_ (0, 0.4, 1, and 2.5 μM) for 24 h, while they were 0.09 vs 0.12, 0.40 vs 0.87, 0.46 vs 0.97, 0.68 vs 1.24 for 48 h, and 0.08 vs 0.07, 0.47 vs 0.66, 0.69 vs 1.49, 0.92 vs. 2.16 for 72 h ( Fig. 3).

**Fig. 4:**
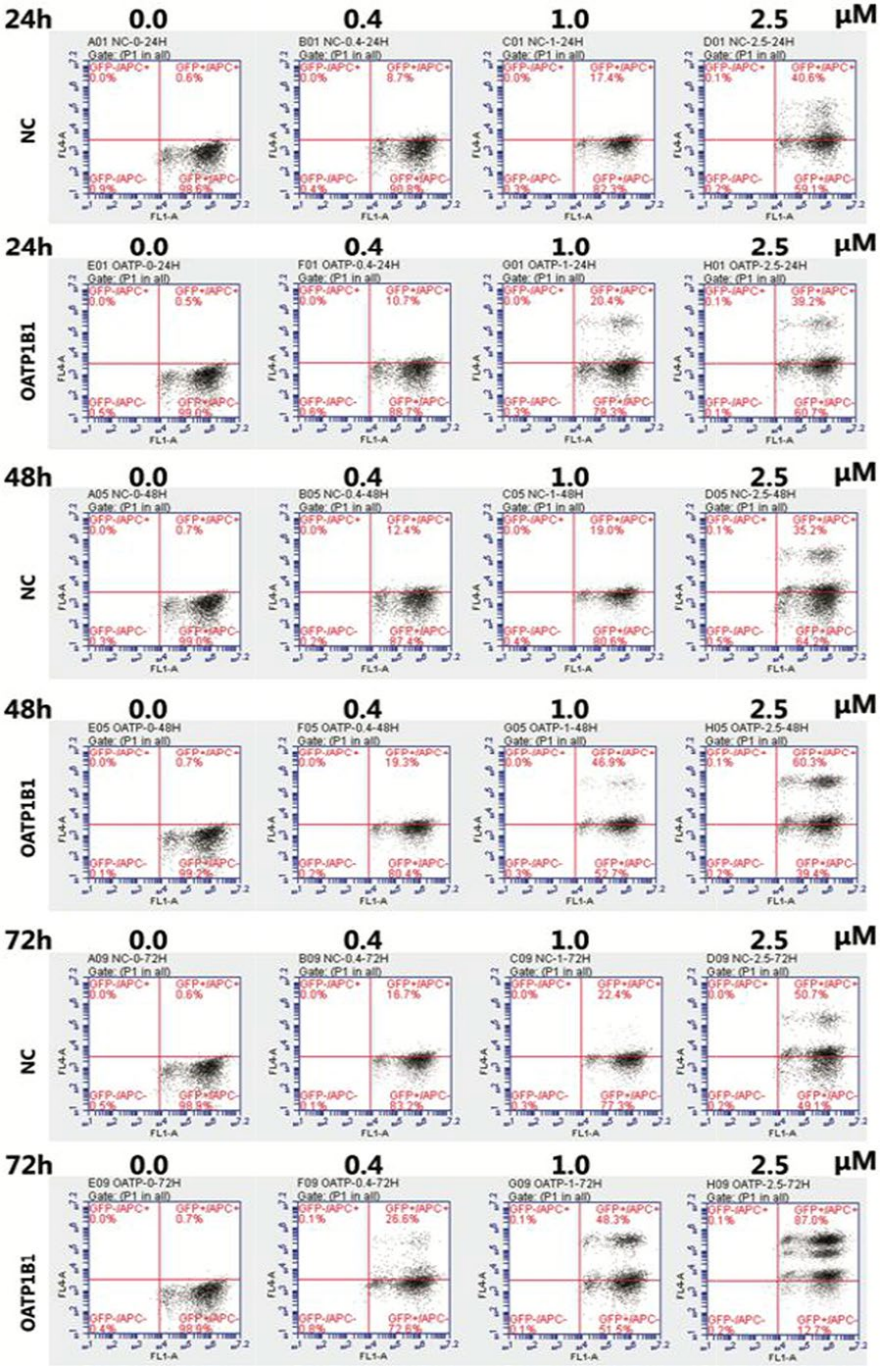
In the flow cytometry diagram, GFP fluorescence of cells was detected by transverse FL1A channel, and Aβ _1–42_ Label fluorescence was detected by longitudinal FL4A channel. The double positive cells in the upper right quadrant were HEK293T cells successfully carrying GFP and ingesting Aβ_1-42_. By calculating the proportion of double positive cells in the upper right quadrant, the proportion of cells in each group that ingested Aβ_1-42_ was obtained (Fig. 4 and Table 1). By calculating the fluorescence intensity of Aβ_1-42_ in the double positive cells in the upper right quadrant, the relative amount of A β_1-42_ ingested in each group of cells was obtained (shown in Fig. 5 and Table 2). NC:GFP-HEK293T cells; OATP1B1: OATP1B1-GFP-HEK293T cells

**Fig. 5:**
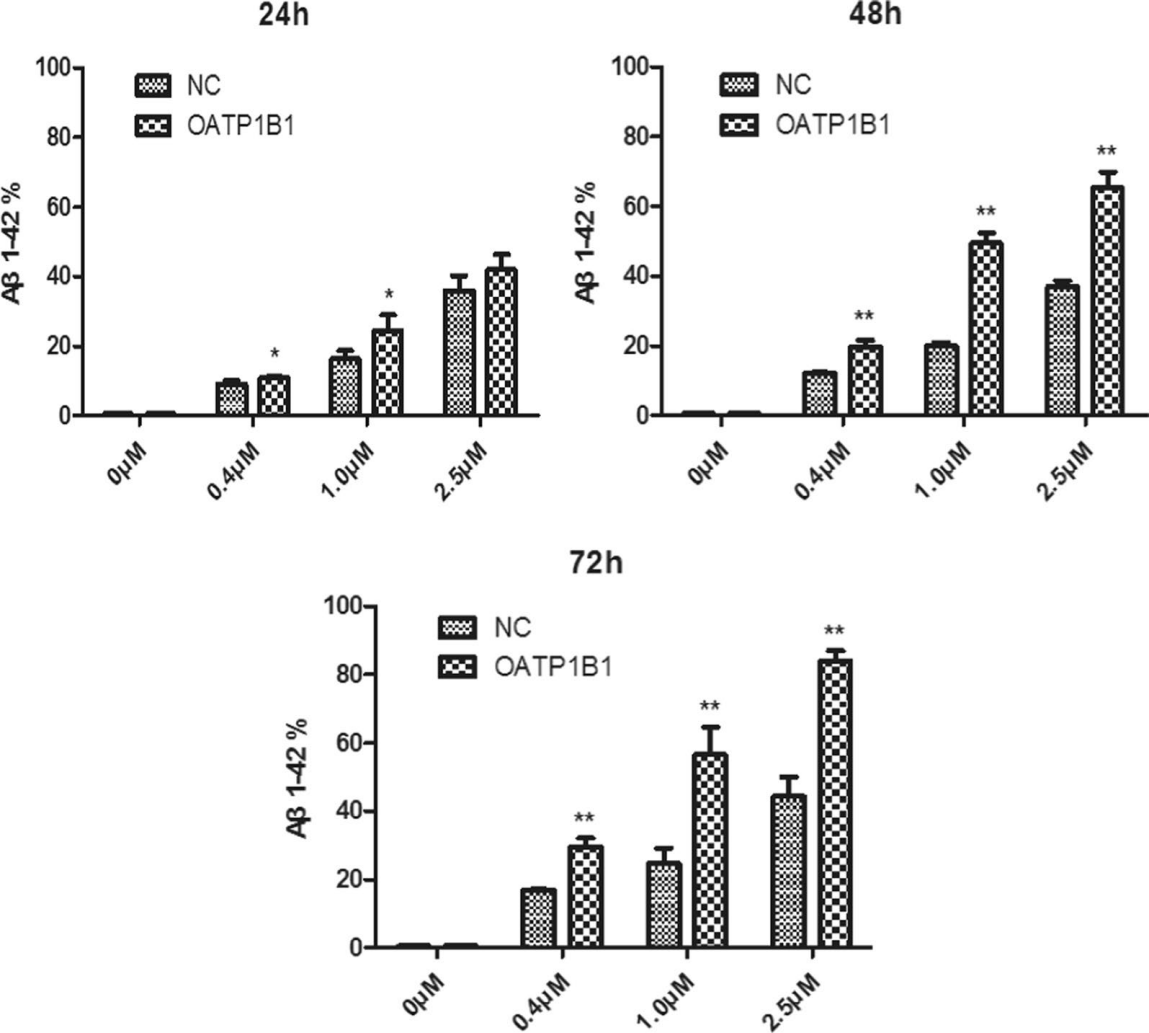
Flow cytometry showing the cellular uptake of Aβ _1–42_ in GFP-HEK293T cells and OATP1B1-GFP-HEK293T cells following incubation with 0.4, 1, 2.5 μM Aβ (1–42) for 24, 48, 72 h, respectively. Uptake of Aβ _1–42_ increased both in GFP-HEK293T cells and OATP1B1-GFP-HEK293T, and compared to GFP-HEK293T cells, the uptake of Aβ_1-42_ in OATP1B1-GFP-HEK293Tcells increased significantly with the increase in duration of incubation. Especially, when the incubation time was 72 h, the uptake of Aβ_1-42_ in OATP1B1-HEK293T was almost twice as much as that in the HEK293T group. *indicates P < 0.05; **indicates P < 0.01. NC: GFP-HEK293T cells; OATP1B1:OATP1B1-GFP-HEK293T cells. n = 3

**Fig. 6:**
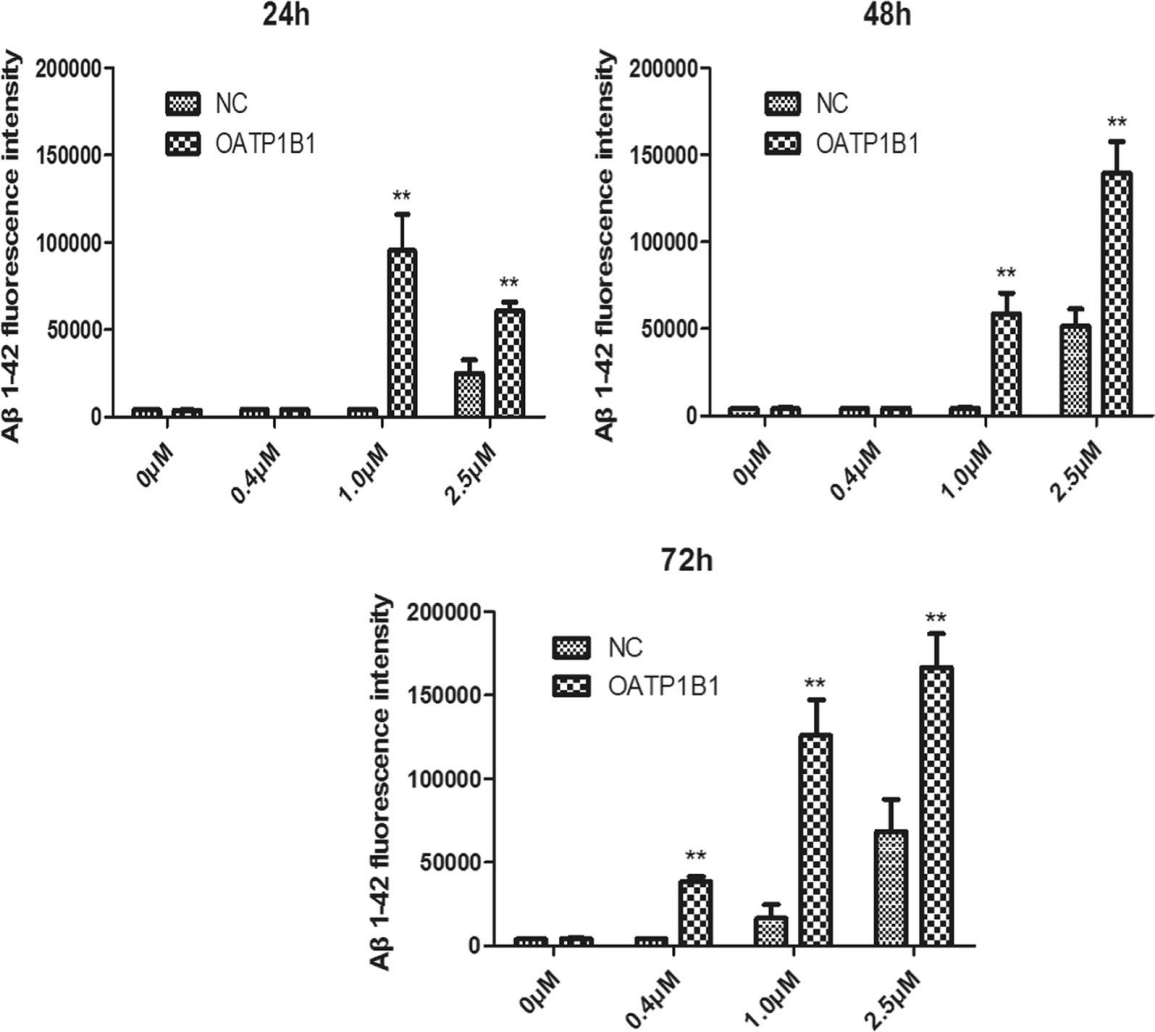
Fluorescence intensity of Aβ_1-42_ in the GFP-HEK293T cells and OATP1B1-GFP-HEK293T cells. The fluorescence intensity of Aβ _1–42_ in OATP1B1-GFP-HEK293T cells was apparently higher than that in the GFP-HEK293T cells when the cells were treated with 1.0 and 2.5 μM Aβ_1-42_ during the incubation time of 24–72 h. Especially, as the duration of incubation increased to 72 h, the fluorescence intensity of Aβ _1–42_ in OATP1B1-GFP-HEK293T cells was significantly higher than that in the GFP-HEK293T cells in the concentration range of 0.4–2.5 μM. *indicates P < 0.05; **indicates P < 0.01. NC:GFP-HEK293T cells; OATP1B1:OATP1B1-GFP-HEK293T cells. n = 3

From the results of flow cytometry (Fig. 4), we observed that the uptake of Aβ_1-42_ increased both GFP-HEK293T and OATP1B1-GFP-HEK293T cells, and compared to the HEK293T cells, the uptake of Aβ_1-42_ in OATP1B1-GFP-HEK293T cells increased significantly with the increase in the duration of incubation (Fig. 5 and Table 1). By calculating the fluorescence intensity of Aβ_1-42_ in the GFP-HEK293T and OATP1B1-GFP-HEK293T cells, it is apparent that the intensity of Aβ_1-42_ in OATP1B1-GFP-HEK293T cells was higher than that in GFP-HEK293T cells. The results are shown in Fig. 6 and Table 2. Results of both Western Blotting and flow cytometry confirmed that Aβ_1-42_ was the substrate of OATP1B1. OATP1B1 is involved in the transport of Aβ_1-42_ in tissues.

**Table 1:** The cellular uptake of Aβ_1-42_ in GFP-HEK293T cells and OATP1B1-GFP-HEK293T cells following incubation with 0.4, 1, 2.5 μM Aβ_1-42_ for 24, 48, 72 h, respectively (n = 3)

	Aβ_1-42_ 0 μM		Aβ_1-42_ 0.4 μM		Aβ_1-42_1 μM		Aβ_1-42_ 2.5 μM	
	NC	OATP1B1	NC	OATP1B1	NC	OATP1B1	NC	OATP1B1
24 h	0.52 ± 0.05	0.47 ± 0.12	9.16 ± 0.93	10.88 ± 0.31*	16.28 ± 2.42	24.50 ± 4.48*	36.01 ± 4.25	42.00 ± 4.27
48 h	0.62 ± 0.11	0.67 ± 0.15	12.18 ± 0.25	19.87 ± 1.70**	20.07 ± 0.98	49.42 ± 3.02**	37.04 ± 1.77	65.38 ± 4.52**
72 h	0.52 ± 0.07	0.63 ± 0.10	16.92 ± 0.20	29.53 ± 2.58**	24.61 ± 4.57	56.60 ± 8.03**	44.39 ± 5.76	84.13 ± 3.03**

*NC* GFP-HEK293T cells; *OATP1B1* OATP1B1-GFP-HEK293T cells

*P < 0.05; **P < 0.01

**Table 2:** Fluorescence intensity of Aβ_1-42_ in the GFP-HEK293Tand OATP1B1-GFP-HEK293T cells when the cells were treated with different concentration of Aβ_1-42_ during the different incubation time (n = 3)

	Aβ1-42 0 μM		Aβ1-42 0.4 μM		Aβ1-42 1 μM		Aβ1-42 2.5 μM	
	NC	OATP1B1	NC	OATP1B1	NC	OATP1B1	NC	OATP1B1
24 h	4162 ± 336	3943 ± 167	4386 ± 63	4316 ± 52	4316 ± 107	95484 ± 20582	25148 ± 7713	60935 ± 4789
48 h	3980 ± 145	4100 ± 566	4333 ± 19	4356 ± 74	4440 ± 323	58629 ± 12127	51704 ± 9795	139172 ± 18360
72 h	4115 ± 225	4233 ± 562	4335 ± 52	38803 ± 2597	16363 ± 8540	126123 ± 21186	68502 ± 19324	166943 ± 20123

*NC* GFP-HEK293T cells; *OATP1B1* OATP1B1-GFP-HEK293T cells

## Discussion

One study demonstrated that endocytosis is the major, if not the only pathway for the entry of Aβ_1-40_ and Aβ_1-42_ into the SH-SY5Y cells [13]. This disputes some previous beliefs about passive diffusion or membrane-penetration modes of entry, which would allow Aβ direct access to the cytoplasm [14]. Simultaneously, the clearance of cerebral Aβ is a complex process mediated by various systems and cell types, including vascular transport across the blood–brain barrier, glymphatic drainage, and engulfment and degradation by the resident microglia and infiltrating innate immune cells [5]. Therefore, the process for the uptake and efflux of Aβ is complex and chaotic. Our study showed that Aβ_1-42_ was the substrate of OATP1B1, which is a novel and interesting finding. A study showed that impaired hepatic Aβ degradation could be a factor contributing to increased brain Aβ accumulation and AD [11]. Rodent studies have suggested that the liver has a major role in Aβ degradation [15, 16]. It is possible alterations of liver function could affect brain Aβ levels through changes in blood Aβ concentration [11]. As an uptake transporter that might play an important role in the cellular uptake of Aβ_1-42_, OATP1B1will become a new target for the treatment of AD. We found that the protein expression of Oatp1a4 (OATP1B1) in the liver tissue of mice with AD was significantly higher than that in the normal mice. In contrast, the protein expression of Oatp 1a4 in the brain was significantly lower in mice with AD [10]. OATP1B1 might have a “self-defense system role” that could decrease the uptake quantity of Aβ_1-42_ in the blood and decrease the uptake of Aβ_1-42_ in brain. At the same time, it is necessary to confirm whether other members of the OATPs families act as a “carrier” of Aβ_1-42_. Despite decades of research, the pathophysiology of AD remains elusive. Understanding the normal versus impaired processing and clearance mechanisms affecting Aβ peptides will assist in the development of more effective therapeutic agents to combat this progressive neurodegenerative condition that continues to devastate millions of patients globally [8]. We hope that this study will be helpful in the research for the pathophysiology of AD. The active uptake pathway for the entry of Aβ_1-42_ into the nerve cells by the OATPs is likely to become a new and novel mechanism of the pathophysiology of AD. In future, we will explore the role of OATP1A2 in uptake of Aβ_1-42_ in blood brain barrier and study the protein expression of Aβ_1-42_ in OATPs knock out AD mouse model.

## Supplementary Information

**Additional file 1: **Raw western blot of actin in Figure 1.

**Additional file 2: **Raw western blot of OATP1B1 in Figure 1.

**Additional file 3: **Raw western blot of Aβ1-42 after incubation for 24h in Figure 2.

**Additional file 4: **Raw western blot of Aβ1-42 after incubation for 48h in Figure 2.

**Additional file 5: **Raw western blot of Aβ1-42 after incubation for 72h in Figure 2.

**Additional file 6: **Raw western blot of βactin after incubation for 24h in Figure 2.

**Additional file 7: **Raw western blot of βactin after incubation for 48h in Figure 2.

**Additional file 8: **Raw western blot of βactin after incubation for 72h in Figure 2.

**Additional file 9: **Raw Figures for flow cytometry after incubation for 24h in Figure 4

**Additional file 10: **Raw Figures for flow cytometry after incubation for 48h in Figure 4.

**Additional file 11: **Raw Figures for flow cytometry after incubation for 72h in Figure 4.

**Additional file 12: **Raw data for Figure 5 and Figure 6.

**Additional file 13: ** multiple exposures-Figure2 -Aβ1-42-24h Administrator 2020-05-22 13 h 48 min_Exposure_50.0sec. multiple exposures-Figure2 -Aβ1-42-24h Administrator 2020-05-22 13 h 48 min_Exposure_75.9sec. multiple exposures-Figure2 -Aβ1-42-24h Administrator 2020-05-22 13 h 48 min_Exposure_300.0sec. multiple exposures-Figure2 -Aβ1-42-48h Administrator 2020-05-22 13 h 33 min_Exposure_50.0sec. multiple exposures-Figure2 -Aβ1-42-48h Administrator 2020-05-22 13 h 33 min_Exposure_205.2sec. multiple exposures-Figure2 -Aβ1-42-48h Administrator 2020-05-22 13 h 33 min_Exposure_291.4sec. multiple exposures-Figure2 -Aβ1-42-72h Administrator 2020-05-22 11 h 17 min_Exposure_58.6sec. multiple exposures-Figure2 -Aβ1-42-72h Administrator 2020-05-22 11 h 17 min_Exposure_231.0sec. multiple exposures-Figure2 -Aβ1-42-72h Administrator 2020-05-22 11 h 17 min_Exposure_300.0sec. multiple exposures-Figure2 -βactin-24h Administrator 2020-05-22 11 h 36 min_Exposure_50.0sec. multiple exposures-Figure2 -βactin-24h Administrator 2020-05-22 11 h 36 min_Exposure_239.6sec. multiple exposures-Figure2 -βactin-24h Administrator 2020-05-22 11 h 36 min_Exposure_300.0sec. multiple exposures-Figure2 -βactin-48h Administrator 2020-05-22 10 h 58 min_Exposure_50.0sec. multiple exposures-Figure2 -βactin-48h Administrator 2020-05-22 10 h 58 min_Exposure_282.7sec. multiple exposures-Figure2 -βactin-48h Administrator 2020-05-22 11 h 58 min_Exposure_300.0sec. multiple exposures-Figure2 -βactin-72h Administrator 2020-05-22 11 h 50 min_Exposure_50.0sec. multiple exposures-Figure2 -βactin-72h Administrator 2020-05-22 11 h 50 min_Exposure_205.2sec. multiple exposures-Figure2 -βactin-72h Administrator 2020-05-22 11 h 50 min_Exposure_300.0sec.

## Data Availability

The datasets generated and/or analysed during the current study are available in the [professor] repository, https://osf.io/tnz57/?view_only=f9e6fa6ec3fe44549069f472be380197.

## References

[1] Dufort-Gervais J, Mongrain V, Brouillette J (2019). Bidirectional relationships between sleep and amyloid-beta in the hippocampus. Neurobiol Learn Mem.

[2] Stower H (2018). Meningeal lymphatics in aging and Alzheimer's disease. Nat Med.

[3] World Alzheimer Report. The state of the art of dementia research: new frontiers Published by Alzheimer's Disease International (ADI), London. September 2018 Copyright © Alzheimer’s Disease International. 2018

[4] Tarasoff-Conway JM, Carare RO, Osorio RS, Glodzik L, Butler T, Fieremans E, Axel L, Rusinek H, Nicholson C, Zlokovic BV, Frangione B, Blennow K, Ménard J, Zetterberg H, Wisniewski T, de Leon MJ (2015). Clearance systems in the brain-implications for Alzheimer disease. Nat Rev Neurol.

[5] Zuroff L, Daley D, Black KL, Koronyo-Hamaoui M (2017). Clearance of cerebral Aβ in Alzheimer's disease: reassessing the role of microglia and monocytes. Cell Mol Life Sci.

[6] Sita G, Hrelia P, Tarozzi A, Morroni F (2017). P-glycoprotein (ABCB1) and oxidative stress: focus on Alzheimer's Disease. Oxid Med Cell Longev.

[7] Bello I, Salerno M (2015). Evidence against a role of P-glycoprotein in the clearance of the Alzheimer's disease Aβ1-42 peptides. Cell Stress Chaperones.

[8] Chai AB, Leung GKF, Callaghan R, Gelissen IC (2020). P-glycoprotein: a role in the export of amyloid-β in Alzheimer's disease?. FEBS J.

[9] Cao L, Zhou J, Wen J (2019). Transport of salvianolic acid B via the human organic anion transporter 1B1 in the liver. Phytother Res.

[10] Wen J, Zhao M, Liu L (2020). Expression of Oatp2 in the brain and liver of Alzheimer disease mouse model. ACS Chem Neurosci.

[11] Chera LM, Jessica EW, Lucia IS, Brittany ND, Thomas GB, Geidy ES (2018). Impaired hepatic amyloid-beta degradation in Alzheimer's disease. PLoS ONE.

[12] Amandi-Burgermeister E, Tibes U, Kaiser BM, Friebe WG, Scheuer WV (1997). Suppression of cytokine synthesis, integrin expression and chronic inflammation by inhibitors of cytosolic phospholipase A2. Eur J Pharmacol.

[13] Wesén E, Jeffries GDM, Dzebo MM, Esbjörner EK (2017). Endocytic uptake of monomeric amyloid-β peptides is clathrin- and dynamin-independent and results in selective accumulation of Aβ(1–42) compared to Aβ(1–40). Sci Rep.

[14] Omtri RS, Davidson MW, Arumugam B, Poduslo JF, Kandimalla KK (2012). Differences in the cellular uptake and intracellular itineraries of amyloid beta proteins 40 and 42: ramifications for the Alzheimer’s drug discovery. Mol Pharm.

[15] Ghiso J, Shayo M, Calero M, Ng D, Tomidokoro Y, Gandy S, Rostagno A, Frangione B (2004). Systemic catabolismof Alzheimer’s Abeta40 and Abeta42. J Biol Chem.

[16] XiangY BuXL, Liu YH, Zhu C, Shen LL, Jiao SS, Zhu XY, Giunta B, Tan J, Song WH, Zhou HD, Zhou XF, Wang YJ (2015). Physiological amyloid-beta clearance in the periphery and its therapeutic potential for Alzheimer’s disease. Acta Neuropathol.

[17] Park DW, Cho HC (2019). Ultrasound shear wave simulation of wave propagation at oblique angles. Australas Phys Eng Sci Med.

