# Publisher Correction to: Uptake of Aβ by OATPs might be a new pathophysiological mechanism of Alzheimer disease

**DOI:** 10.1186/s12868-021-00664-x

**Published:** 2021-09-29

**Authors:** Jinhua Wen, Menghua Zhao, Wenxiong Sun, Xiaohua Cheng, Luyi Yu, Duanwen Cao, Pu Li

**Affiliations:** 1 Departmentof GCP/Psychosomatic Medicine, The First Affiliated Hospital of Nanchang University, Nanchang, China; 2 School of Pharmacy, Nanchang University, Nanchang, China

## Publisher Correction to: BMC Neurosci 22:53 (2021) 10.1186/s12868-021-00658-9

Following publication of the original article [1], it was reported that a co-first authorship designation was missing for Jinhua Wen and Menghua Zhao. The corrected authorship list is available in this Correction article and the original article has been corrected. The publishers apologize for the error.
