# Supplementary figures and images for: Uptake of Aβ by OATPs might be a new pathophysiological mechanism of Alzheimer disease
Source: BMC Neurosci. 2021 Sep 14;22:53. doi: 10.1186/s12868-021-00658-9 (PMC8439072; doi:10.1186/s12868-021-00658-9)

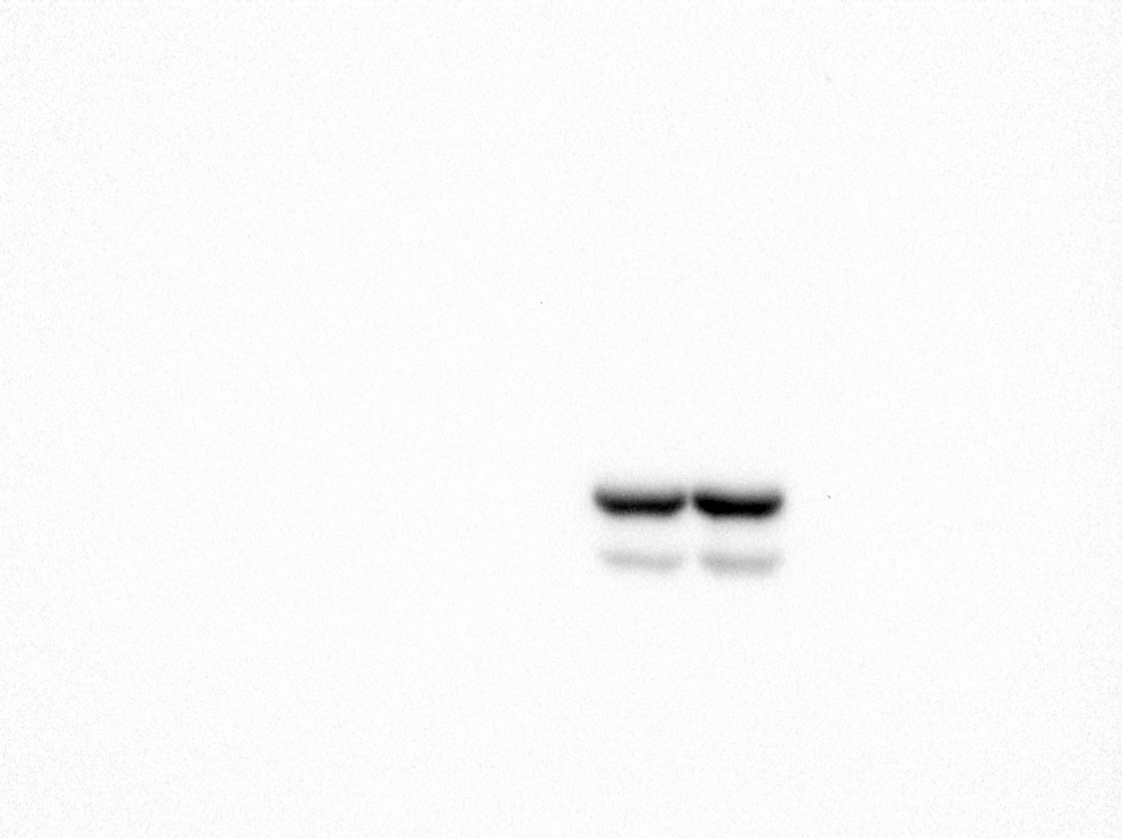

Supplement: Supplementary file 1 — Additional file 1: Raw western blot of actin in Figure 1. [file 12868_2021_658_MOESM1_ESM.tif]

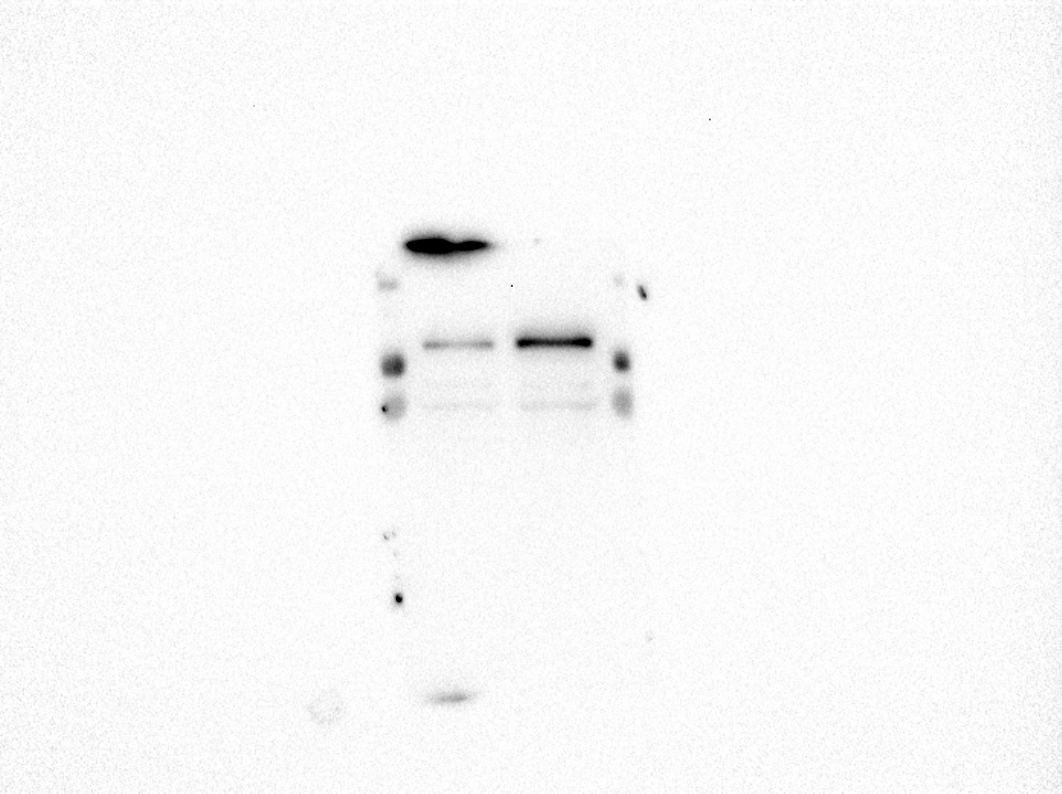

Supplement: Supplementary file 2 — Additional file 2: Raw western blot of OATP1B1 in Figure 1. [file 12868_2021_658_MOESM2_ESM.tif]

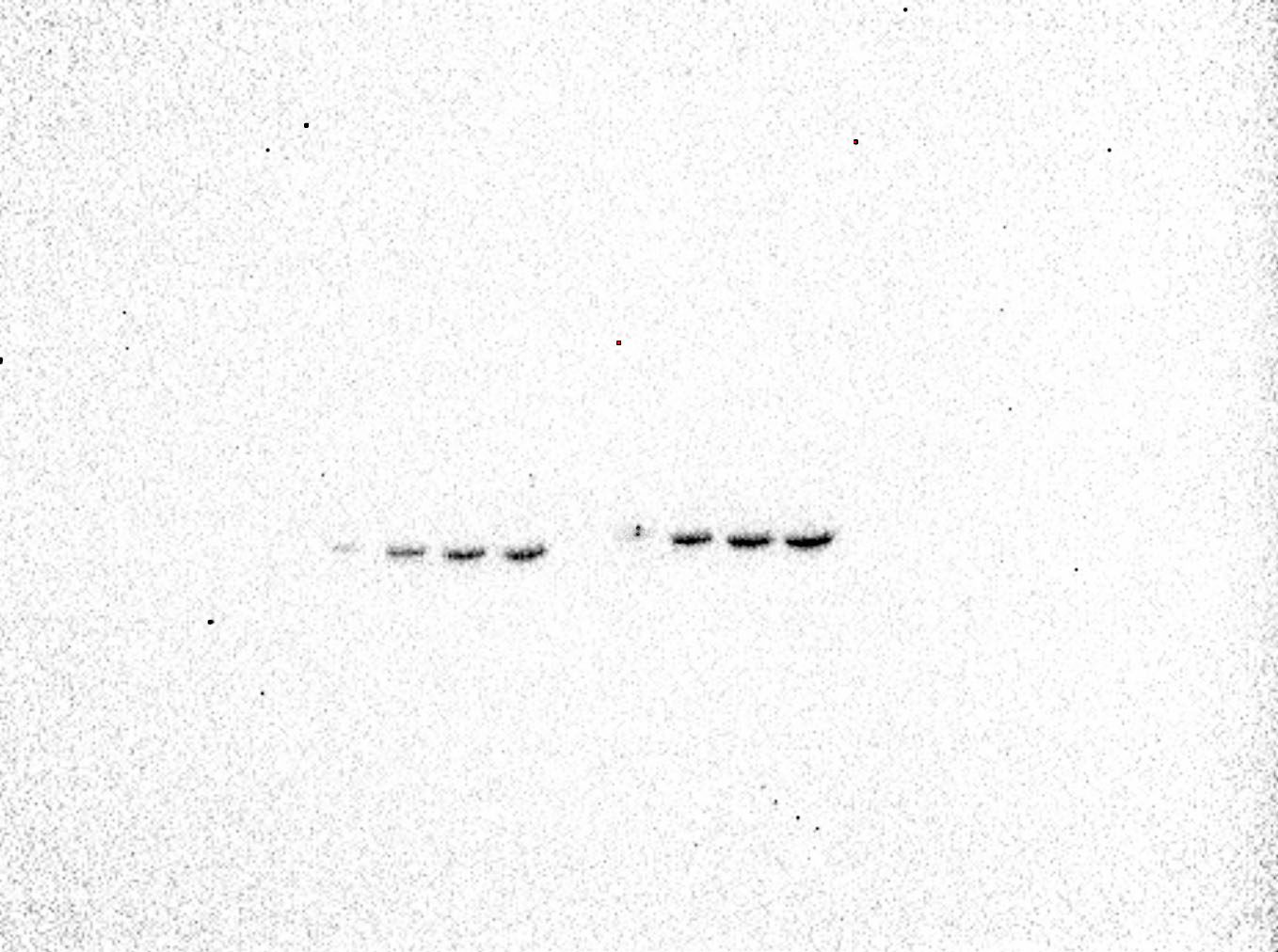

Supplement: Supplementary file 3 — Additional file 3: Raw western blot of Aβ1-42 after incubation for 24h in Figure 2. [file 12868_2021_658_MOESM3_ESM.tif]

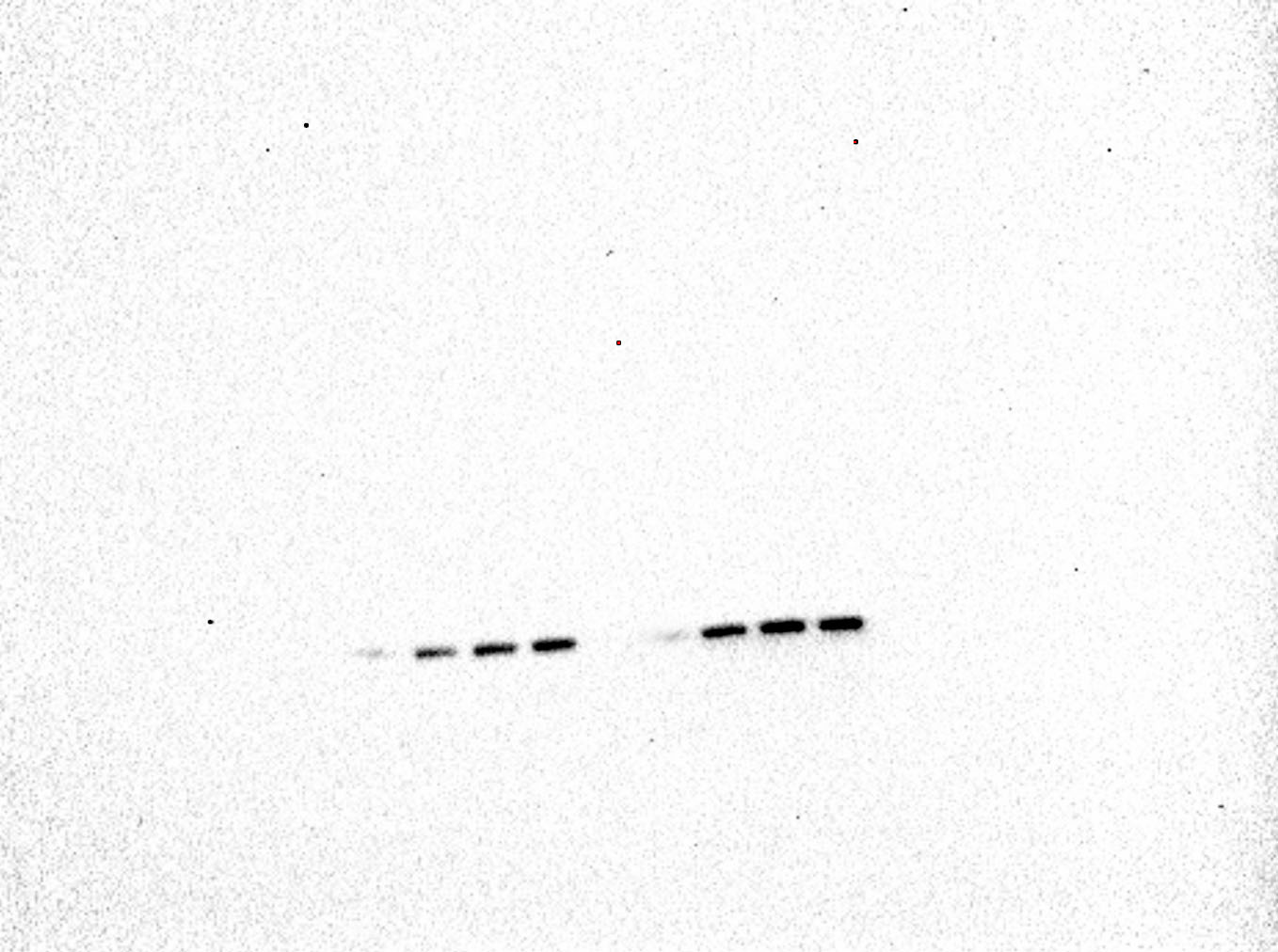

Supplement: Supplementary file 4 — Additional file 4: Raw western blot of Aβ1-42 after incubation for 48h in Figure 2. [file 12868_2021_658_MOESM4_ESM.tif]

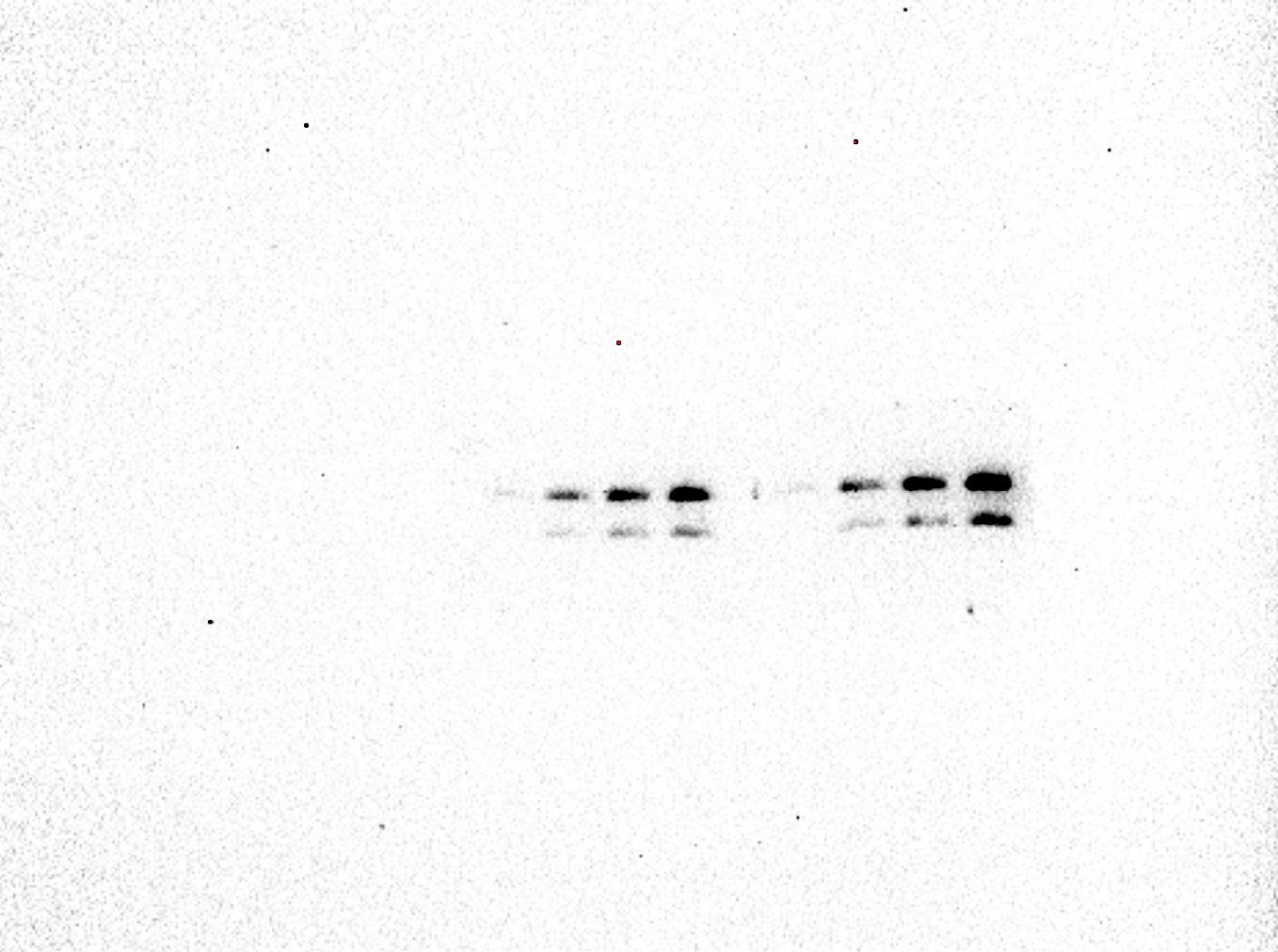

Supplement: Supplementary file 5 — Additional file 5: Raw western blot of Aβ1-42 after incubation for 72h in Figure 2. [file 12868_2021_658_MOESM5_ESM.tif]

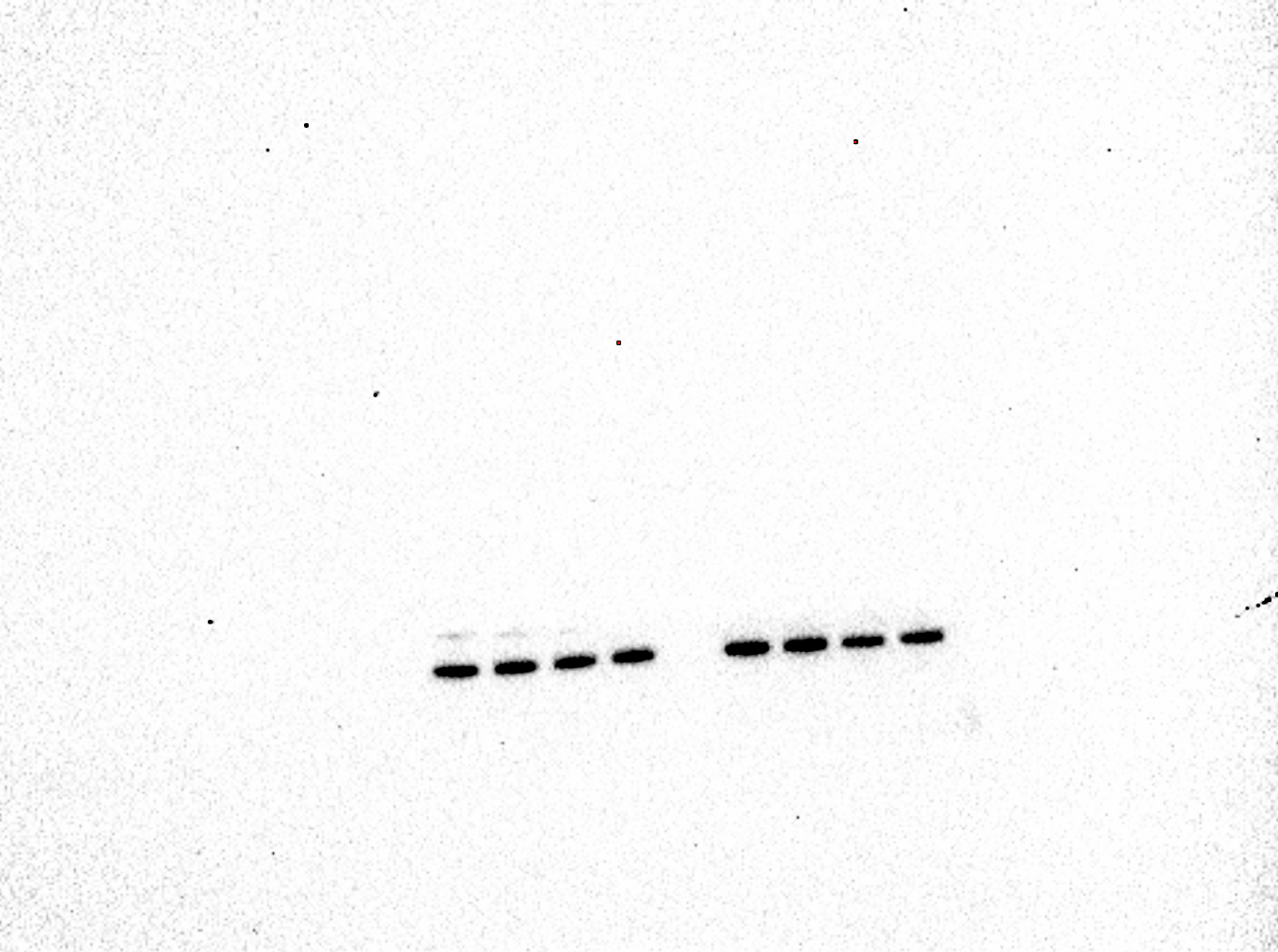

Supplement: Supplementary file 6 — Additional file 6: Raw western blot of βactin after incubation for 24h in Figure 2. [file 12868_2021_658_MOESM6_ESM.tif]

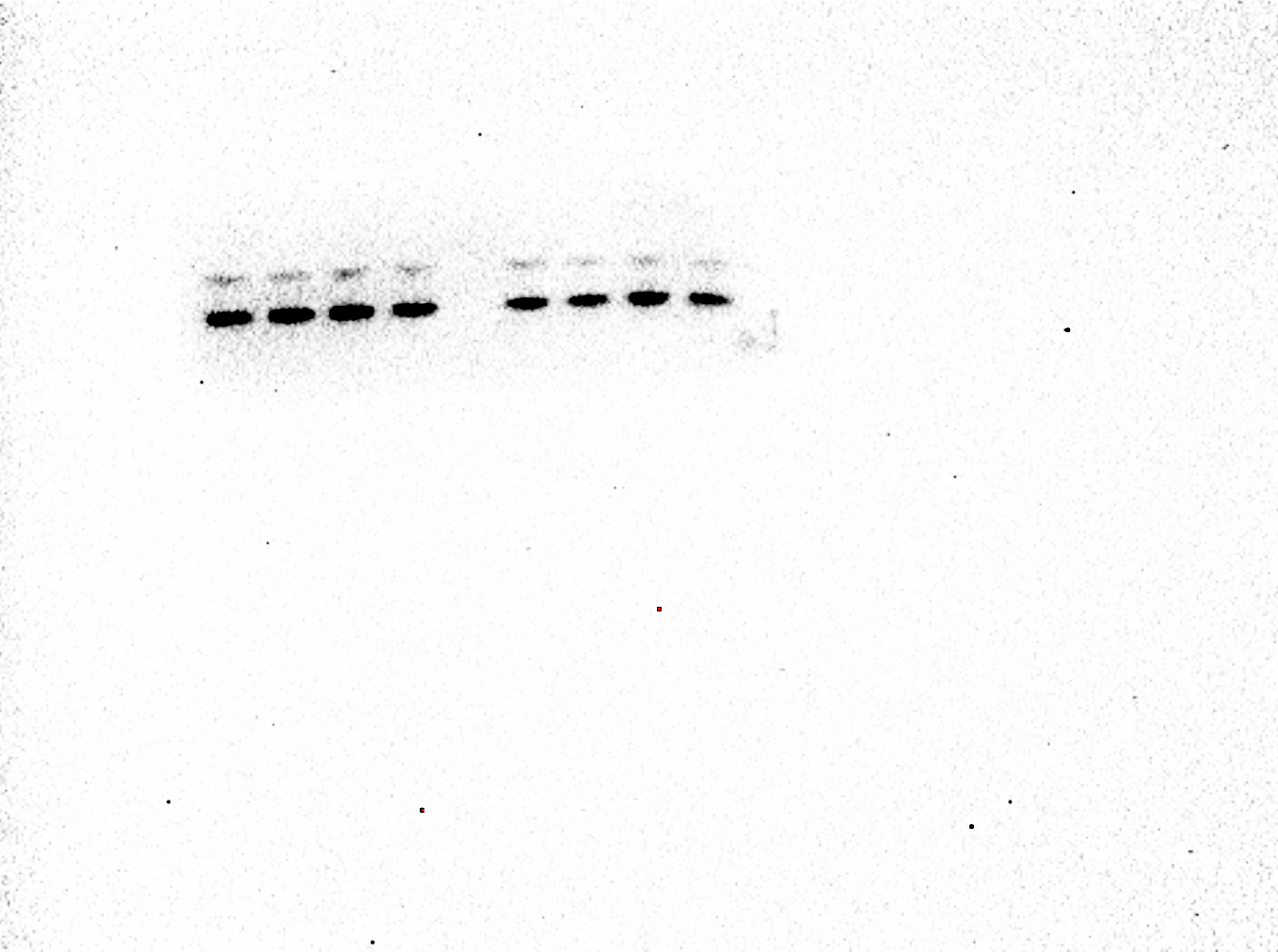

Supplement: Supplementary file 7 — Additional file 7: Raw western blot of βactin after incubation for 48h in Figure 2. [file 12868_2021_658_MOESM7_ESM.tif]

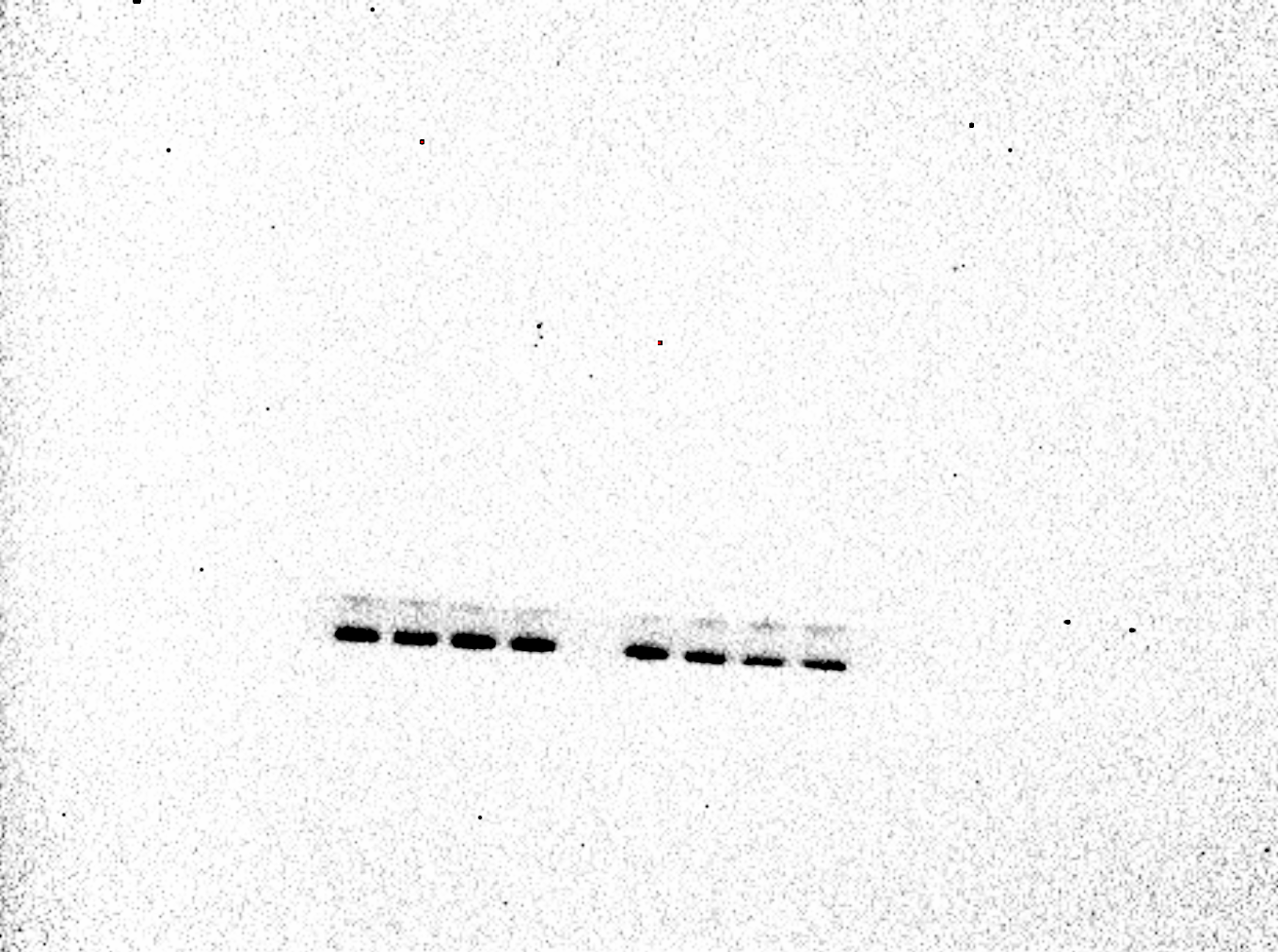

Supplement: Supplementary file 8 — Additional file 8: Raw western blot of βactin after incubation for 72h in Figure 2. [file 12868_2021_658_MOESM8_ESM.tif]

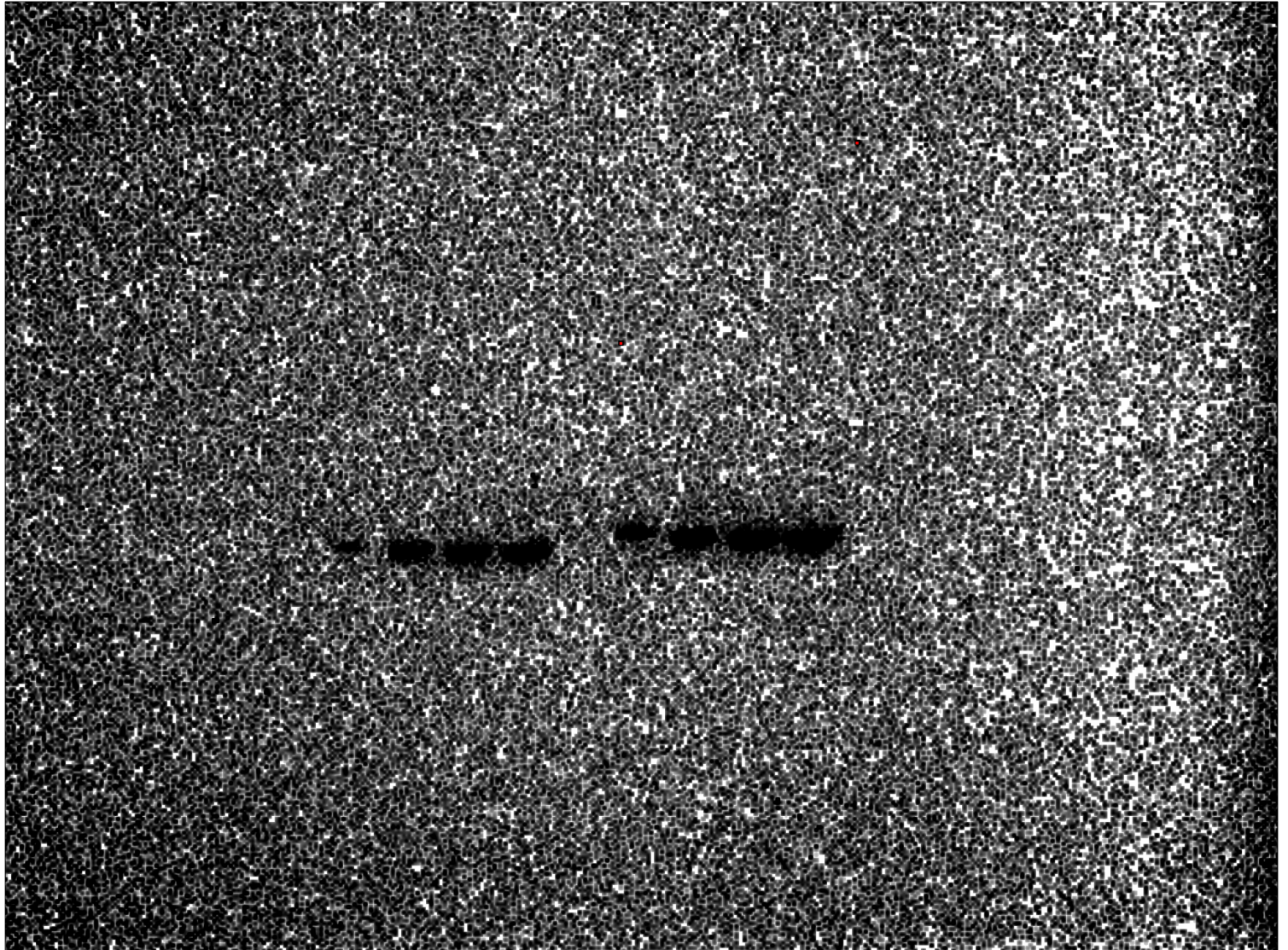

Supplement: Supplementary file 13 — Additional file 13: multiple exposures-Figure2 -Aβ1-42-24h Administrator 2020-05-22 13 h 48 min_Exposure_50.0sec. multiple exposures-Figure2 -Aβ1-42-24h Administrator 2020-05-22 13 h 48 min_Exposure_75.9sec. multiple exposures-Figure2 -Aβ1-42-24h Administrator 2020-05-22 13 h 48 min_Exposure_300.0sec. multiple exposures-Figure2 -Aβ1-42-48h Administrator 2020-05-22 13 h 33 min_Exposure_50.0sec. multiple exposures-Figure2 -Aβ1-42-48h Administrator 2020-05-22 13 h 33 min_Exposure_205.2sec. multiple exposures-Figure2 -Aβ1-42-48h Administrator 2020-05-22 13 h 33 min_Exposure_291.4sec. multiple exposures-Figure2 -Aβ1-42-72h Administrator 2020-05-22 11 h 17 min_Exposure_58.6sec. multiple exposures-Figure2 -Aβ1-42-72h Administrator 2020-05-22 11 h 17 min_Exposure_231.0sec. multiple exposures-Figure2 -Aβ1-42-72h Administrator 2020-05-22 11 h 17 min_Exposure_300.0sec. multiple exposures-Figure2 -βactin-24h Administrator 2020-05-22 11 h 36 min_Exposure_50.0sec. multiple exposures-Figure2 -βactin-24h Administrator 2020-05-22 11 h 36 min_Exposure_239.6sec. multiple exposures-Figure2 -βactin-24h Administrator 2020-05-22 11 h 36 min_Exposure_300.0sec. multiple exposures-Figure2 -βactin-48h Administrator 2020-05-22 10 h 58 min_Exposure_50.0sec. multiple exposures-Figure2 -βactin-48h Administrator 2020-05-22 10 h 58 min_Exposure_282.7sec. multiple exposures-Figure2 -βactin-48h Administrator 2020-05-22 11 h 58 min_Exposure_300.0sec. multiple exposures-Figure2 -βactin-72h Administrator 2020-05-22 11 h 50 min_Exposure_50.0sec. multiple exposures-Figure2 -βactin-72h Administrator 2020-05-22 11 h 50 min_Exposure_205.2sec. multiple exposures-Figure2 -βactin-72h Administrator 2020-05-22 11 h 50 min_Exposure_300.0sec. [file 12868_2021_658_MOESM13_ESM.zip › multiple exposures/multiple exposures-Figure2 -Aa┬1-42-24h Administrator 2020-05-22 13 ╩▒ 48 ╖╓_Exposure_300.0sec.pdf]

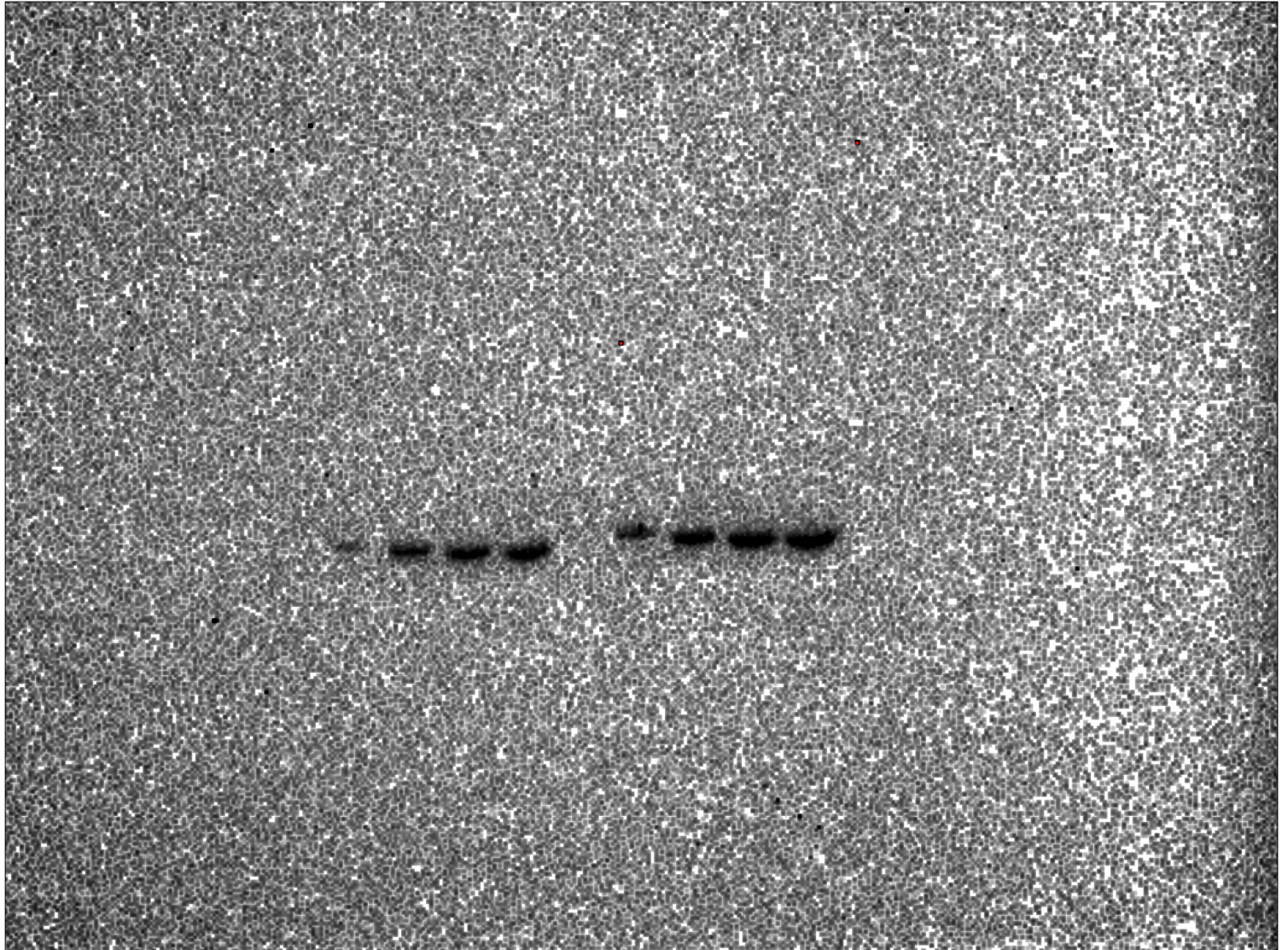

Supplement: Supplementary file 13 — Additional file 13: multiple exposures-Figure2 -Aβ1-42-24h Administrator 2020-05-22 13 h 48 min_Exposure_50.0sec. multiple exposures-Figure2 -Aβ1-42-24h Administrator 2020-05-22 13 h 48 min_Exposure_75.9sec. multiple exposures-Figure2 -Aβ1-42-24h Administrator 2020-05-22 13 h 48 min_Exposure_300.0sec. multiple exposures-Figure2 -Aβ1-42-48h Administrator 2020-05-22 13 h 33 min_Exposure_50.0sec. multiple exposures-Figure2 -Aβ1-42-48h Administrator 2020-05-22 13 h 33 min_Exposure_205.2sec. multiple exposures-Figure2 -Aβ1-42-48h Administrator 2020-05-22 13 h 33 min_Exposure_291.4sec. multiple exposures-Figure2 -Aβ1-42-72h Administrator 2020-05-22 11 h 17 min_Exposure_58.6sec. multiple exposures-Figure2 -Aβ1-42-72h Administrator 2020-05-22 11 h 17 min_Exposure_231.0sec. multiple exposures-Figure2 -Aβ1-42-72h Administrator 2020-05-22 11 h 17 min_Exposure_300.0sec. multiple exposures-Figure2 -βactin-24h Administrator 2020-05-22 11 h 36 min_Exposure_50.0sec. multiple exposures-Figure2 -βactin-24h Administrator 2020-05-22 11 h 36 min_Exposure_239.6sec. multiple exposures-Figure2 -βactin-24h Administrator 2020-05-22 11 h 36 min_Exposure_300.0sec. multiple exposures-Figure2 -βactin-48h Administrator 2020-05-22 10 h 58 min_Exposure_50.0sec. multiple exposures-Figure2 -βactin-48h Administrator 2020-05-22 10 h 58 min_Exposure_282.7sec. multiple exposures-Figure2 -βactin-48h Administrator 2020-05-22 11 h 58 min_Exposure_300.0sec. multiple exposures-Figure2 -βactin-72h Administrator 2020-05-22 11 h 50 min_Exposure_50.0sec. multiple exposures-Figure2 -βactin-72h Administrator 2020-05-22 11 h 50 min_Exposure_205.2sec. multiple exposures-Figure2 -βactin-72h Administrator 2020-05-22 11 h 50 min_Exposure_300.0sec. [file 12868_2021_658_MOESM13_ESM.zip › multiple exposures/multiple exposures-Figure2 -Aa┬1-42-24h Administrator 2020-05-22 13 ╩▒ 48 ╖╓_Exposure_50.0sec.pdf]

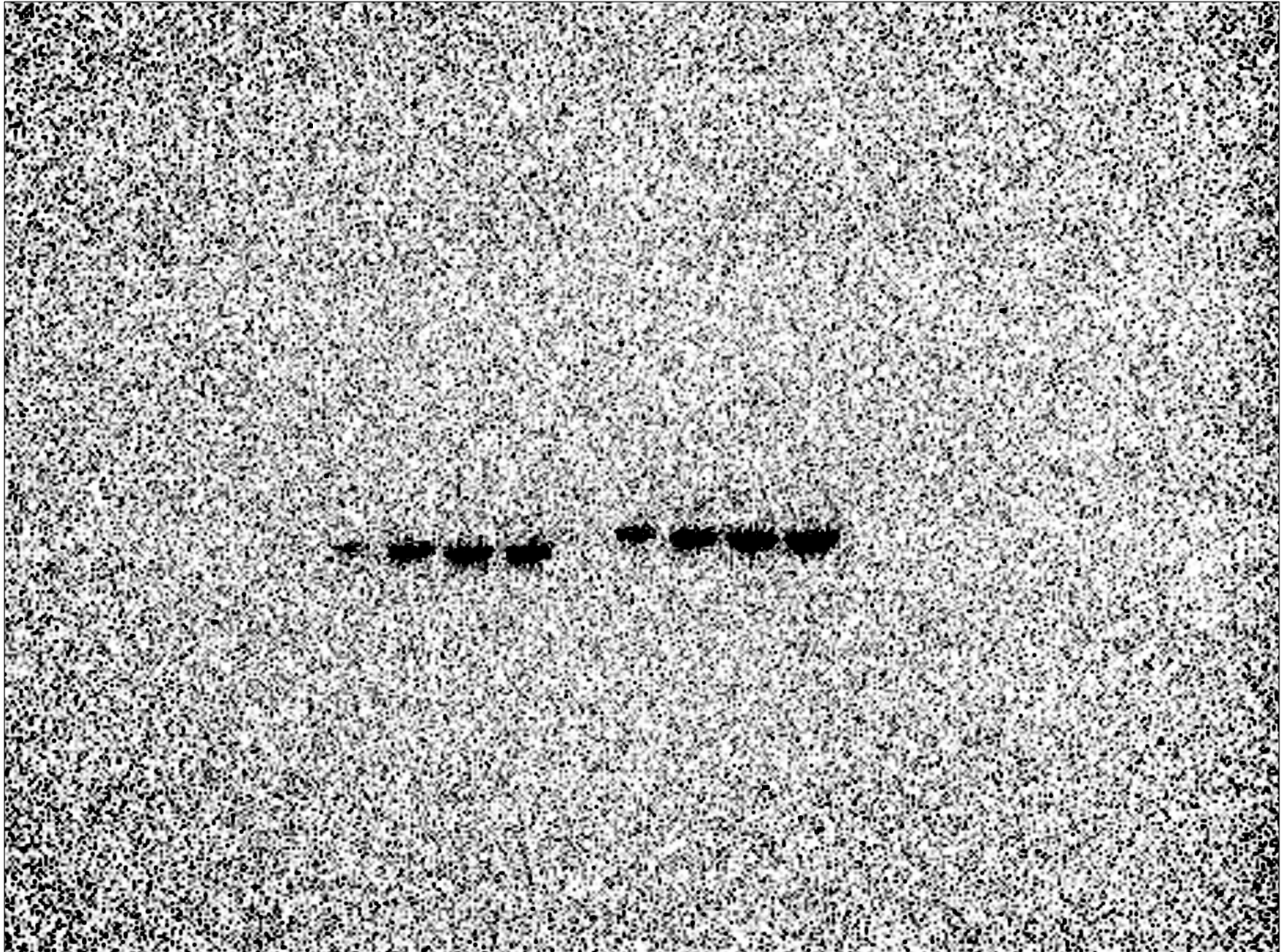

Supplement: Supplementary file 13 — Additional file 13: multiple exposures-Figure2 -Aβ1-42-24h Administrator 2020-05-22 13 h 48 min_Exposure_50.0sec. multiple exposures-Figure2 -Aβ1-42-24h Administrator 2020-05-22 13 h 48 min_Exposure_75.9sec. multiple exposures-Figure2 -Aβ1-42-24h Administrator 2020-05-22 13 h 48 min_Exposure_300.0sec. multiple exposures-Figure2 -Aβ1-42-48h Administrator 2020-05-22 13 h 33 min_Exposure_50.0sec. multiple exposures-Figure2 -Aβ1-42-48h Administrator 2020-05-22 13 h 33 min_Exposure_205.2sec. multiple exposures-Figure2 -Aβ1-42-48h Administrator 2020-05-22 13 h 33 min_Exposure_291.4sec. multiple exposures-Figure2 -Aβ1-42-72h Administrator 2020-05-22 11 h 17 min_Exposure_58.6sec. multiple exposures-Figure2 -Aβ1-42-72h Administrator 2020-05-22 11 h 17 min_Exposure_231.0sec. multiple exposures-Figure2 -Aβ1-42-72h Administrator 2020-05-22 11 h 17 min_Exposure_300.0sec. multiple exposures-Figure2 -βactin-24h Administrator 2020-05-22 11 h 36 min_Exposure_50.0sec. multiple exposures-Figure2 -βactin-24h Administrator 2020-05-22 11 h 36 min_Exposure_239.6sec. multiple exposures-Figure2 -βactin-24h Administrator 2020-05-22 11 h 36 min_Exposure_300.0sec. multiple exposures-Figure2 -βactin-48h Administrator 2020-05-22 10 h 58 min_Exposure_50.0sec. multiple exposures-Figure2 -βactin-48h Administrator 2020-05-22 10 h 58 min_Exposure_282.7sec. multiple exposures-Figure2 -βactin-48h Administrator 2020-05-22 11 h 58 min_Exposure_300.0sec. multiple exposures-Figure2 -βactin-72h Administrator 2020-05-22 11 h 50 min_Exposure_50.0sec. multiple exposures-Figure2 -βactin-72h Administrator 2020-05-22 11 h 50 min_Exposure_205.2sec. multiple exposures-Figure2 -βactin-72h Administrator 2020-05-22 11 h 50 min_Exposure_300.0sec. [file 12868_2021_658_MOESM13_ESM.zip › multiple exposures/multiple exposures-Figure2 -Aa┬1-42-24h Administrator 2020-05-22 13 ╩▒ 48 ╖╓_Exposure_75.9sec.pdf]

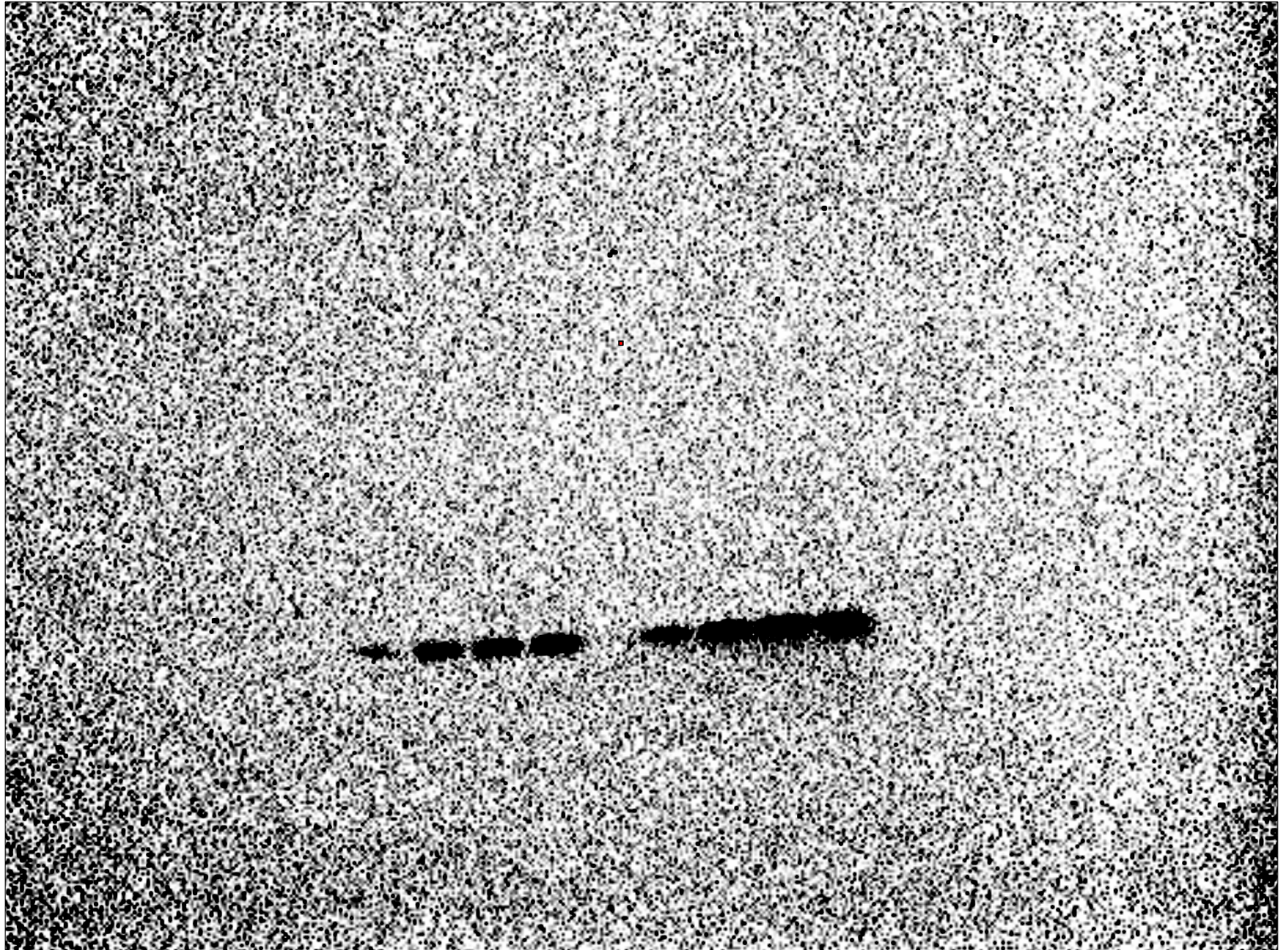

Supplement: Supplementary file 13 — Additional file 13: multiple exposures-Figure2 -Aβ1-42-24h Administrator 2020-05-22 13 h 48 min_Exposure_50.0sec. multiple exposures-Figure2 -Aβ1-42-24h Administrator 2020-05-22 13 h 48 min_Exposure_75.9sec. multiple exposures-Figure2 -Aβ1-42-24h Administrator 2020-05-22 13 h 48 min_Exposure_300.0sec. multiple exposures-Figure2 -Aβ1-42-48h Administrator 2020-05-22 13 h 33 min_Exposure_50.0sec. multiple exposures-Figure2 -Aβ1-42-48h Administrator 2020-05-22 13 h 33 min_Exposure_205.2sec. multiple exposures-Figure2 -Aβ1-42-48h Administrator 2020-05-22 13 h 33 min_Exposure_291.4sec. multiple exposures-Figure2 -Aβ1-42-72h Administrator 2020-05-22 11 h 17 min_Exposure_58.6sec. multiple exposures-Figure2 -Aβ1-42-72h Administrator 2020-05-22 11 h 17 min_Exposure_231.0sec. multiple exposures-Figure2 -Aβ1-42-72h Administrator 2020-05-22 11 h 17 min_Exposure_300.0sec. multiple exposures-Figure2 -βactin-24h Administrator 2020-05-22 11 h 36 min_Exposure_50.0sec. multiple exposures-Figure2 -βactin-24h Administrator 2020-05-22 11 h 36 min_Exposure_239.6sec. multiple exposures-Figure2 -βactin-24h Administrator 2020-05-22 11 h 36 min_Exposure_300.0sec. multiple exposures-Figure2 -βactin-48h Administrator 2020-05-22 10 h 58 min_Exposure_50.0sec. multiple exposures-Figure2 -βactin-48h Administrator 2020-05-22 10 h 58 min_Exposure_282.7sec. multiple exposures-Figure2 -βactin-48h Administrator 2020-05-22 11 h 58 min_Exposure_300.0sec. multiple exposures-Figure2 -βactin-72h Administrator 2020-05-22 11 h 50 min_Exposure_50.0sec. multiple exposures-Figure2 -βactin-72h Administrator 2020-05-22 11 h 50 min_Exposure_205.2sec. multiple exposures-Figure2 -βactin-72h Administrator 2020-05-22 11 h 50 min_Exposure_300.0sec. [file 12868_2021_658_MOESM13_ESM.zip › multiple exposures/multiple exposures-Figure2 -Aa┬1-42-48h Administrator 2020-05-22 13 ╩▒ 33 ╖╓_Exposure_205.2sec.pdf]

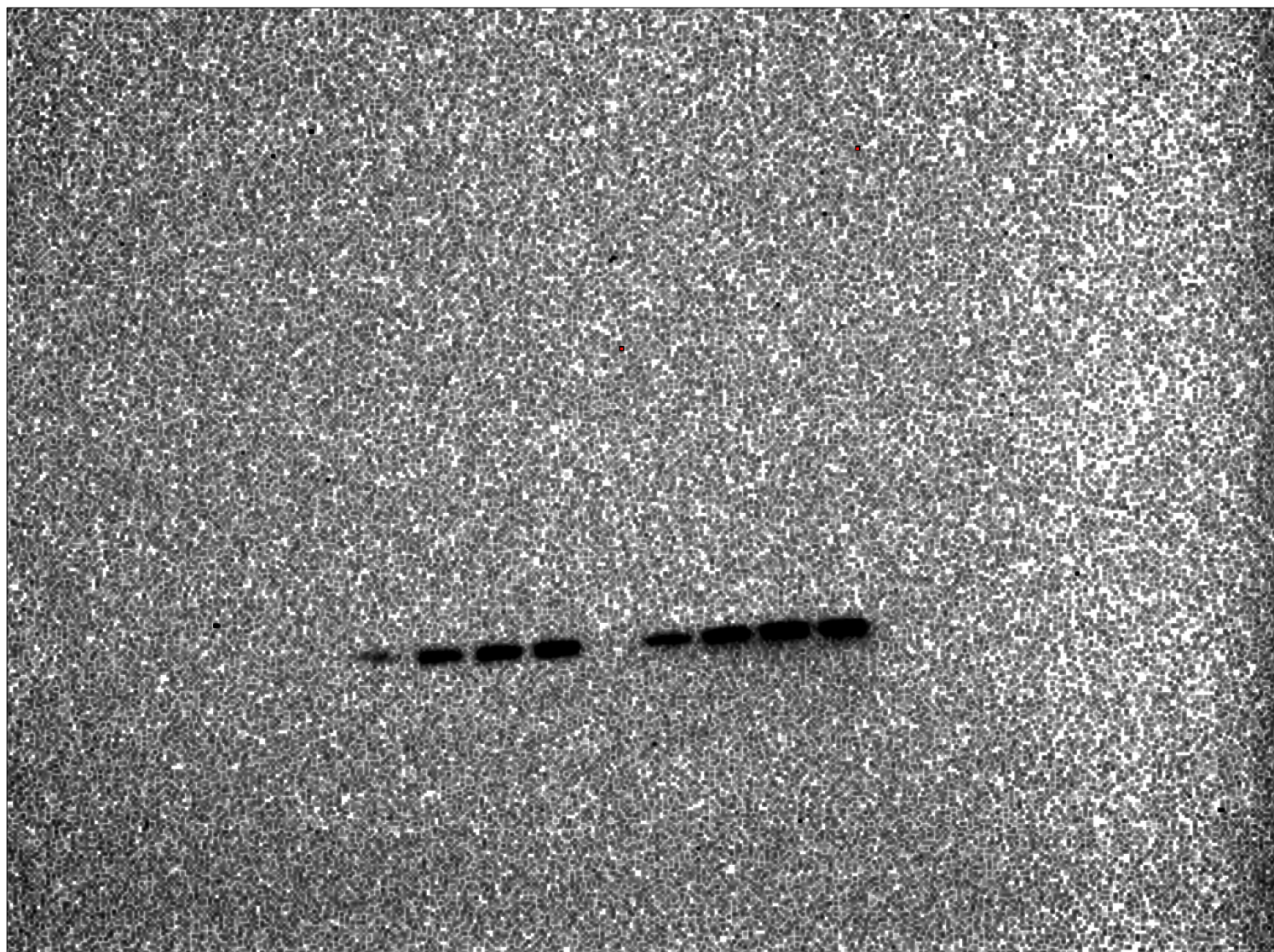

Supplement: Supplementary file 13 — Additional file 13: multiple exposures-Figure2 -Aβ1-42-24h Administrator 2020-05-22 13 h 48 min_Exposure_50.0sec. multiple exposures-Figure2 -Aβ1-42-24h Administrator 2020-05-22 13 h 48 min_Exposure_75.9sec. multiple exposures-Figure2 -Aβ1-42-24h Administrator 2020-05-22 13 h 48 min_Exposure_300.0sec. multiple exposures-Figure2 -Aβ1-42-48h Administrator 2020-05-22 13 h 33 min_Exposure_50.0sec. multiple exposures-Figure2 -Aβ1-42-48h Administrator 2020-05-22 13 h 33 min_Exposure_205.2sec. multiple exposures-Figure2 -Aβ1-42-48h Administrator 2020-05-22 13 h 33 min_Exposure_291.4sec. multiple exposures-Figure2 -Aβ1-42-72h Administrator 2020-05-22 11 h 17 min_Exposure_58.6sec. multiple exposures-Figure2 -Aβ1-42-72h Administrator 2020-05-22 11 h 17 min_Exposure_231.0sec. multiple exposures-Figure2 -Aβ1-42-72h Administrator 2020-05-22 11 h 17 min_Exposure_300.0sec. multiple exposures-Figure2 -βactin-24h Administrator 2020-05-22 11 h 36 min_Exposure_50.0sec. multiple exposures-Figure2 -βactin-24h Administrator 2020-05-22 11 h 36 min_Exposure_239.6sec. multiple exposures-Figure2 -βactin-24h Administrator 2020-05-22 11 h 36 min_Exposure_300.0sec. multiple exposures-Figure2 -βactin-48h Administrator 2020-05-22 10 h 58 min_Exposure_50.0sec. multiple exposures-Figure2 -βactin-48h Administrator 2020-05-22 10 h 58 min_Exposure_282.7sec. multiple exposures-Figure2 -βactin-48h Administrator 2020-05-22 11 h 58 min_Exposure_300.0sec. multiple exposures-Figure2 -βactin-72h Administrator 2020-05-22 11 h 50 min_Exposure_50.0sec. multiple exposures-Figure2 -βactin-72h Administrator 2020-05-22 11 h 50 min_Exposure_205.2sec. multiple exposures-Figure2 -βactin-72h Administrator 2020-05-22 11 h 50 min_Exposure_300.0sec. [file 12868_2021_658_MOESM13_ESM.zip › multiple exposures/multiple exposures-Figure2 -Aa┬1-42-48h Administrator 2020-05-22 13 ╩▒ 33 ╖╓_Exposure_291.4sec.pdf]

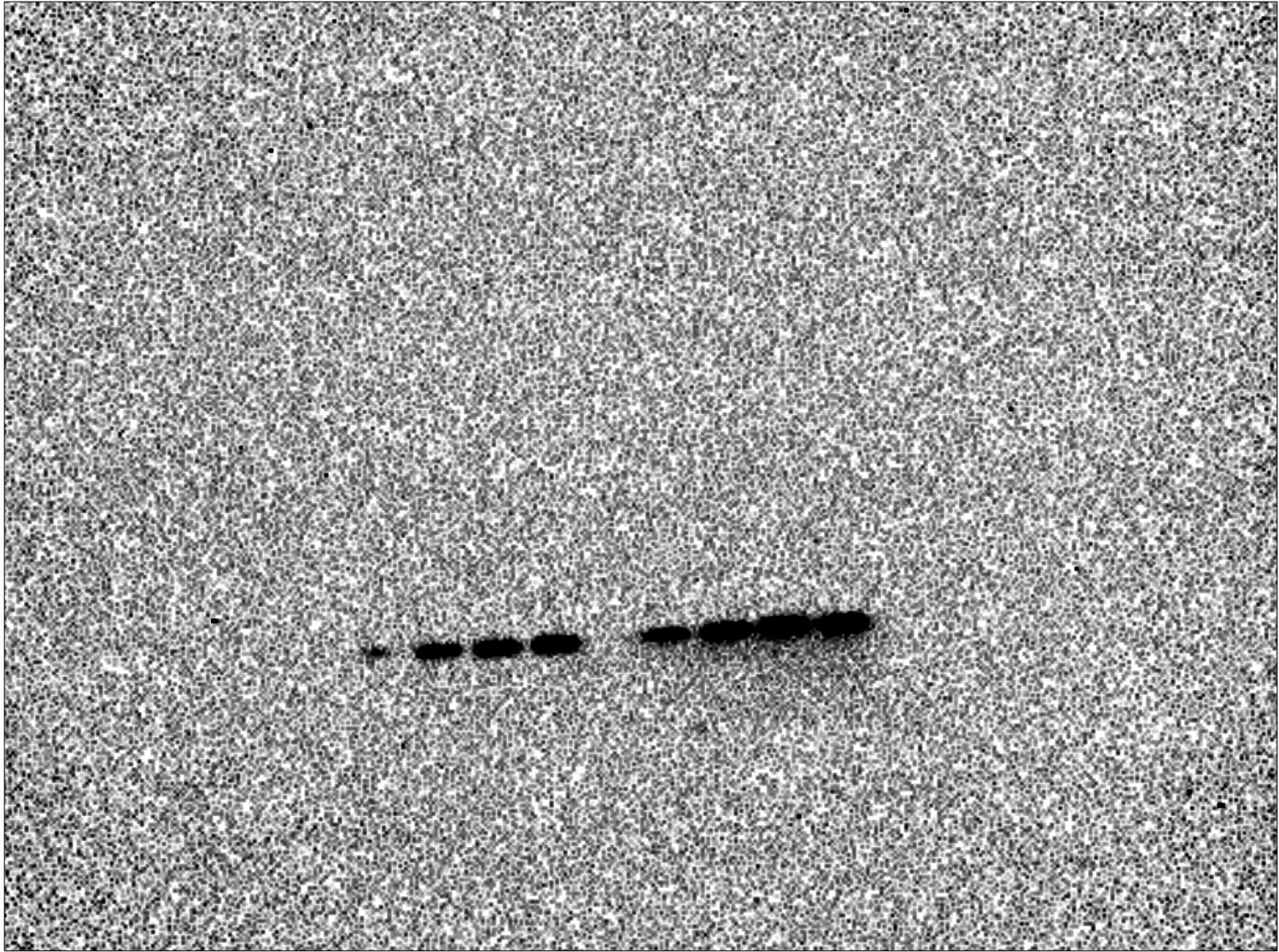

Supplement: Supplementary file 13 — Additional file 13: multiple exposures-Figure2 -Aβ1-42-24h Administrator 2020-05-22 13 h 48 min_Exposure_50.0sec. multiple exposures-Figure2 -Aβ1-42-24h Administrator 2020-05-22 13 h 48 min_Exposure_75.9sec. multiple exposures-Figure2 -Aβ1-42-24h Administrator 2020-05-22 13 h 48 min_Exposure_300.0sec. multiple exposures-Figure2 -Aβ1-42-48h Administrator 2020-05-22 13 h 33 min_Exposure_50.0sec. multiple exposures-Figure2 -Aβ1-42-48h Administrator 2020-05-22 13 h 33 min_Exposure_205.2sec. multiple exposures-Figure2 -Aβ1-42-48h Administrator 2020-05-22 13 h 33 min_Exposure_291.4sec. multiple exposures-Figure2 -Aβ1-42-72h Administrator 2020-05-22 11 h 17 min_Exposure_58.6sec. multiple exposures-Figure2 -Aβ1-42-72h Administrator 2020-05-22 11 h 17 min_Exposure_231.0sec. multiple exposures-Figure2 -Aβ1-42-72h Administrator 2020-05-22 11 h 17 min_Exposure_300.0sec. multiple exposures-Figure2 -βactin-24h Administrator 2020-05-22 11 h 36 min_Exposure_50.0sec. multiple exposures-Figure2 -βactin-24h Administrator 2020-05-22 11 h 36 min_Exposure_239.6sec. multiple exposures-Figure2 -βactin-24h Administrator 2020-05-22 11 h 36 min_Exposure_300.0sec. multiple exposures-Figure2 -βactin-48h Administrator 2020-05-22 10 h 58 min_Exposure_50.0sec. multiple exposures-Figure2 -βactin-48h Administrator 2020-05-22 10 h 58 min_Exposure_282.7sec. multiple exposures-Figure2 -βactin-48h Administrator 2020-05-22 11 h 58 min_Exposure_300.0sec. multiple exposures-Figure2 -βactin-72h Administrator 2020-05-22 11 h 50 min_Exposure_50.0sec. multiple exposures-Figure2 -βactin-72h Administrator 2020-05-22 11 h 50 min_Exposure_205.2sec. multiple exposures-Figure2 -βactin-72h Administrator 2020-05-22 11 h 50 min_Exposure_300.0sec. [file 12868_2021_658_MOESM13_ESM.zip › multiple exposures/multiple exposures-Figure2 -Aa┬1-42-48h Administrator 2020-05-22 13 ╩▒ 33 ╖╓_Exposure_50.0sec.pdf]

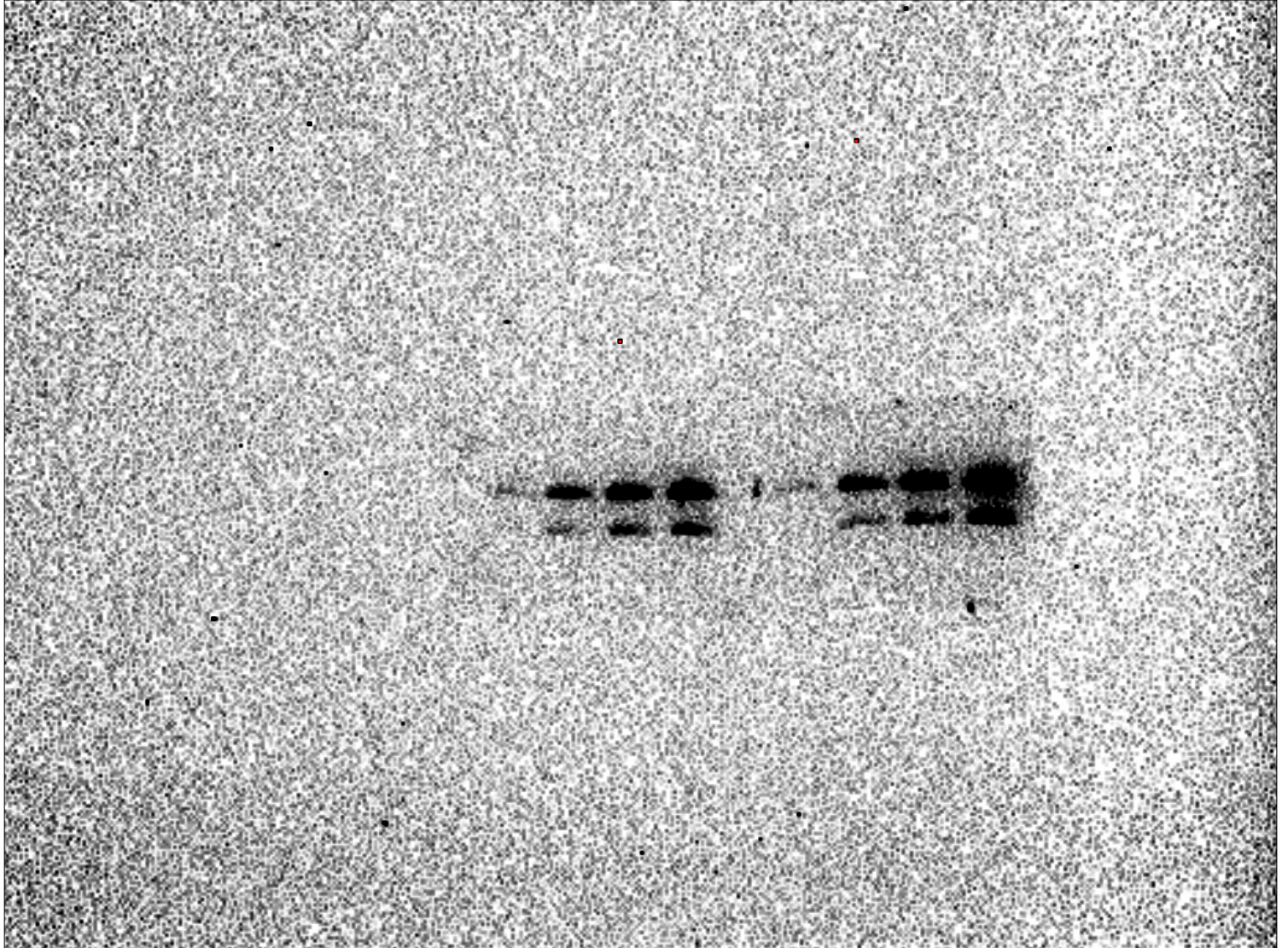

Supplement: Supplementary file 13 — Additional file 13: multiple exposures-Figure2 -Aβ1-42-24h Administrator 2020-05-22 13 h 48 min_Exposure_50.0sec. multiple exposures-Figure2 -Aβ1-42-24h Administrator 2020-05-22 13 h 48 min_Exposure_75.9sec. multiple exposures-Figure2 -Aβ1-42-24h Administrator 2020-05-22 13 h 48 min_Exposure_300.0sec. multiple exposures-Figure2 -Aβ1-42-48h Administrator 2020-05-22 13 h 33 min_Exposure_50.0sec. multiple exposures-Figure2 -Aβ1-42-48h Administrator 2020-05-22 13 h 33 min_Exposure_205.2sec. multiple exposures-Figure2 -Aβ1-42-48h Administrator 2020-05-22 13 h 33 min_Exposure_291.4sec. multiple exposures-Figure2 -Aβ1-42-72h Administrator 2020-05-22 11 h 17 min_Exposure_58.6sec. multiple exposures-Figure2 -Aβ1-42-72h Administrator 2020-05-22 11 h 17 min_Exposure_231.0sec. multiple exposures-Figure2 -Aβ1-42-72h Administrator 2020-05-22 11 h 17 min_Exposure_300.0sec. multiple exposures-Figure2 -βactin-24h Administrator 2020-05-22 11 h 36 min_Exposure_50.0sec. multiple exposures-Figure2 -βactin-24h Administrator 2020-05-22 11 h 36 min_Exposure_239.6sec. multiple exposures-Figure2 -βactin-24h Administrator 2020-05-22 11 h 36 min_Exposure_300.0sec. multiple exposures-Figure2 -βactin-48h Administrator 2020-05-22 10 h 58 min_Exposure_50.0sec. multiple exposures-Figure2 -βactin-48h Administrator 2020-05-22 10 h 58 min_Exposure_282.7sec. multiple exposures-Figure2 -βactin-48h Administrator 2020-05-22 11 h 58 min_Exposure_300.0sec. multiple exposures-Figure2 -βactin-72h Administrator 2020-05-22 11 h 50 min_Exposure_50.0sec. multiple exposures-Figure2 -βactin-72h Administrator 2020-05-22 11 h 50 min_Exposure_205.2sec. multiple exposures-Figure2 -βactin-72h Administrator 2020-05-22 11 h 50 min_Exposure_300.0sec. [file 12868_2021_658_MOESM13_ESM.zip › multiple exposures/multiple exposures-Figure2 -Aa┬1-42-72h Administrator 2020-05-22 11 ╩▒ 17 ╖╓_Exposure_231.0sec.pdf]

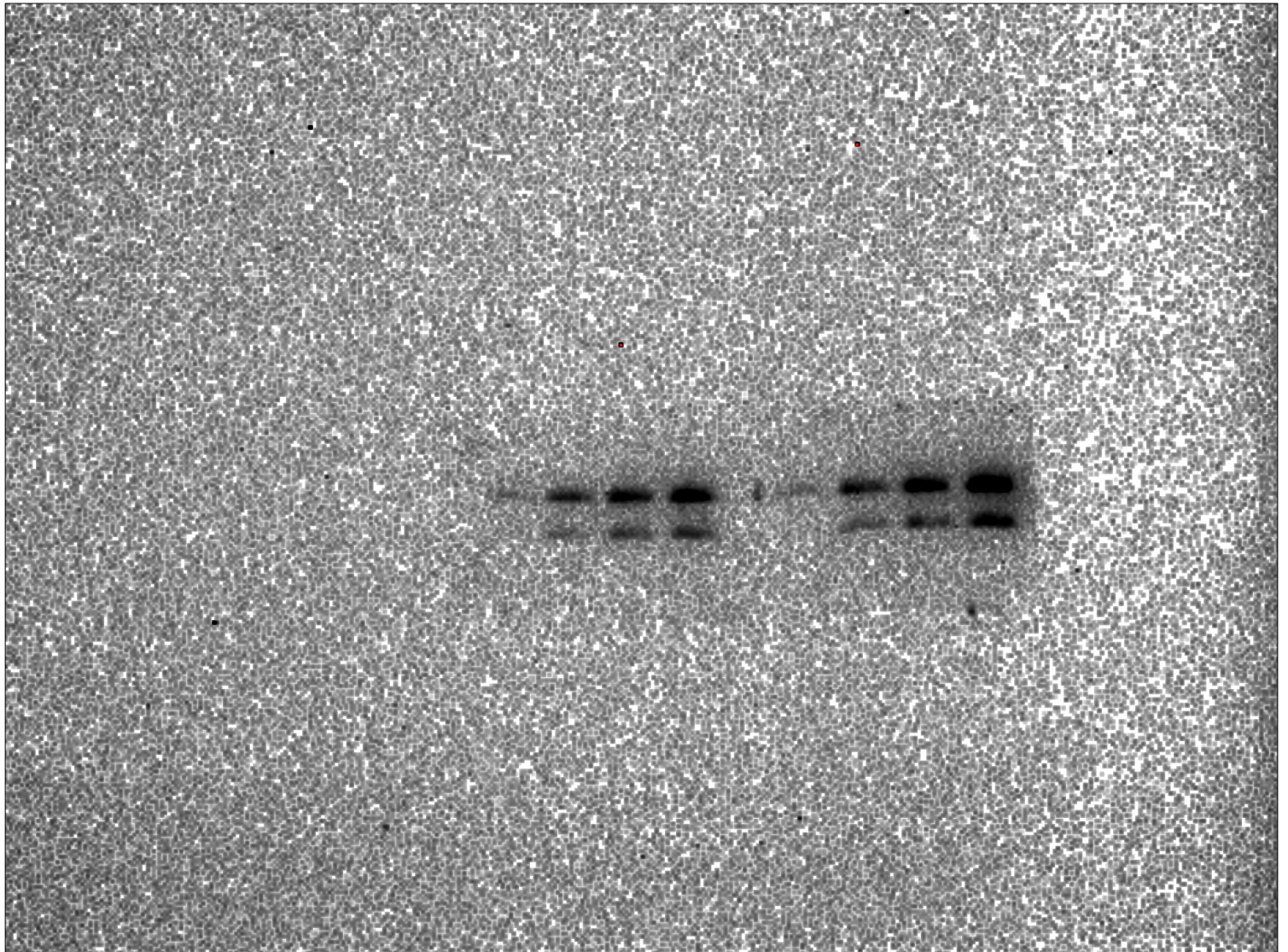

Supplement: Supplementary file 13 — Additional file 13: multiple exposures-Figure2 -Aβ1-42-24h Administrator 2020-05-22 13 h 48 min_Exposure_50.0sec. multiple exposures-Figure2 -Aβ1-42-24h Administrator 2020-05-22 13 h 48 min_Exposure_75.9sec. multiple exposures-Figure2 -Aβ1-42-24h Administrator 2020-05-22 13 h 48 min_Exposure_300.0sec. multiple exposures-Figure2 -Aβ1-42-48h Administrator 2020-05-22 13 h 33 min_Exposure_50.0sec. multiple exposures-Figure2 -Aβ1-42-48h Administrator 2020-05-22 13 h 33 min_Exposure_205.2sec. multiple exposures-Figure2 -Aβ1-42-48h Administrator 2020-05-22 13 h 33 min_Exposure_291.4sec. multiple exposures-Figure2 -Aβ1-42-72h Administrator 2020-05-22 11 h 17 min_Exposure_58.6sec. multiple exposures-Figure2 -Aβ1-42-72h Administrator 2020-05-22 11 h 17 min_Exposure_231.0sec. multiple exposures-Figure2 -Aβ1-42-72h Administrator 2020-05-22 11 h 17 min_Exposure_300.0sec. multiple exposures-Figure2 -βactin-24h Administrator 2020-05-22 11 h 36 min_Exposure_50.0sec. multiple exposures-Figure2 -βactin-24h Administrator 2020-05-22 11 h 36 min_Exposure_239.6sec. multiple exposures-Figure2 -βactin-24h Administrator 2020-05-22 11 h 36 min_Exposure_300.0sec. multiple exposures-Figure2 -βactin-48h Administrator 2020-05-22 10 h 58 min_Exposure_50.0sec. multiple exposures-Figure2 -βactin-48h Administrator 2020-05-22 10 h 58 min_Exposure_282.7sec. multiple exposures-Figure2 -βactin-48h Administrator 2020-05-22 11 h 58 min_Exposure_300.0sec. multiple exposures-Figure2 -βactin-72h Administrator 2020-05-22 11 h 50 min_Exposure_50.0sec. multiple exposures-Figure2 -βactin-72h Administrator 2020-05-22 11 h 50 min_Exposure_205.2sec. multiple exposures-Figure2 -βactin-72h Administrator 2020-05-22 11 h 50 min_Exposure_300.0sec. [file 12868_2021_658_MOESM13_ESM.zip › multiple exposures/multiple exposures-Figure2 -Aa┬1-42-72h Administrator 2020-05-22 11 ╩▒ 17 ╖╓_Exposure_300.0sec.pdf]

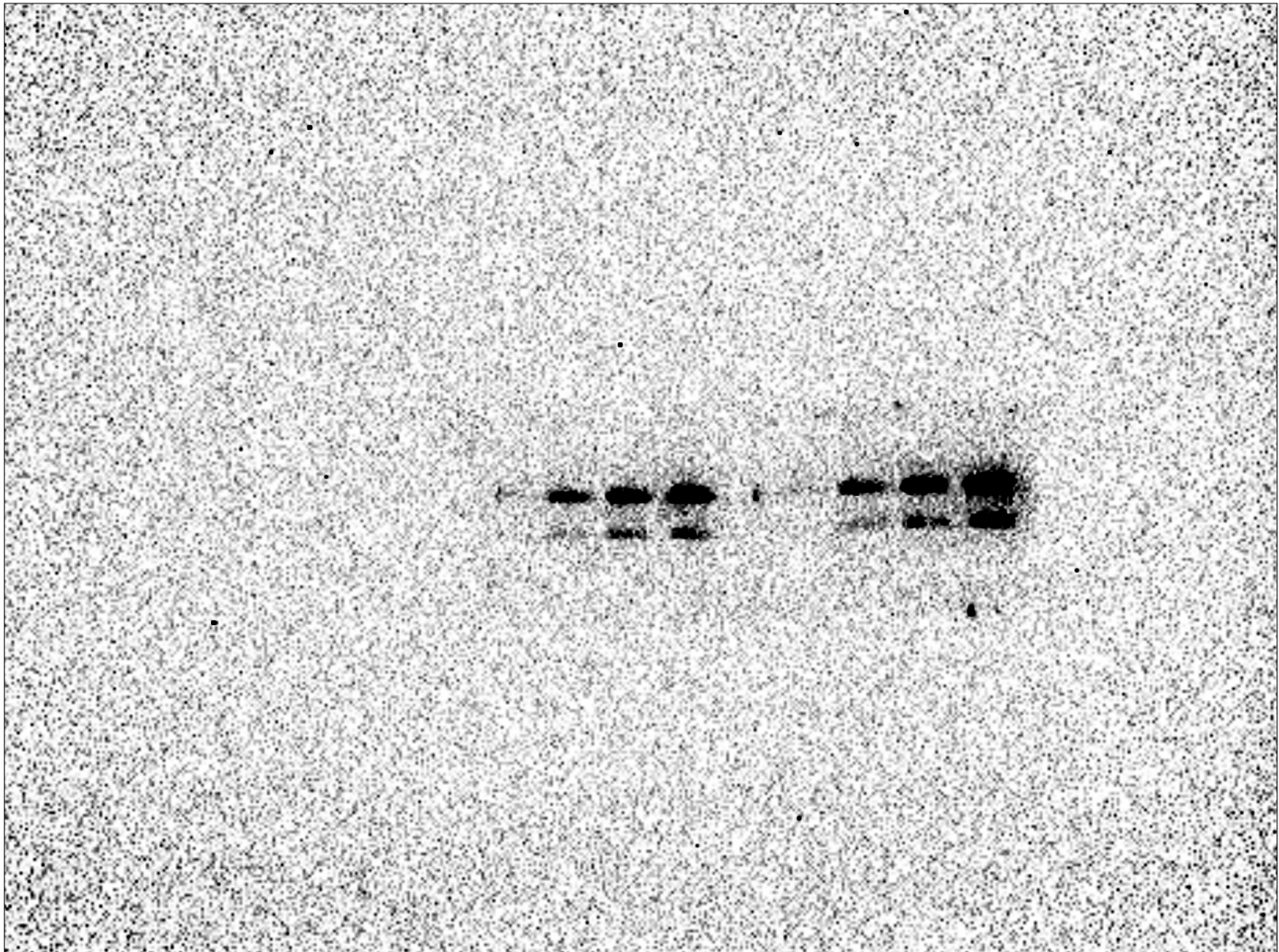

Supplement: Supplementary file 13 — Additional file 13: multiple exposures-Figure2 -Aβ1-42-24h Administrator 2020-05-22 13 h 48 min_Exposure_50.0sec. multiple exposures-Figure2 -Aβ1-42-24h Administrator 2020-05-22 13 h 48 min_Exposure_75.9sec. multiple exposures-Figure2 -Aβ1-42-24h Administrator 2020-05-22 13 h 48 min_Exposure_300.0sec. multiple exposures-Figure2 -Aβ1-42-48h Administrator 2020-05-22 13 h 33 min_Exposure_50.0sec. multiple exposures-Figure2 -Aβ1-42-48h Administrator 2020-05-22 13 h 33 min_Exposure_205.2sec. multiple exposures-Figure2 -Aβ1-42-48h Administrator 2020-05-22 13 h 33 min_Exposure_291.4sec. multiple exposures-Figure2 -Aβ1-42-72h Administrator 2020-05-22 11 h 17 min_Exposure_58.6sec. multiple exposures-Figure2 -Aβ1-42-72h Administrator 2020-05-22 11 h 17 min_Exposure_231.0sec. multiple exposures-Figure2 -Aβ1-42-72h Administrator 2020-05-22 11 h 17 min_Exposure_300.0sec. multiple exposures-Figure2 -βactin-24h Administrator 2020-05-22 11 h 36 min_Exposure_50.0sec. multiple exposures-Figure2 -βactin-24h Administrator 2020-05-22 11 h 36 min_Exposure_239.6sec. multiple exposures-Figure2 -βactin-24h Administrator 2020-05-22 11 h 36 min_Exposure_300.0sec. multiple exposures-Figure2 -βactin-48h Administrator 2020-05-22 10 h 58 min_Exposure_50.0sec. multiple exposures-Figure2 -βactin-48h Administrator 2020-05-22 10 h 58 min_Exposure_282.7sec. multiple exposures-Figure2 -βactin-48h Administrator 2020-05-22 11 h 58 min_Exposure_300.0sec. multiple exposures-Figure2 -βactin-72h Administrator 2020-05-22 11 h 50 min_Exposure_50.0sec. multiple exposures-Figure2 -βactin-72h Administrator 2020-05-22 11 h 50 min_Exposure_205.2sec. multiple exposures-Figure2 -βactin-72h Administrator 2020-05-22 11 h 50 min_Exposure_300.0sec. [file 12868_2021_658_MOESM13_ESM.zip › multiple exposures/multiple exposures-Figure2 -Aa┬1-42-72h Administrator 2020-05-22 11 ╩▒ 17 ╖╓_Exposure_58.6sec.pdf]

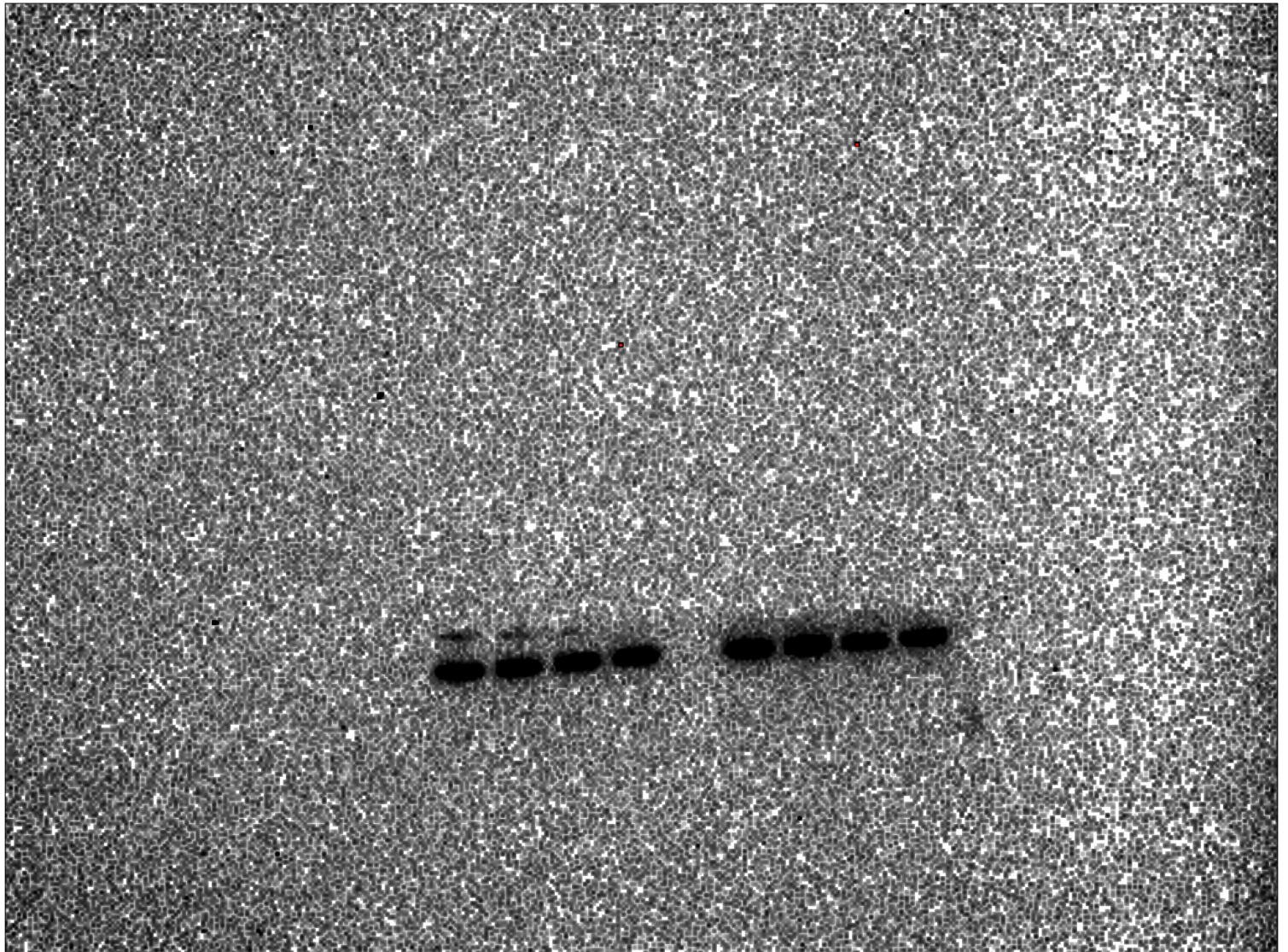

Supplement: Supplementary file 13 — Additional file 13: multiple exposures-Figure2 -Aβ1-42-24h Administrator 2020-05-22 13 h 48 min_Exposure_50.0sec. multiple exposures-Figure2 -Aβ1-42-24h Administrator 2020-05-22 13 h 48 min_Exposure_75.9sec. multiple exposures-Figure2 -Aβ1-42-24h Administrator 2020-05-22 13 h 48 min_Exposure_300.0sec. multiple exposures-Figure2 -Aβ1-42-48h Administrator 2020-05-22 13 h 33 min_Exposure_50.0sec. multiple exposures-Figure2 -Aβ1-42-48h Administrator 2020-05-22 13 h 33 min_Exposure_205.2sec. multiple exposures-Figure2 -Aβ1-42-48h Administrator 2020-05-22 13 h 33 min_Exposure_291.4sec. multiple exposures-Figure2 -Aβ1-42-72h Administrator 2020-05-22 11 h 17 min_Exposure_58.6sec. multiple exposures-Figure2 -Aβ1-42-72h Administrator 2020-05-22 11 h 17 min_Exposure_231.0sec. multiple exposures-Figure2 -Aβ1-42-72h Administrator 2020-05-22 11 h 17 min_Exposure_300.0sec. multiple exposures-Figure2 -βactin-24h Administrator 2020-05-22 11 h 36 min_Exposure_50.0sec. multiple exposures-Figure2 -βactin-24h Administrator 2020-05-22 11 h 36 min_Exposure_239.6sec. multiple exposures-Figure2 -βactin-24h Administrator 2020-05-22 11 h 36 min_Exposure_300.0sec. multiple exposures-Figure2 -βactin-48h Administrator 2020-05-22 10 h 58 min_Exposure_50.0sec. multiple exposures-Figure2 -βactin-48h Administrator 2020-05-22 10 h 58 min_Exposure_282.7sec. multiple exposures-Figure2 -βactin-48h Administrator 2020-05-22 11 h 58 min_Exposure_300.0sec. multiple exposures-Figure2 -βactin-72h Administrator 2020-05-22 11 h 50 min_Exposure_50.0sec. multiple exposures-Figure2 -βactin-72h Administrator 2020-05-22 11 h 50 min_Exposure_205.2sec. multiple exposures-Figure2 -βactin-72h Administrator 2020-05-22 11 h 50 min_Exposure_300.0sec. [file 12868_2021_658_MOESM13_ESM.zip › multiple exposures/multiple exposures-Figure2 -a┬actin-24h Administrator 2020-05-22 11 ╩▒ 36 ╖╓_Exposure_239.6sec.pdf]

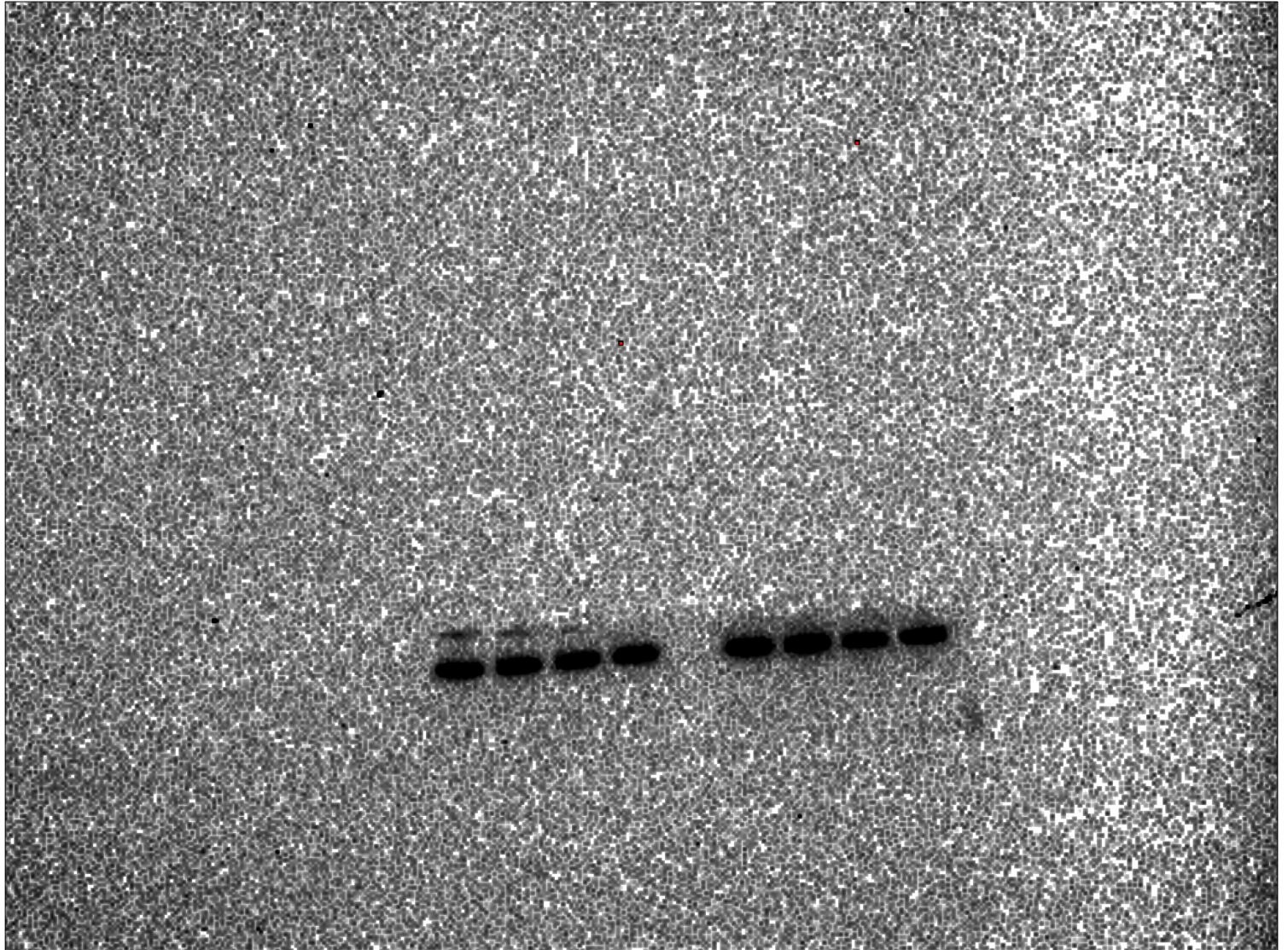

Supplement: Supplementary file 13 — Additional file 13: multiple exposures-Figure2 -Aβ1-42-24h Administrator 2020-05-22 13 h 48 min_Exposure_50.0sec. multiple exposures-Figure2 -Aβ1-42-24h Administrator 2020-05-22 13 h 48 min_Exposure_75.9sec. multiple exposures-Figure2 -Aβ1-42-24h Administrator 2020-05-22 13 h 48 min_Exposure_300.0sec. multiple exposures-Figure2 -Aβ1-42-48h Administrator 2020-05-22 13 h 33 min_Exposure_50.0sec. multiple exposures-Figure2 -Aβ1-42-48h Administrator 2020-05-22 13 h 33 min_Exposure_205.2sec. multiple exposures-Figure2 -Aβ1-42-48h Administrator 2020-05-22 13 h 33 min_Exposure_291.4sec. multiple exposures-Figure2 -Aβ1-42-72h Administrator 2020-05-22 11 h 17 min_Exposure_58.6sec. multiple exposures-Figure2 -Aβ1-42-72h Administrator 2020-05-22 11 h 17 min_Exposure_231.0sec. multiple exposures-Figure2 -Aβ1-42-72h Administrator 2020-05-22 11 h 17 min_Exposure_300.0sec. multiple exposures-Figure2 -βactin-24h Administrator 2020-05-22 11 h 36 min_Exposure_50.0sec. multiple exposures-Figure2 -βactin-24h Administrator 2020-05-22 11 h 36 min_Exposure_239.6sec. multiple exposures-Figure2 -βactin-24h Administrator 2020-05-22 11 h 36 min_Exposure_300.0sec. multiple exposures-Figure2 -βactin-48h Administrator 2020-05-22 10 h 58 min_Exposure_50.0sec. multiple exposures-Figure2 -βactin-48h Administrator 2020-05-22 10 h 58 min_Exposure_282.7sec. multiple exposures-Figure2 -βactin-48h Administrator 2020-05-22 11 h 58 min_Exposure_300.0sec. multiple exposures-Figure2 -βactin-72h Administrator 2020-05-22 11 h 50 min_Exposure_50.0sec. multiple exposures-Figure2 -βactin-72h Administrator 2020-05-22 11 h 50 min_Exposure_205.2sec. multiple exposures-Figure2 -βactin-72h Administrator 2020-05-22 11 h 50 min_Exposure_300.0sec. [file 12868_2021_658_MOESM13_ESM.zip › multiple exposures/multiple exposures-Figure2 -a┬actin-24h Administrator 2020-05-22 11 ╩▒ 36 ╖╓_Exposure_300.0sec.pdf]

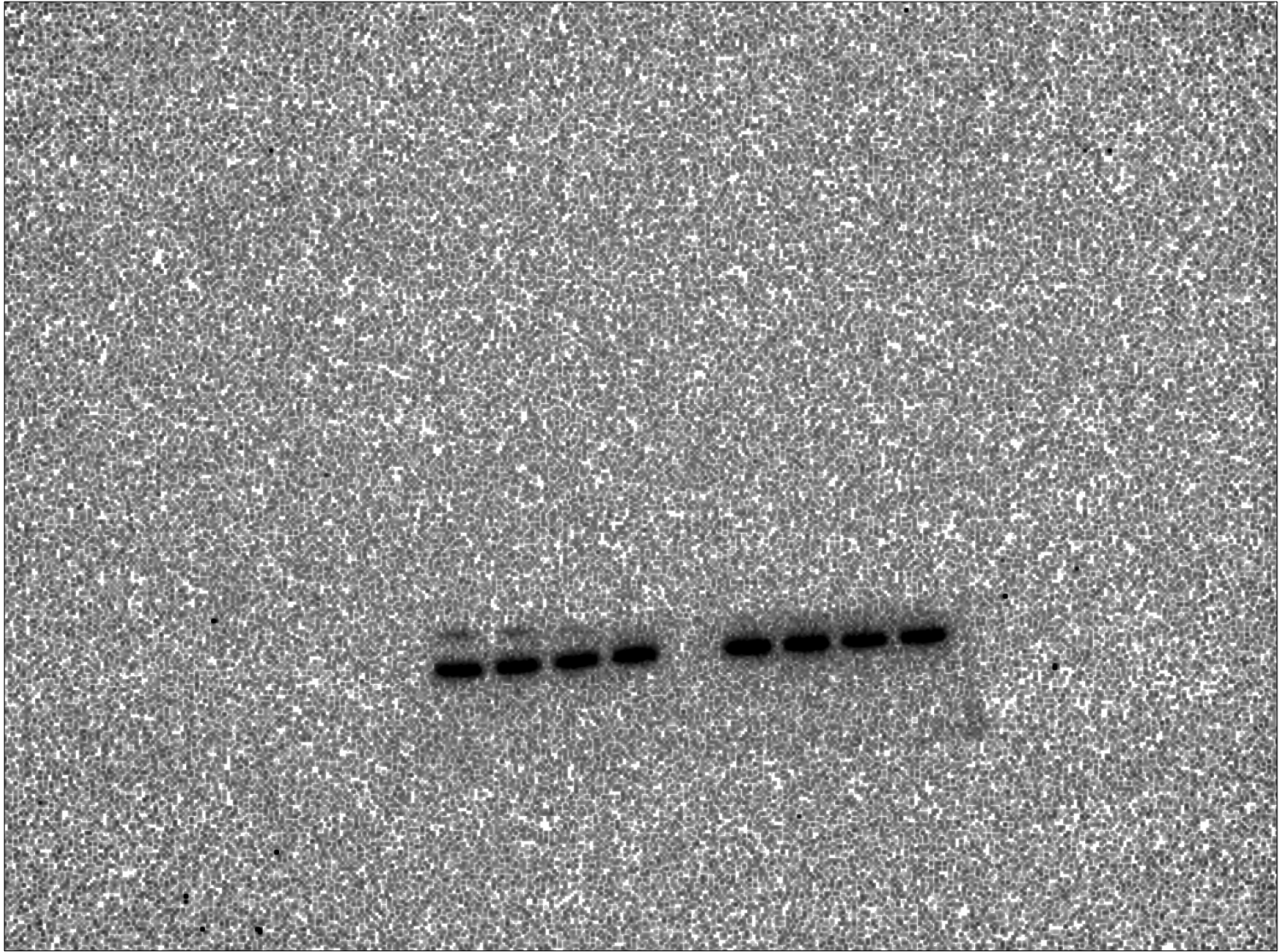

Supplement: Supplementary file 13 — Additional file 13: multiple exposures-Figure2 -Aβ1-42-24h Administrator 2020-05-22 13 h 48 min_Exposure_50.0sec. multiple exposures-Figure2 -Aβ1-42-24h Administrator 2020-05-22 13 h 48 min_Exposure_75.9sec. multiple exposures-Figure2 -Aβ1-42-24h Administrator 2020-05-22 13 h 48 min_Exposure_300.0sec. multiple exposures-Figure2 -Aβ1-42-48h Administrator 2020-05-22 13 h 33 min_Exposure_50.0sec. multiple exposures-Figure2 -Aβ1-42-48h Administrator 2020-05-22 13 h 33 min_Exposure_205.2sec. multiple exposures-Figure2 -Aβ1-42-48h Administrator 2020-05-22 13 h 33 min_Exposure_291.4sec. multiple exposures-Figure2 -Aβ1-42-72h Administrator 2020-05-22 11 h 17 min_Exposure_58.6sec. multiple exposures-Figure2 -Aβ1-42-72h Administrator 2020-05-22 11 h 17 min_Exposure_231.0sec. multiple exposures-Figure2 -Aβ1-42-72h Administrator 2020-05-22 11 h 17 min_Exposure_300.0sec. multiple exposures-Figure2 -βactin-24h Administrator 2020-05-22 11 h 36 min_Exposure_50.0sec. multiple exposures-Figure2 -βactin-24h Administrator 2020-05-22 11 h 36 min_Exposure_239.6sec. multiple exposures-Figure2 -βactin-24h Administrator 2020-05-22 11 h 36 min_Exposure_300.0sec. multiple exposures-Figure2 -βactin-48h Administrator 2020-05-22 10 h 58 min_Exposure_50.0sec. multiple exposures-Figure2 -βactin-48h Administrator 2020-05-22 10 h 58 min_Exposure_282.7sec. multiple exposures-Figure2 -βactin-48h Administrator 2020-05-22 11 h 58 min_Exposure_300.0sec. multiple exposures-Figure2 -βactin-72h Administrator 2020-05-22 11 h 50 min_Exposure_50.0sec. multiple exposures-Figure2 -βactin-72h Administrator 2020-05-22 11 h 50 min_Exposure_205.2sec. multiple exposures-Figure2 -βactin-72h Administrator 2020-05-22 11 h 50 min_Exposure_300.0sec. [file 12868_2021_658_MOESM13_ESM.zip › multiple exposures/multiple exposures-Figure2 -a┬actin-24h Administrator 2020-05-22 11 ╩▒ 36 ╖╓_Exposure_50.0sec.pdf]

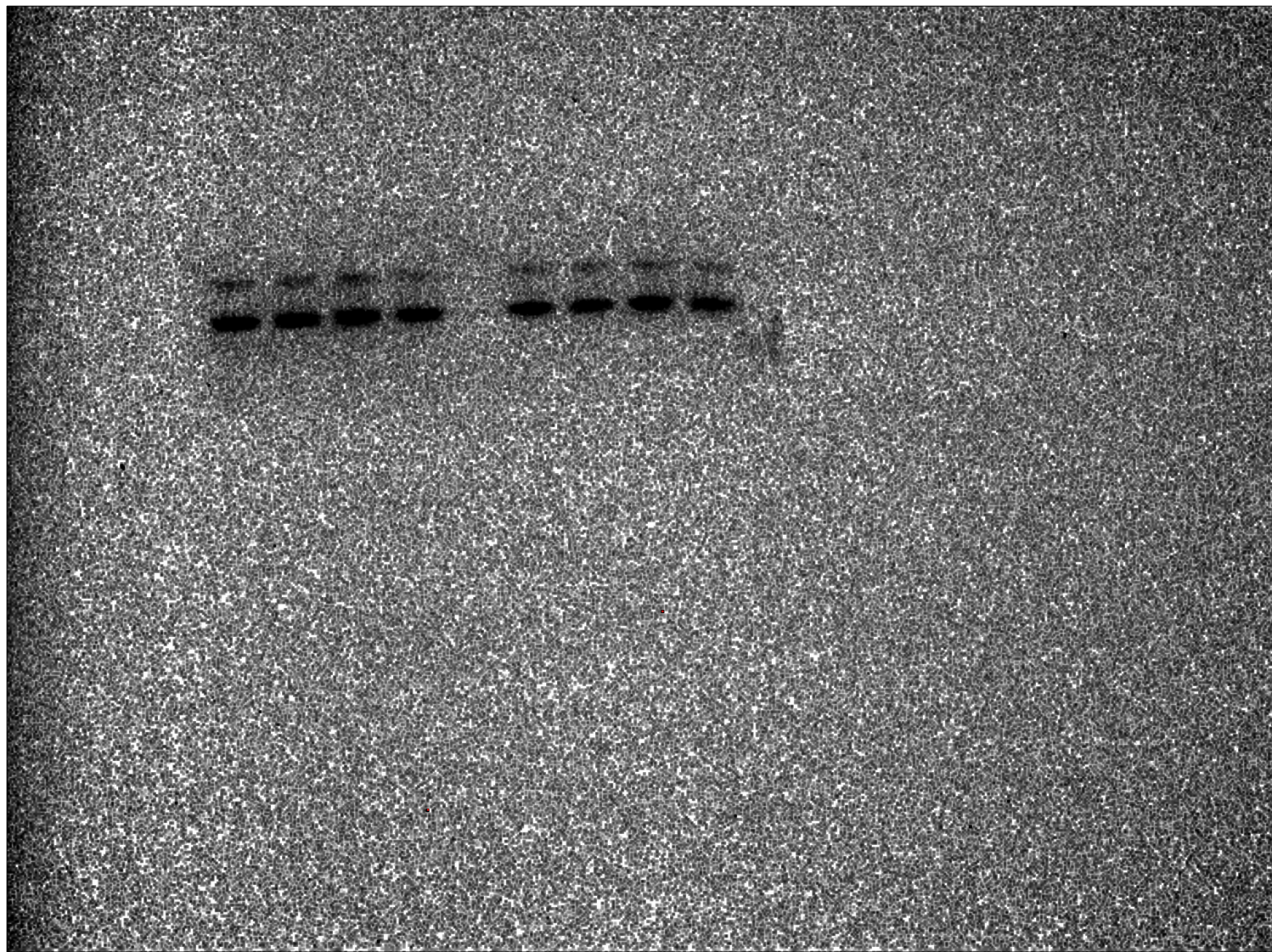

Supplement: Supplementary file 13 — Additional file 13: multiple exposures-Figure2 -Aβ1-42-24h Administrator 2020-05-22 13 h 48 min_Exposure_50.0sec. multiple exposures-Figure2 -Aβ1-42-24h Administrator 2020-05-22 13 h 48 min_Exposure_75.9sec. multiple exposures-Figure2 -Aβ1-42-24h Administrator 2020-05-22 13 h 48 min_Exposure_300.0sec. multiple exposures-Figure2 -Aβ1-42-48h Administrator 2020-05-22 13 h 33 min_Exposure_50.0sec. multiple exposures-Figure2 -Aβ1-42-48h Administrator 2020-05-22 13 h 33 min_Exposure_205.2sec. multiple exposures-Figure2 -Aβ1-42-48h Administrator 2020-05-22 13 h 33 min_Exposure_291.4sec. multiple exposures-Figure2 -Aβ1-42-72h Administrator 2020-05-22 11 h 17 min_Exposure_58.6sec. multiple exposures-Figure2 -Aβ1-42-72h Administrator 2020-05-22 11 h 17 min_Exposure_231.0sec. multiple exposures-Figure2 -Aβ1-42-72h Administrator 2020-05-22 11 h 17 min_Exposure_300.0sec. multiple exposures-Figure2 -βactin-24h Administrator 2020-05-22 11 h 36 min_Exposure_50.0sec. multiple exposures-Figure2 -βactin-24h Administrator 2020-05-22 11 h 36 min_Exposure_239.6sec. multiple exposures-Figure2 -βactin-24h Administrator 2020-05-22 11 h 36 min_Exposure_300.0sec. multiple exposures-Figure2 -βactin-48h Administrator 2020-05-22 10 h 58 min_Exposure_50.0sec. multiple exposures-Figure2 -βactin-48h Administrator 2020-05-22 10 h 58 min_Exposure_282.7sec. multiple exposures-Figure2 -βactin-48h Administrator 2020-05-22 11 h 58 min_Exposure_300.0sec. multiple exposures-Figure2 -βactin-72h Administrator 2020-05-22 11 h 50 min_Exposure_50.0sec. multiple exposures-Figure2 -βactin-72h Administrator 2020-05-22 11 h 50 min_Exposure_205.2sec. multiple exposures-Figure2 -βactin-72h Administrator 2020-05-22 11 h 50 min_Exposure_300.0sec. [file 12868_2021_658_MOESM13_ESM.zip › multiple exposures/multiple exposures-Figure2 -a┬actin-48h Administrator 2020-05-22 10 ╩▒ 58 ╖╓_Exposure_282.7sec.pdf]

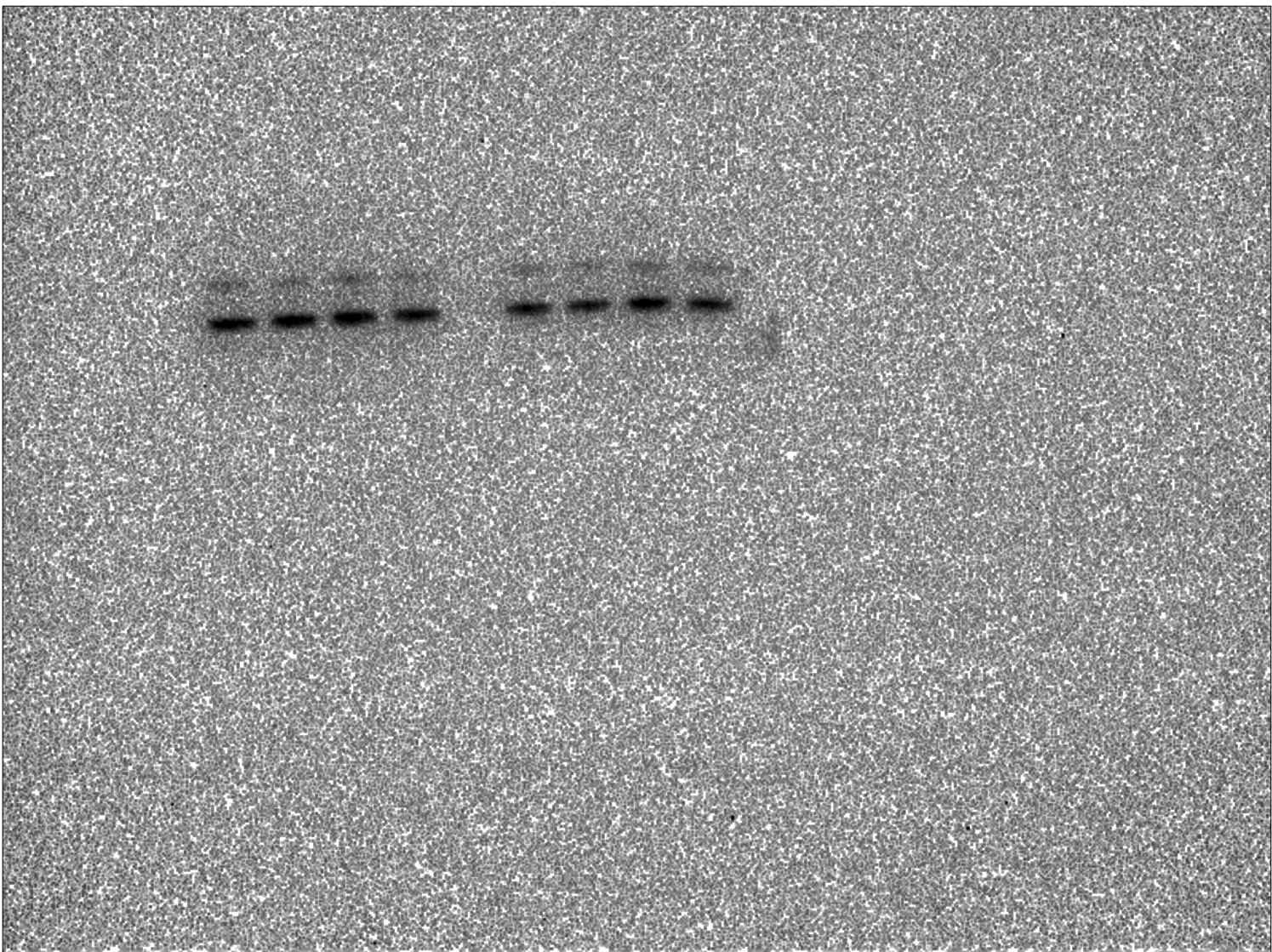

Supplement: Supplementary file 13 — Additional file 13: multiple exposures-Figure2 -Aβ1-42-24h Administrator 2020-05-22 13 h 48 min_Exposure_50.0sec. multiple exposures-Figure2 -Aβ1-42-24h Administrator 2020-05-22 13 h 48 min_Exposure_75.9sec. multiple exposures-Figure2 -Aβ1-42-24h Administrator 2020-05-22 13 h 48 min_Exposure_300.0sec. multiple exposures-Figure2 -Aβ1-42-48h Administrator 2020-05-22 13 h 33 min_Exposure_50.0sec. multiple exposures-Figure2 -Aβ1-42-48h Administrator 2020-05-22 13 h 33 min_Exposure_205.2sec. multiple exposures-Figure2 -Aβ1-42-48h Administrator 2020-05-22 13 h 33 min_Exposure_291.4sec. multiple exposures-Figure2 -Aβ1-42-72h Administrator 2020-05-22 11 h 17 min_Exposure_58.6sec. multiple exposures-Figure2 -Aβ1-42-72h Administrator 2020-05-22 11 h 17 min_Exposure_231.0sec. multiple exposures-Figure2 -Aβ1-42-72h Administrator 2020-05-22 11 h 17 min_Exposure_300.0sec. multiple exposures-Figure2 -βactin-24h Administrator 2020-05-22 11 h 36 min_Exposure_50.0sec. multiple exposures-Figure2 -βactin-24h Administrator 2020-05-22 11 h 36 min_Exposure_239.6sec. multiple exposures-Figure2 -βactin-24h Administrator 2020-05-22 11 h 36 min_Exposure_300.0sec. multiple exposures-Figure2 -βactin-48h Administrator 2020-05-22 10 h 58 min_Exposure_50.0sec. multiple exposures-Figure2 -βactin-48h Administrator 2020-05-22 10 h 58 min_Exposure_282.7sec. multiple exposures-Figure2 -βactin-48h Administrator 2020-05-22 11 h 58 min_Exposure_300.0sec. multiple exposures-Figure2 -βactin-72h Administrator 2020-05-22 11 h 50 min_Exposure_50.0sec. multiple exposures-Figure2 -βactin-72h Administrator 2020-05-22 11 h 50 min_Exposure_205.2sec. multiple exposures-Figure2 -βactin-72h Administrator 2020-05-22 11 h 50 min_Exposure_300.0sec. [file 12868_2021_658_MOESM13_ESM.zip › multiple exposures/multiple exposures-Figure2 -a┬actin-48h Administrator 2020-05-22 10 ╩▒ 58 ╖╓_Exposure_50.0sec.pdf]

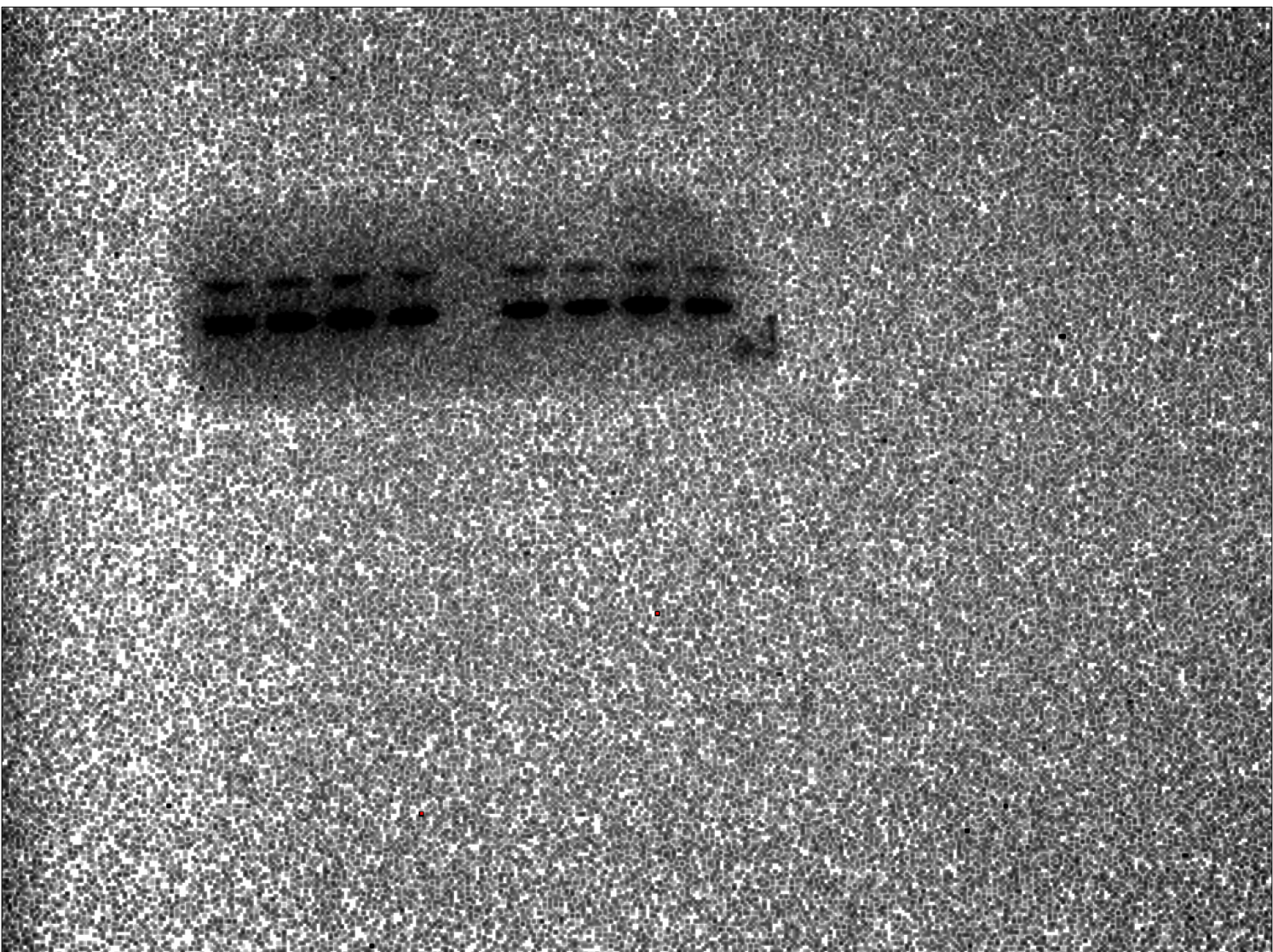

Supplement: Supplementary file 13 — Additional file 13: multiple exposures-Figure2 -Aβ1-42-24h Administrator 2020-05-22 13 h 48 min_Exposure_50.0sec. multiple exposures-Figure2 -Aβ1-42-24h Administrator 2020-05-22 13 h 48 min_Exposure_75.9sec. multiple exposures-Figure2 -Aβ1-42-24h Administrator 2020-05-22 13 h 48 min_Exposure_300.0sec. multiple exposures-Figure2 -Aβ1-42-48h Administrator 2020-05-22 13 h 33 min_Exposure_50.0sec. multiple exposures-Figure2 -Aβ1-42-48h Administrator 2020-05-22 13 h 33 min_Exposure_205.2sec. multiple exposures-Figure2 -Aβ1-42-48h Administrator 2020-05-22 13 h 33 min_Exposure_291.4sec. multiple exposures-Figure2 -Aβ1-42-72h Administrator 2020-05-22 11 h 17 min_Exposure_58.6sec. multiple exposures-Figure2 -Aβ1-42-72h Administrator 2020-05-22 11 h 17 min_Exposure_231.0sec. multiple exposures-Figure2 -Aβ1-42-72h Administrator 2020-05-22 11 h 17 min_Exposure_300.0sec. multiple exposures-Figure2 -βactin-24h Administrator 2020-05-22 11 h 36 min_Exposure_50.0sec. multiple exposures-Figure2 -βactin-24h Administrator 2020-05-22 11 h 36 min_Exposure_239.6sec. multiple exposures-Figure2 -βactin-24h Administrator 2020-05-22 11 h 36 min_Exposure_300.0sec. multiple exposures-Figure2 -βactin-48h Administrator 2020-05-22 10 h 58 min_Exposure_50.0sec. multiple exposures-Figure2 -βactin-48h Administrator 2020-05-22 10 h 58 min_Exposure_282.7sec. multiple exposures-Figure2 -βactin-48h Administrator 2020-05-22 11 h 58 min_Exposure_300.0sec. multiple exposures-Figure2 -βactin-72h Administrator 2020-05-22 11 h 50 min_Exposure_50.0sec. multiple exposures-Figure2 -βactin-72h Administrator 2020-05-22 11 h 50 min_Exposure_205.2sec. multiple exposures-Figure2 -βactin-72h Administrator 2020-05-22 11 h 50 min_Exposure_300.0sec. [file 12868_2021_658_MOESM13_ESM.zip › multiple exposures/multiple exposures-Figure2 -a┬actin-48h Administrator 2020-05-22 11 ╩▒ 04 ╖╓_Exposure_300.0sec.pdf]

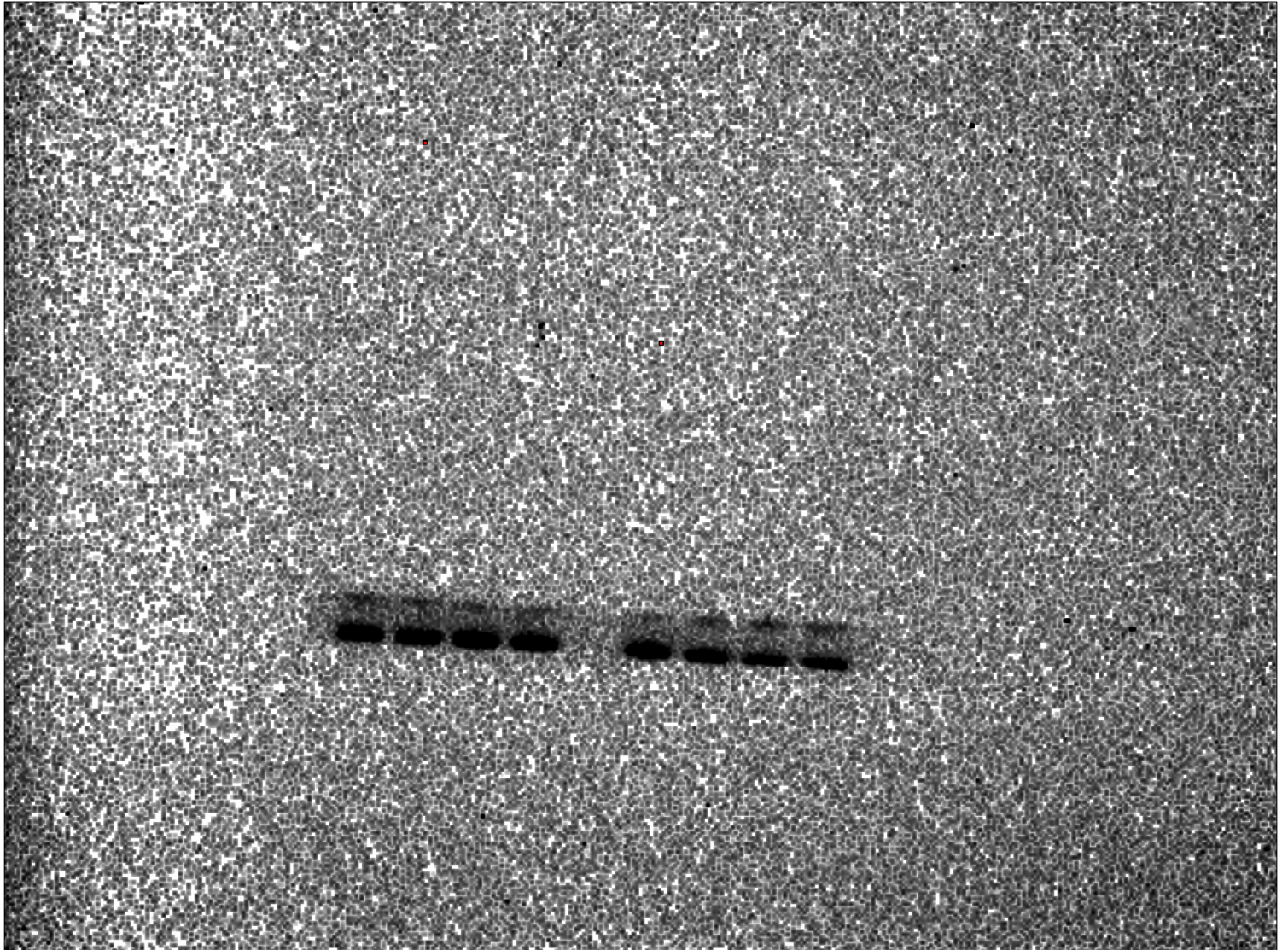

Supplement: Supplementary file 13 — Additional file 13: multiple exposures-Figure2 -Aβ1-42-24h Administrator 2020-05-22 13 h 48 min_Exposure_50.0sec. multiple exposures-Figure2 -Aβ1-42-24h Administrator 2020-05-22 13 h 48 min_Exposure_75.9sec. multiple exposures-Figure2 -Aβ1-42-24h Administrator 2020-05-22 13 h 48 min_Exposure_300.0sec. multiple exposures-Figure2 -Aβ1-42-48h Administrator 2020-05-22 13 h 33 min_Exposure_50.0sec. multiple exposures-Figure2 -Aβ1-42-48h Administrator 2020-05-22 13 h 33 min_Exposure_205.2sec. multiple exposures-Figure2 -Aβ1-42-48h Administrator 2020-05-22 13 h 33 min_Exposure_291.4sec. multiple exposures-Figure2 -Aβ1-42-72h Administrator 2020-05-22 11 h 17 min_Exposure_58.6sec. multiple exposures-Figure2 -Aβ1-42-72h Administrator 2020-05-22 11 h 17 min_Exposure_231.0sec. multiple exposures-Figure2 -Aβ1-42-72h Administrator 2020-05-22 11 h 17 min_Exposure_300.0sec. multiple exposures-Figure2 -βactin-24h Administrator 2020-05-22 11 h 36 min_Exposure_50.0sec. multiple exposures-Figure2 -βactin-24h Administrator 2020-05-22 11 h 36 min_Exposure_239.6sec. multiple exposures-Figure2 -βactin-24h Administrator 2020-05-22 11 h 36 min_Exposure_300.0sec. multiple exposures-Figure2 -βactin-48h Administrator 2020-05-22 10 h 58 min_Exposure_50.0sec. multiple exposures-Figure2 -βactin-48h Administrator 2020-05-22 10 h 58 min_Exposure_282.7sec. multiple exposures-Figure2 -βactin-48h Administrator 2020-05-22 11 h 58 min_Exposure_300.0sec. multiple exposures-Figure2 -βactin-72h Administrator 2020-05-22 11 h 50 min_Exposure_50.0sec. multiple exposures-Figure2 -βactin-72h Administrator 2020-05-22 11 h 50 min_Exposure_205.2sec. multiple exposures-Figure2 -βactin-72h Administrator 2020-05-22 11 h 50 min_Exposure_300.0sec. [file 12868_2021_658_MOESM13_ESM.zip › multiple exposures/multiple exposures-Figure2 -a┬actin-72h Administrator 2020-05-22 11 ╩▒ 50 ╖╓_Exposure_205.2sec.pdf]

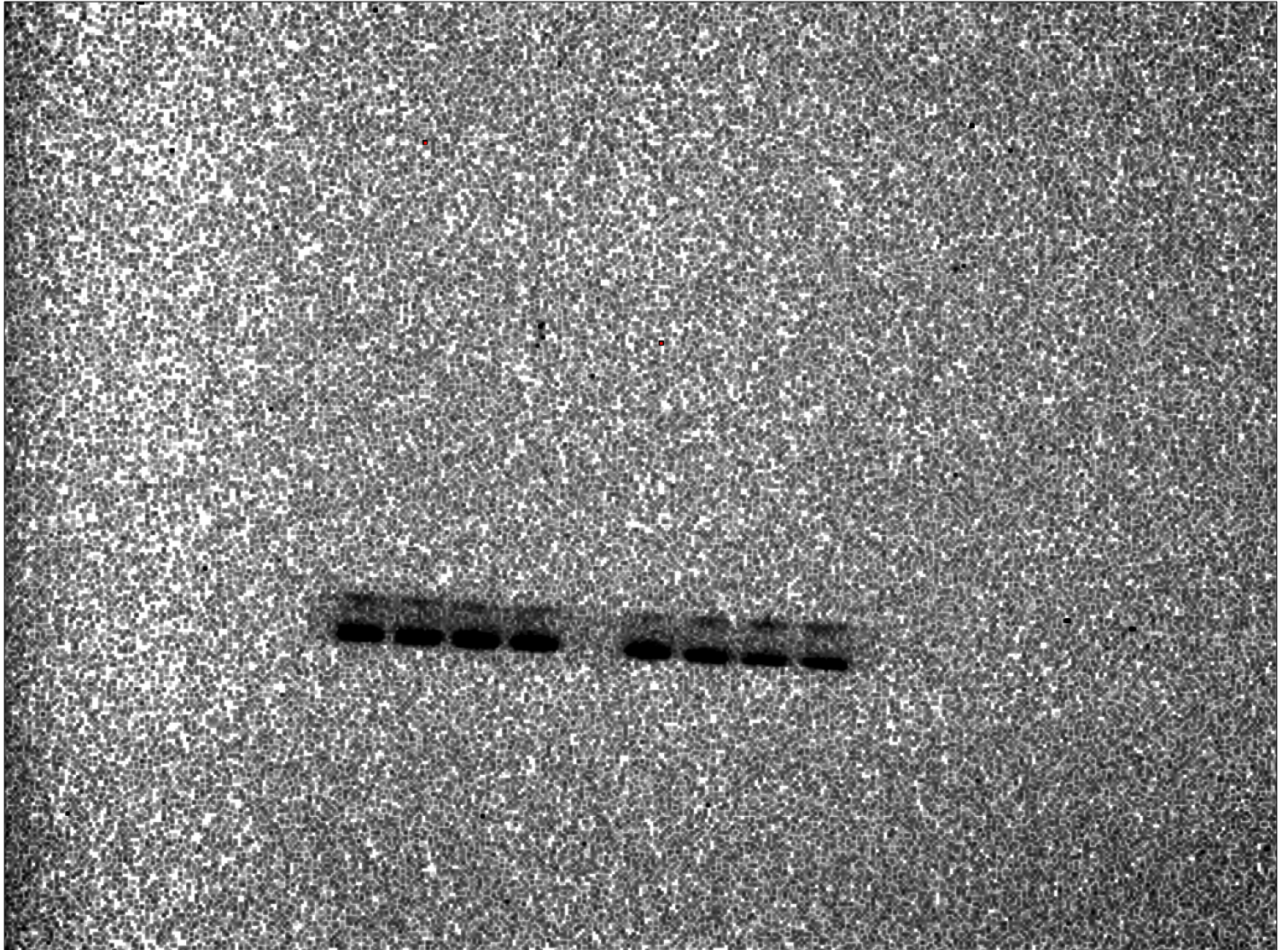

Supplement: Supplementary file 13 — Additional file 13: multiple exposures-Figure2 -Aβ1-42-24h Administrator 2020-05-22 13 h 48 min_Exposure_50.0sec. multiple exposures-Figure2 -Aβ1-42-24h Administrator 2020-05-22 13 h 48 min_Exposure_75.9sec. multiple exposures-Figure2 -Aβ1-42-24h Administrator 2020-05-22 13 h 48 min_Exposure_300.0sec. multiple exposures-Figure2 -Aβ1-42-48h Administrator 2020-05-22 13 h 33 min_Exposure_50.0sec. multiple exposures-Figure2 -Aβ1-42-48h Administrator 2020-05-22 13 h 33 min_Exposure_205.2sec. multiple exposures-Figure2 -Aβ1-42-48h Administrator 2020-05-22 13 h 33 min_Exposure_291.4sec. multiple exposures-Figure2 -Aβ1-42-72h Administrator 2020-05-22 11 h 17 min_Exposure_58.6sec. multiple exposures-Figure2 -Aβ1-42-72h Administrator 2020-05-22 11 h 17 min_Exposure_231.0sec. multiple exposures-Figure2 -Aβ1-42-72h Administrator 2020-05-22 11 h 17 min_Exposure_300.0sec. multiple exposures-Figure2 -βactin-24h Administrator 2020-05-22 11 h 36 min_Exposure_50.0sec. multiple exposures-Figure2 -βactin-24h Administrator 2020-05-22 11 h 36 min_Exposure_239.6sec. multiple exposures-Figure2 -βactin-24h Administrator 2020-05-22 11 h 36 min_Exposure_300.0sec. multiple exposures-Figure2 -βactin-48h Administrator 2020-05-22 10 h 58 min_Exposure_50.0sec. multiple exposures-Figure2 -βactin-48h Administrator 2020-05-22 10 h 58 min_Exposure_282.7sec. multiple exposures-Figure2 -βactin-48h Administrator 2020-05-22 11 h 58 min_Exposure_300.0sec. multiple exposures-Figure2 -βactin-72h Administrator 2020-05-22 11 h 50 min_Exposure_50.0sec. multiple exposures-Figure2 -βactin-72h Administrator 2020-05-22 11 h 50 min_Exposure_205.2sec. multiple exposures-Figure2 -βactin-72h Administrator 2020-05-22 11 h 50 min_Exposure_300.0sec. [file 12868_2021_658_MOESM13_ESM.zip › multiple exposures/multiple exposures-Figure2 -a┬actin-72h Administrator 2020-05-22 11 ╩▒ 50 ╖╓_Exposure_300.0sec.pdf]

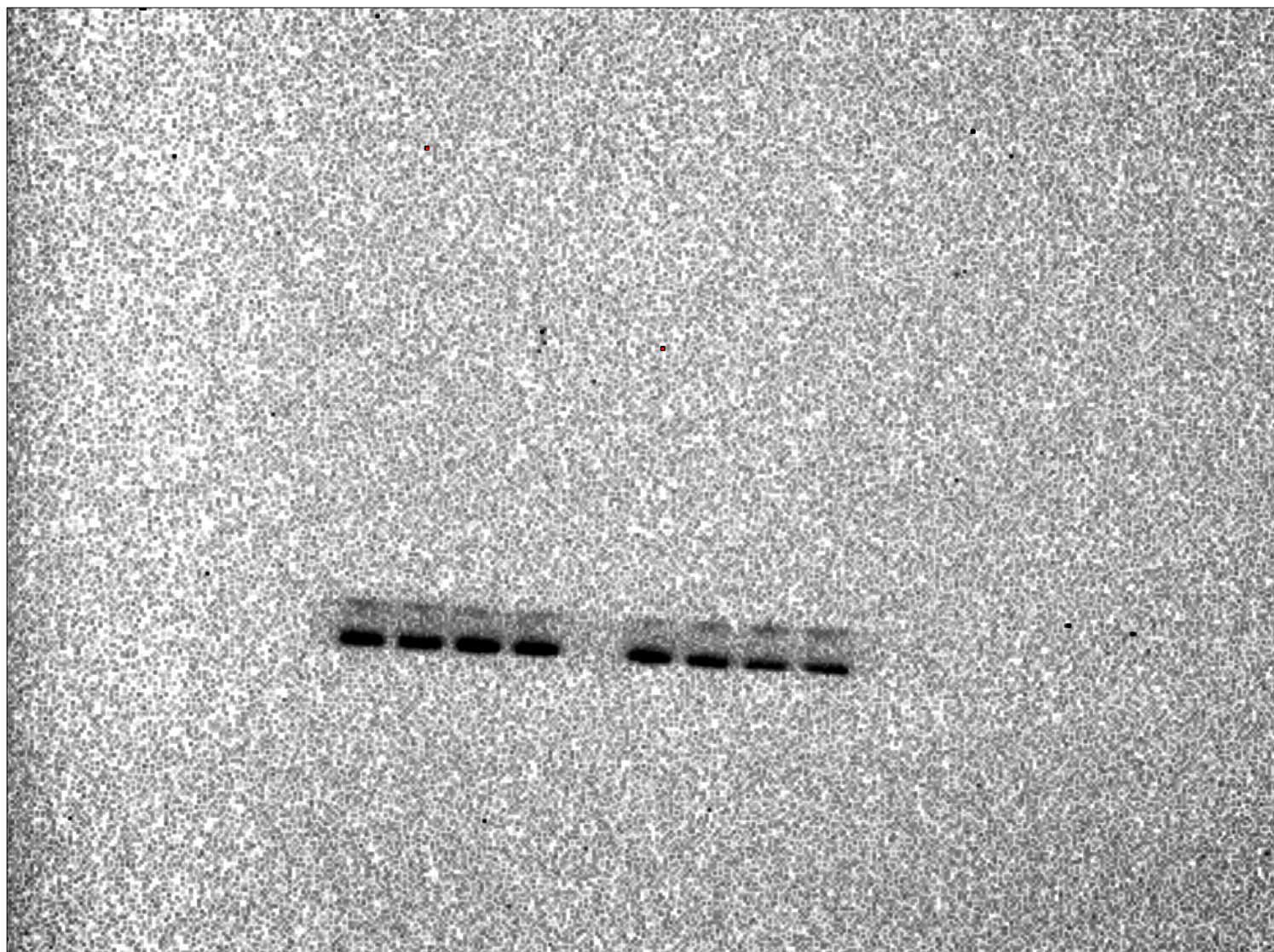

Supplement: Supplementary file 13 — Additional file 13: multiple exposures-Figure2 -Aβ1-42-24h Administrator 2020-05-22 13 h 48 min_Exposure_50.0sec. multiple exposures-Figure2 -Aβ1-42-24h Administrator 2020-05-22 13 h 48 min_Exposure_75.9sec. multiple exposures-Figure2 -Aβ1-42-24h Administrator 2020-05-22 13 h 48 min_Exposure_300.0sec. multiple exposures-Figure2 -Aβ1-42-48h Administrator 2020-05-22 13 h 33 min_Exposure_50.0sec. multiple exposures-Figure2 -Aβ1-42-48h Administrator 2020-05-22 13 h 33 min_Exposure_205.2sec. multiple exposures-Figure2 -Aβ1-42-48h Administrator 2020-05-22 13 h 33 min_Exposure_291.4sec. multiple exposures-Figure2 -Aβ1-42-72h Administrator 2020-05-22 11 h 17 min_Exposure_58.6sec. multiple exposures-Figure2 -Aβ1-42-72h Administrator 2020-05-22 11 h 17 min_Exposure_231.0sec. multiple exposures-Figure2 -Aβ1-42-72h Administrator 2020-05-22 11 h 17 min_Exposure_300.0sec. multiple exposures-Figure2 -βactin-24h Administrator 2020-05-22 11 h 36 min_Exposure_50.0sec. multiple exposures-Figure2 -βactin-24h Administrator 2020-05-22 11 h 36 min_Exposure_239.6sec. multiple exposures-Figure2 -βactin-24h Administrator 2020-05-22 11 h 36 min_Exposure_300.0sec. multiple exposures-Figure2 -βactin-48h Administrator 2020-05-22 10 h 58 min_Exposure_50.0sec. multiple exposures-Figure2 -βactin-48h Administrator 2020-05-22 10 h 58 min_Exposure_282.7sec. multiple exposures-Figure2 -βactin-48h Administrator 2020-05-22 11 h 58 min_Exposure_300.0sec. multiple exposures-Figure2 -βactin-72h Administrator 2020-05-22 11 h 50 min_Exposure_50.0sec. multiple exposures-Figure2 -βactin-72h Administrator 2020-05-22 11 h 50 min_Exposure_205.2sec. multiple exposures-Figure2 -βactin-72h Administrator 2020-05-22 11 h 50 min_Exposure_300.0sec. [file 12868_2021_658_MOESM13_ESM.zip › multiple exposures/multiple exposures-Figure2 -a┬actin-72h Administrator 2020-05-22 11 ╩▒ 50 ╖╓_Exposure_50.0sec.pdf]
