# Supplementary material for: Uptake of Aβ by OATPs might be a new pathophysiological mechanism of Alzheimer disease
Source: BMC Neurosci. 2021 Sep 14;22:53. doi: 10.1186/s12868-021-00658-9 (PMC8439072; doi:10.1186/s12868-021-00658-9)

## Slide 1
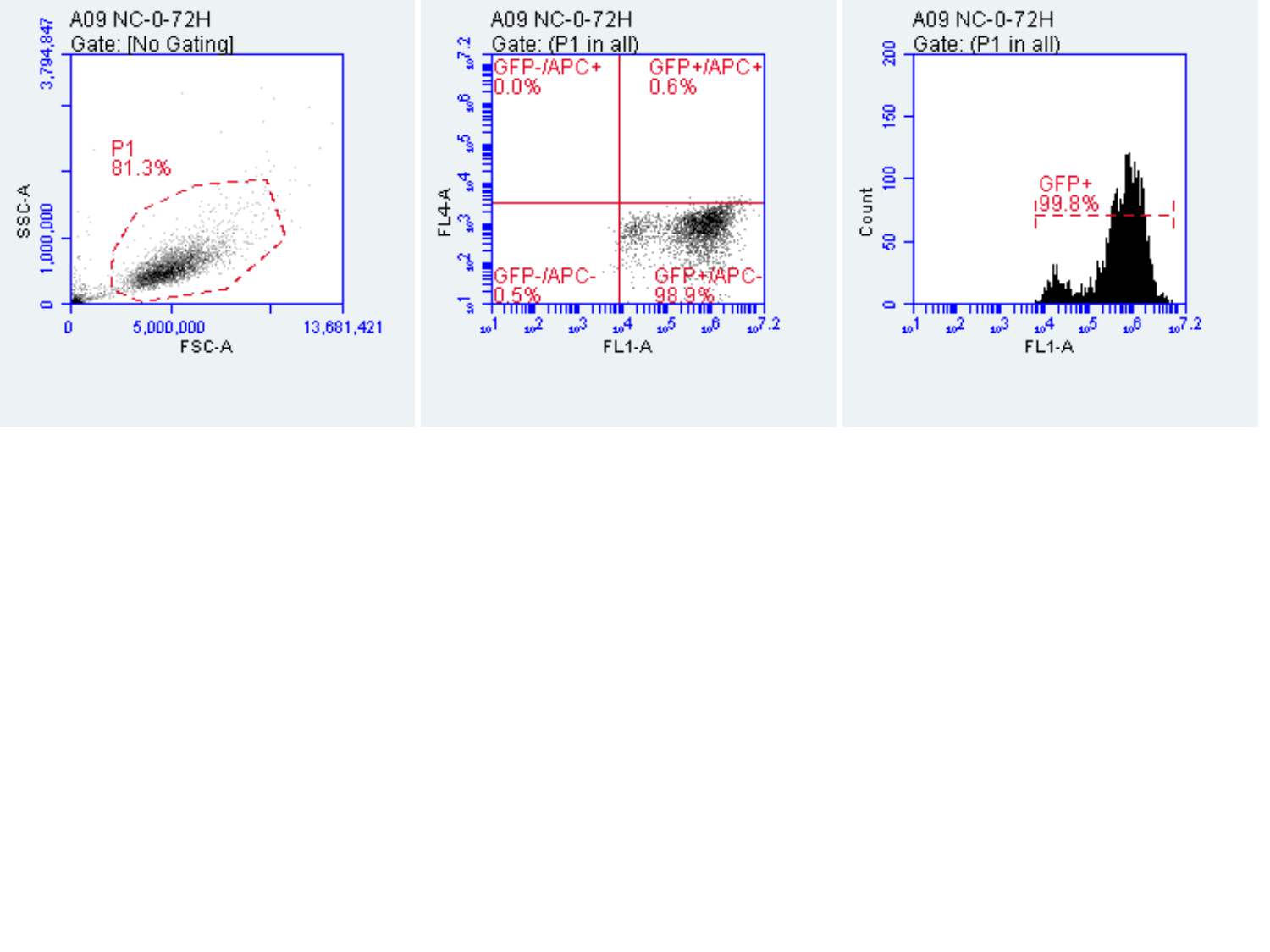

## Slide 2
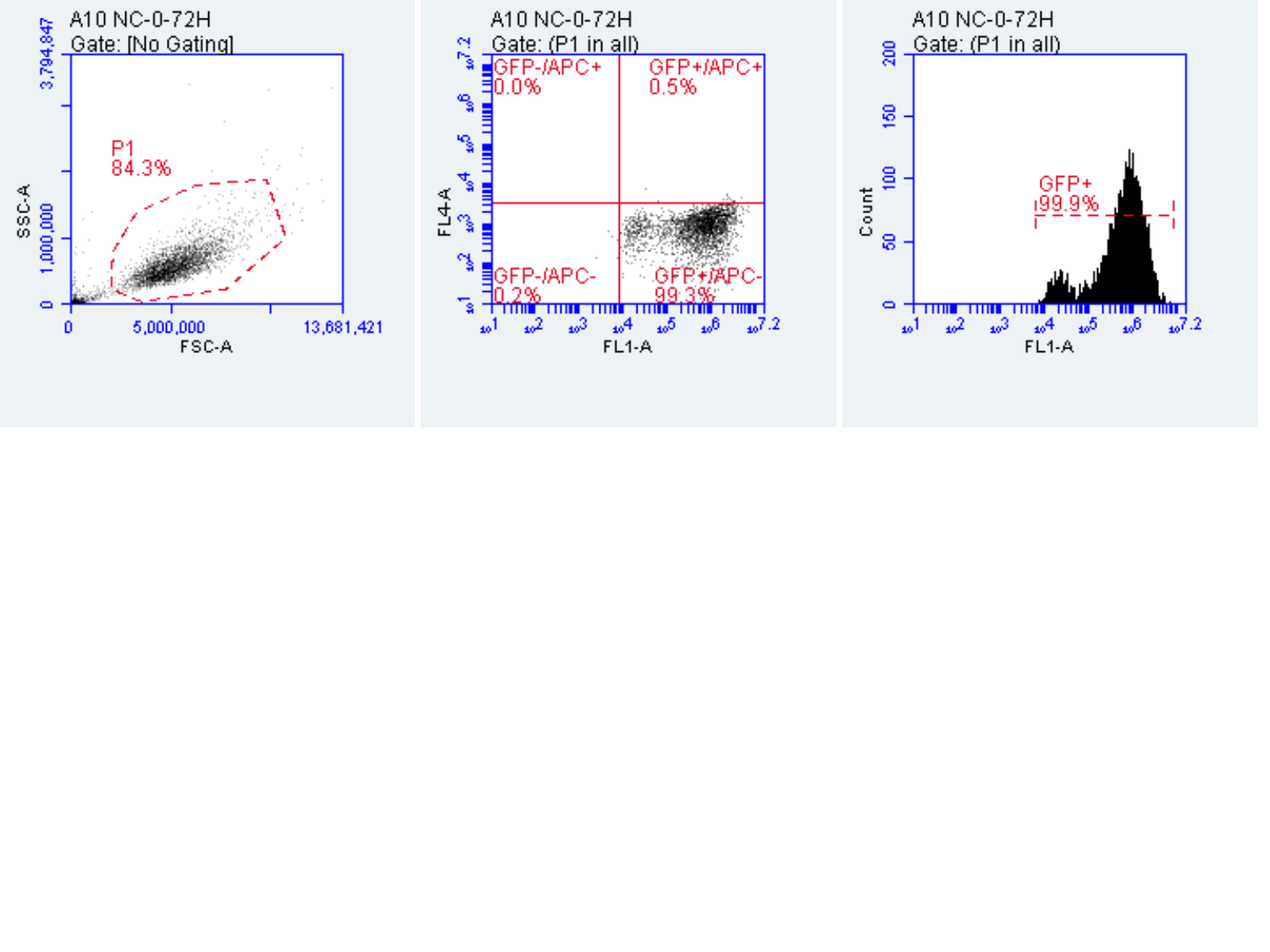

## Slide 3
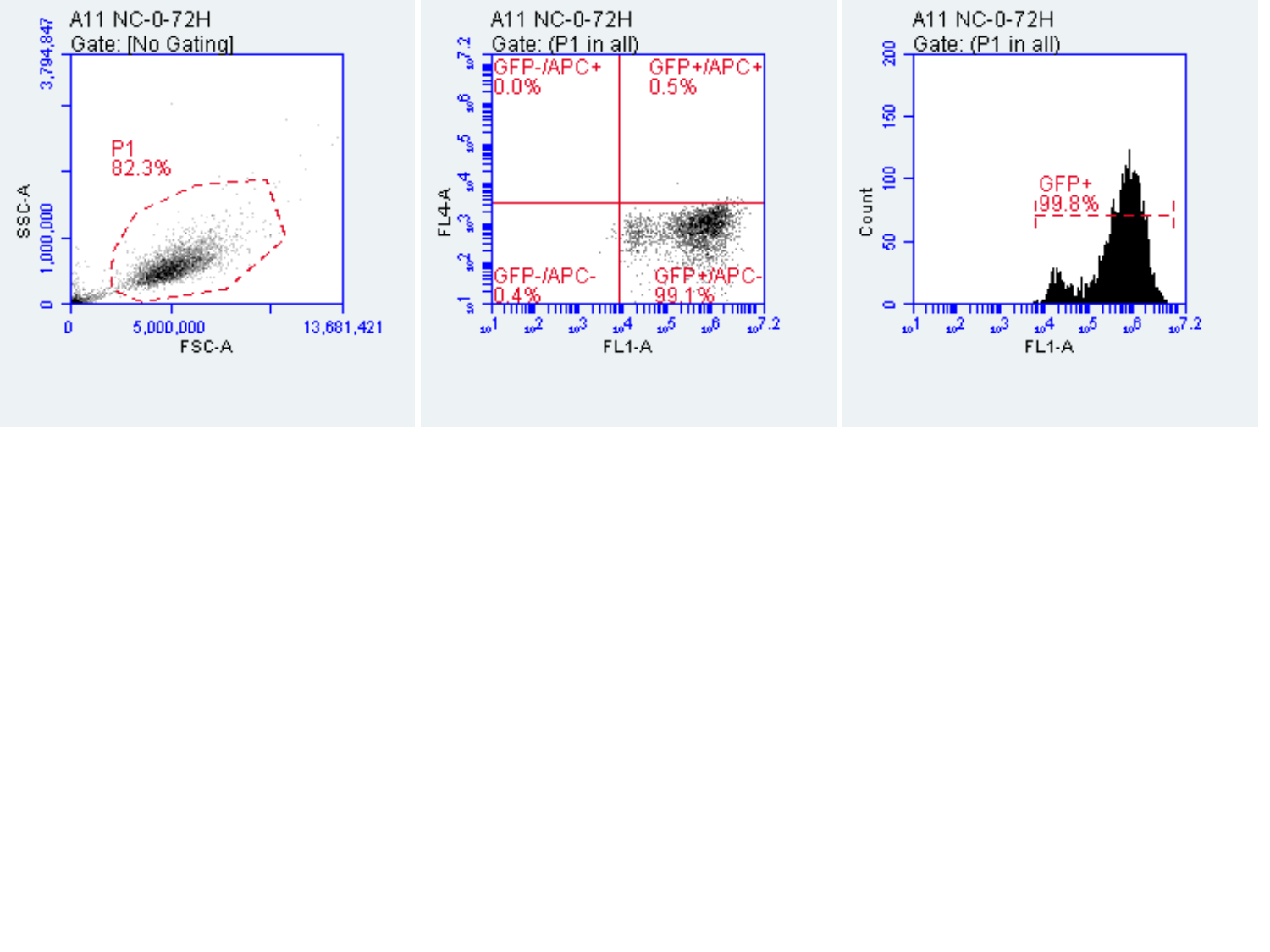

## Slide 4
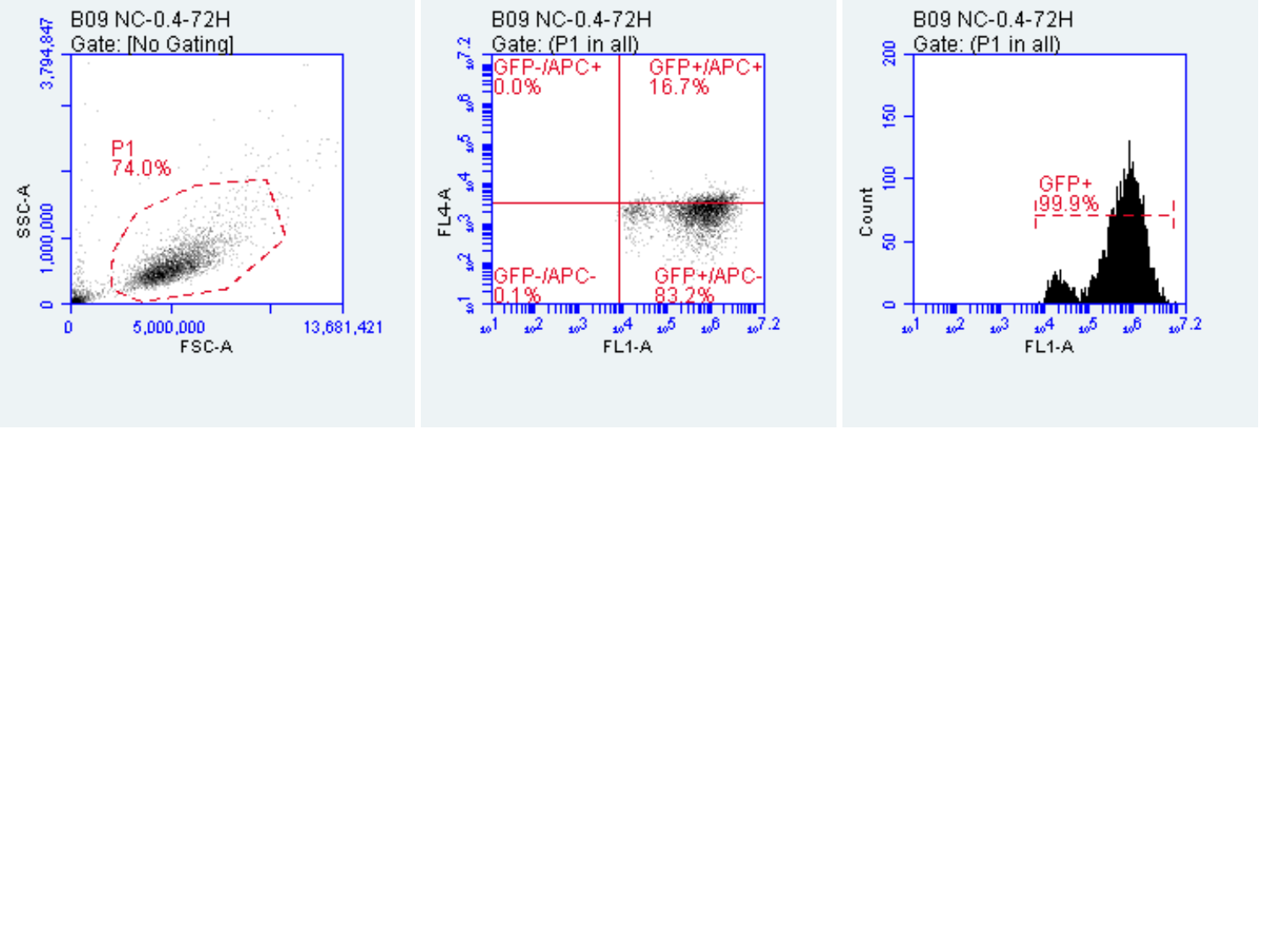

## Slide 5
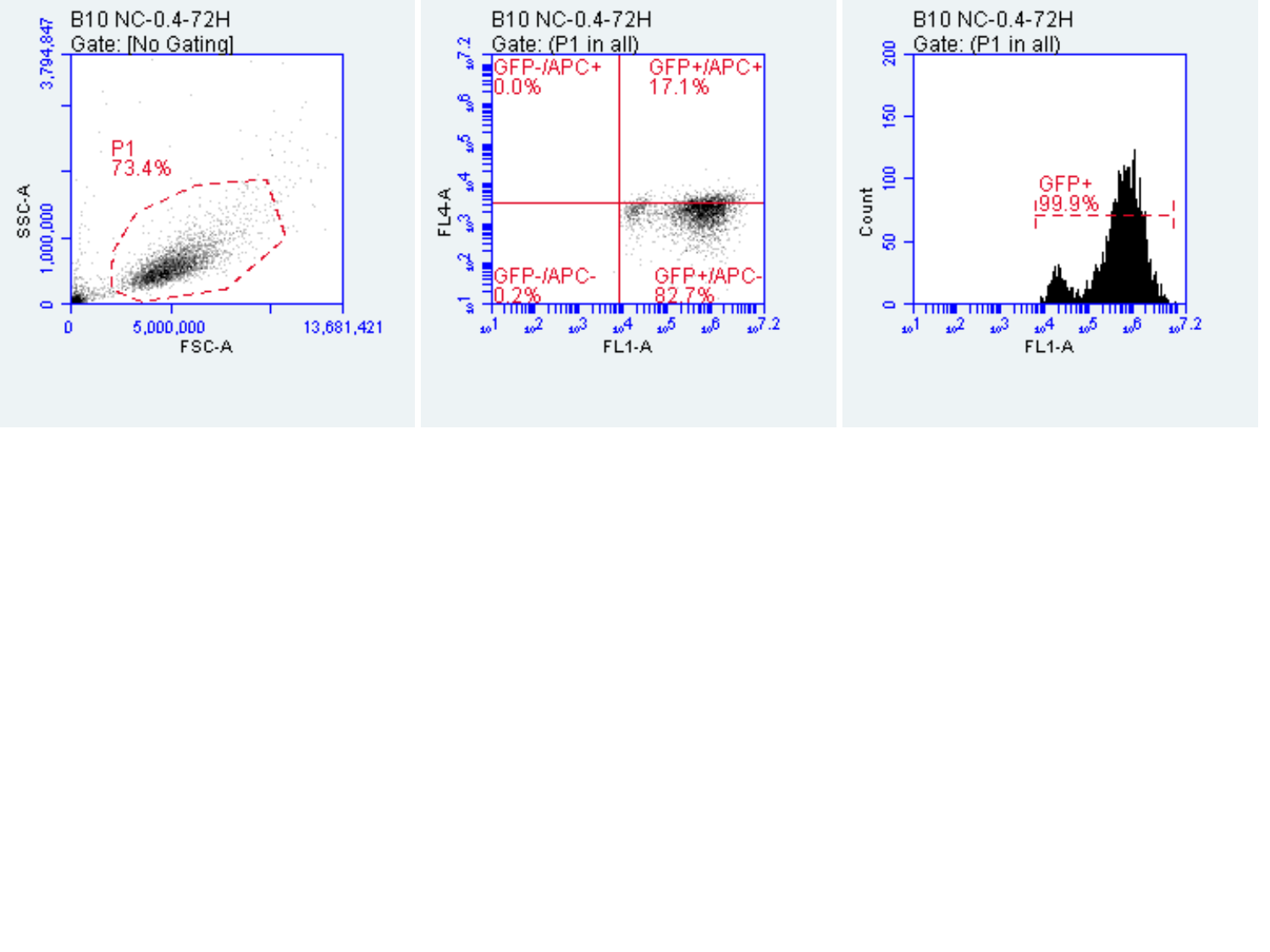

## Slide 6
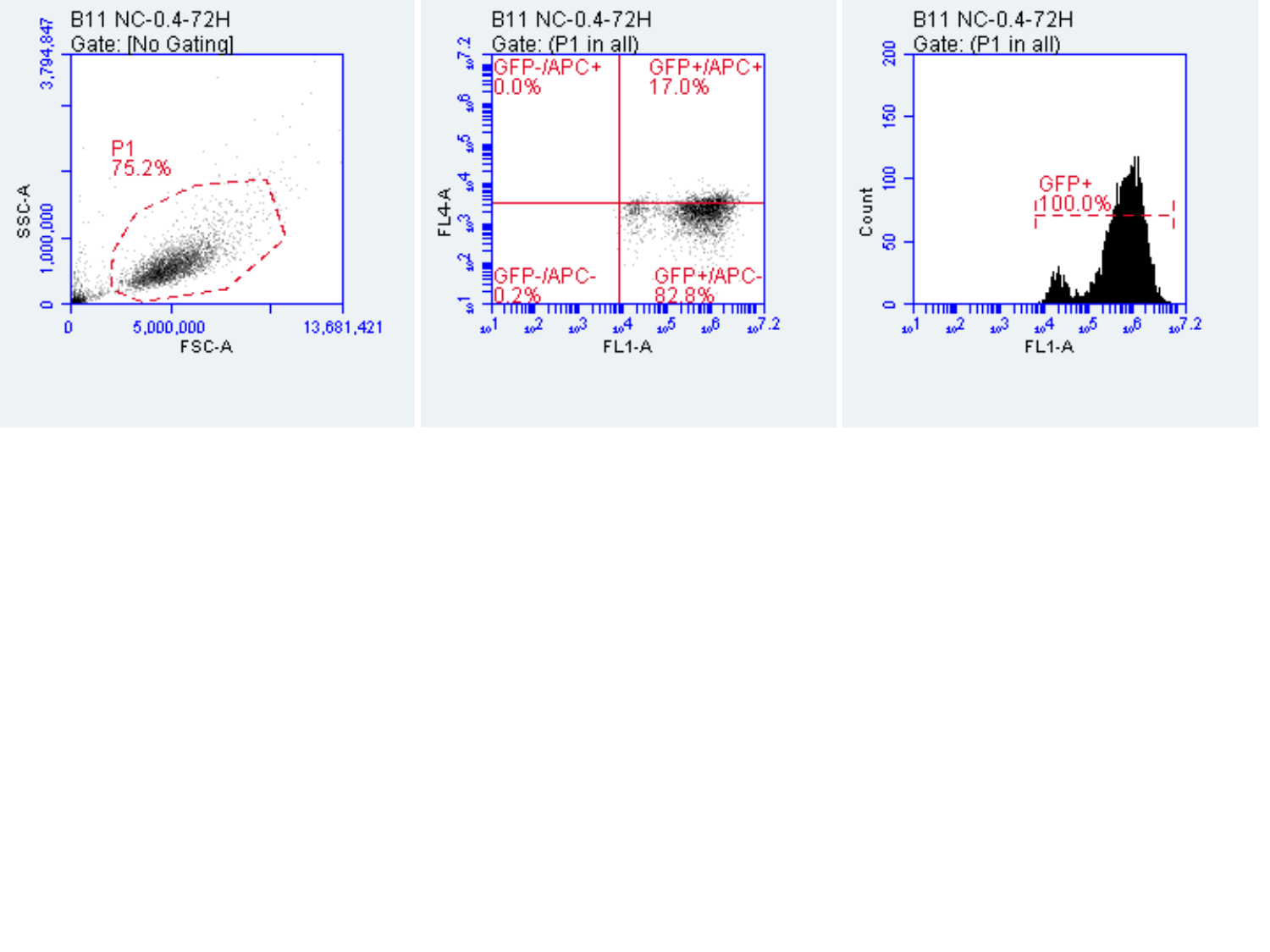

## Slide 7
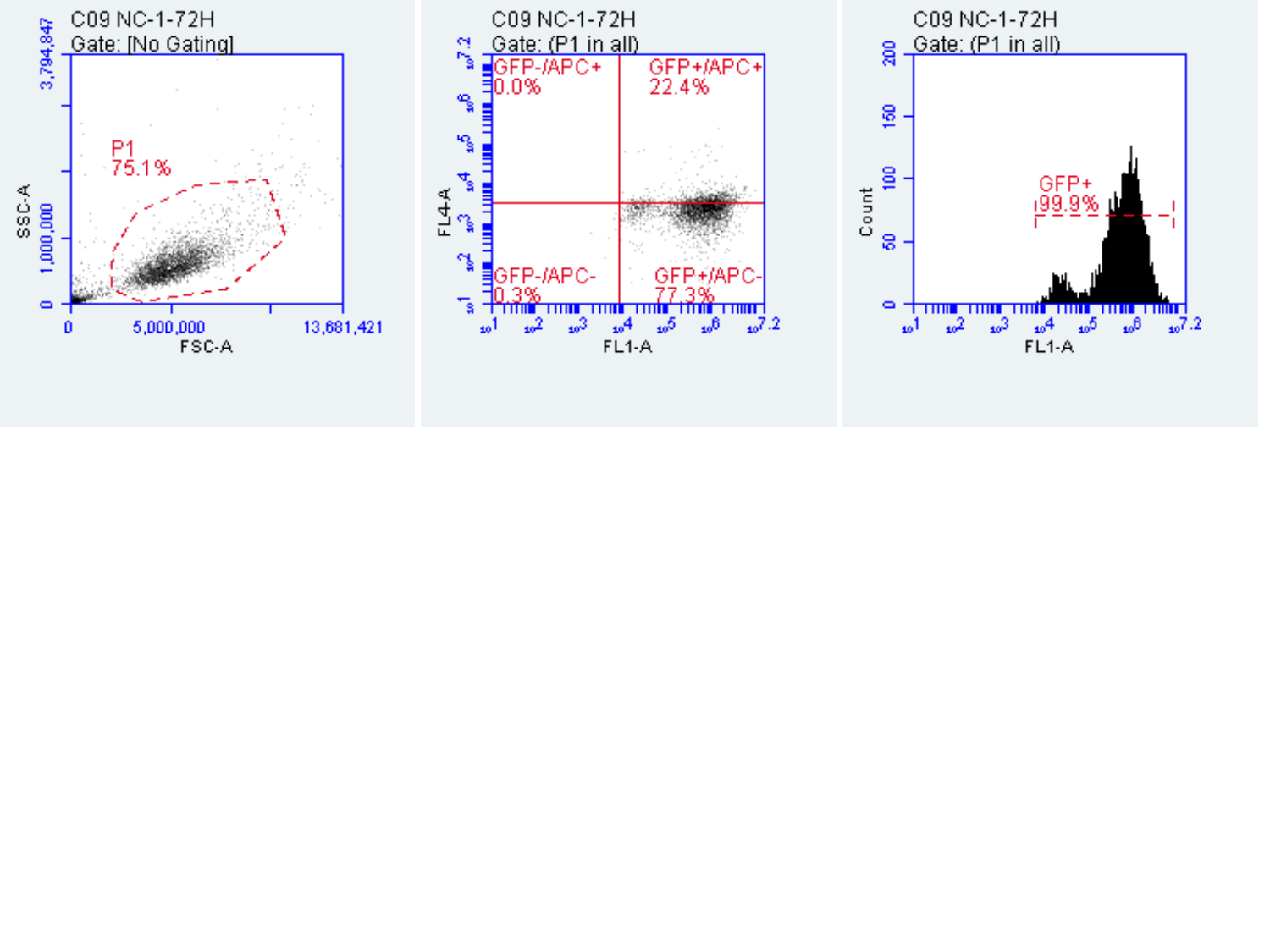

## Slide 8
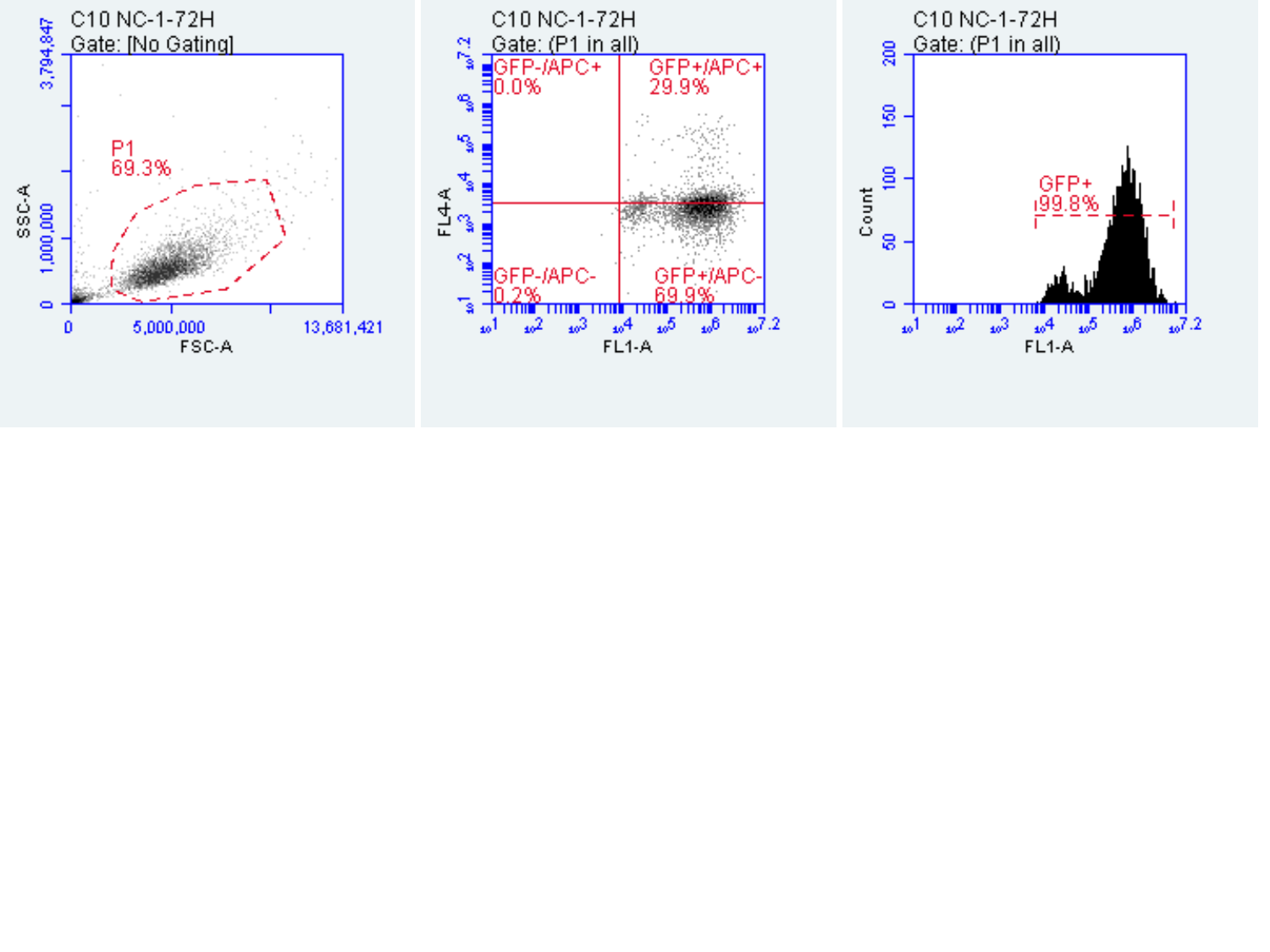

## Slide 9
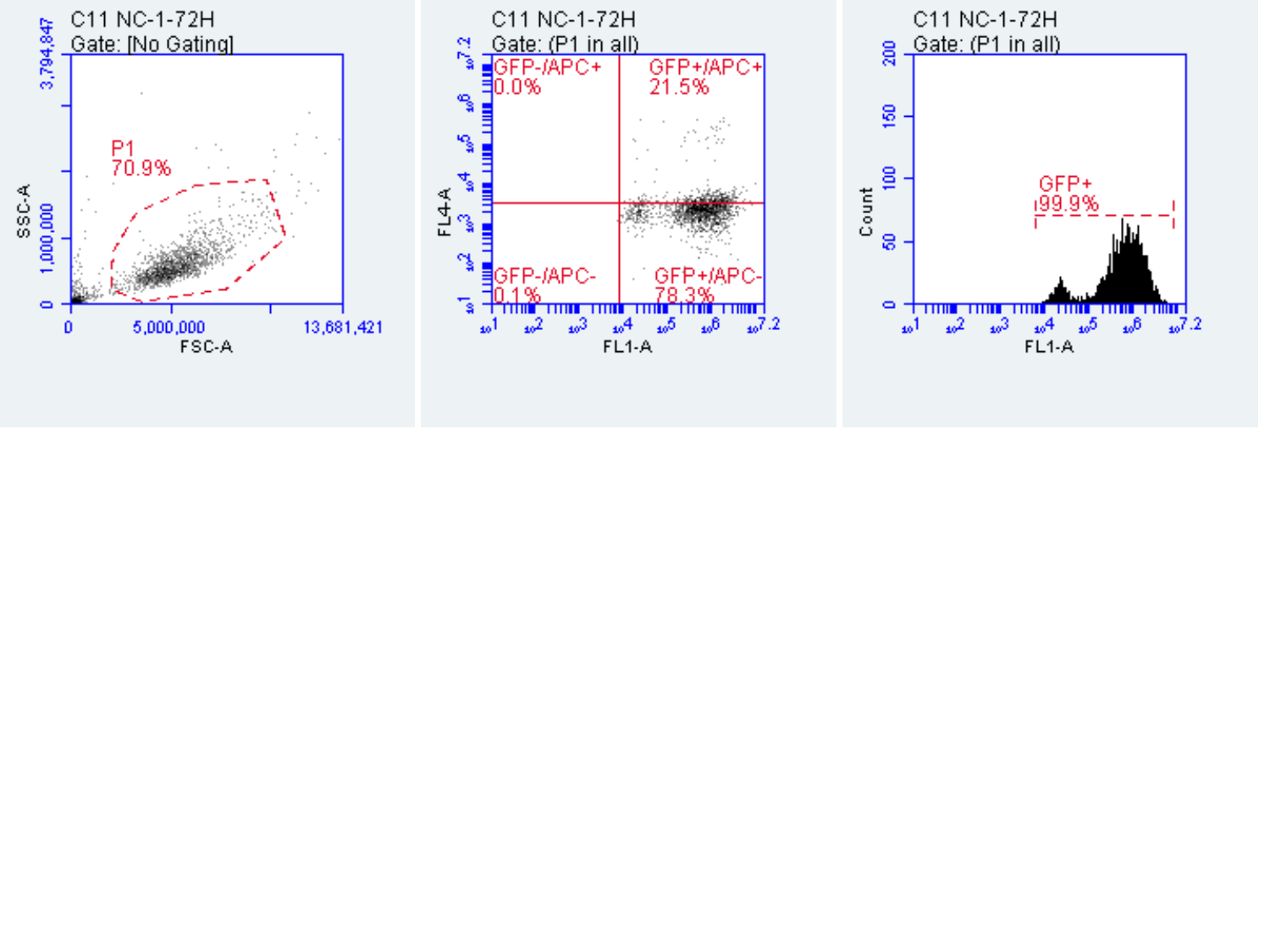

## Slide 10
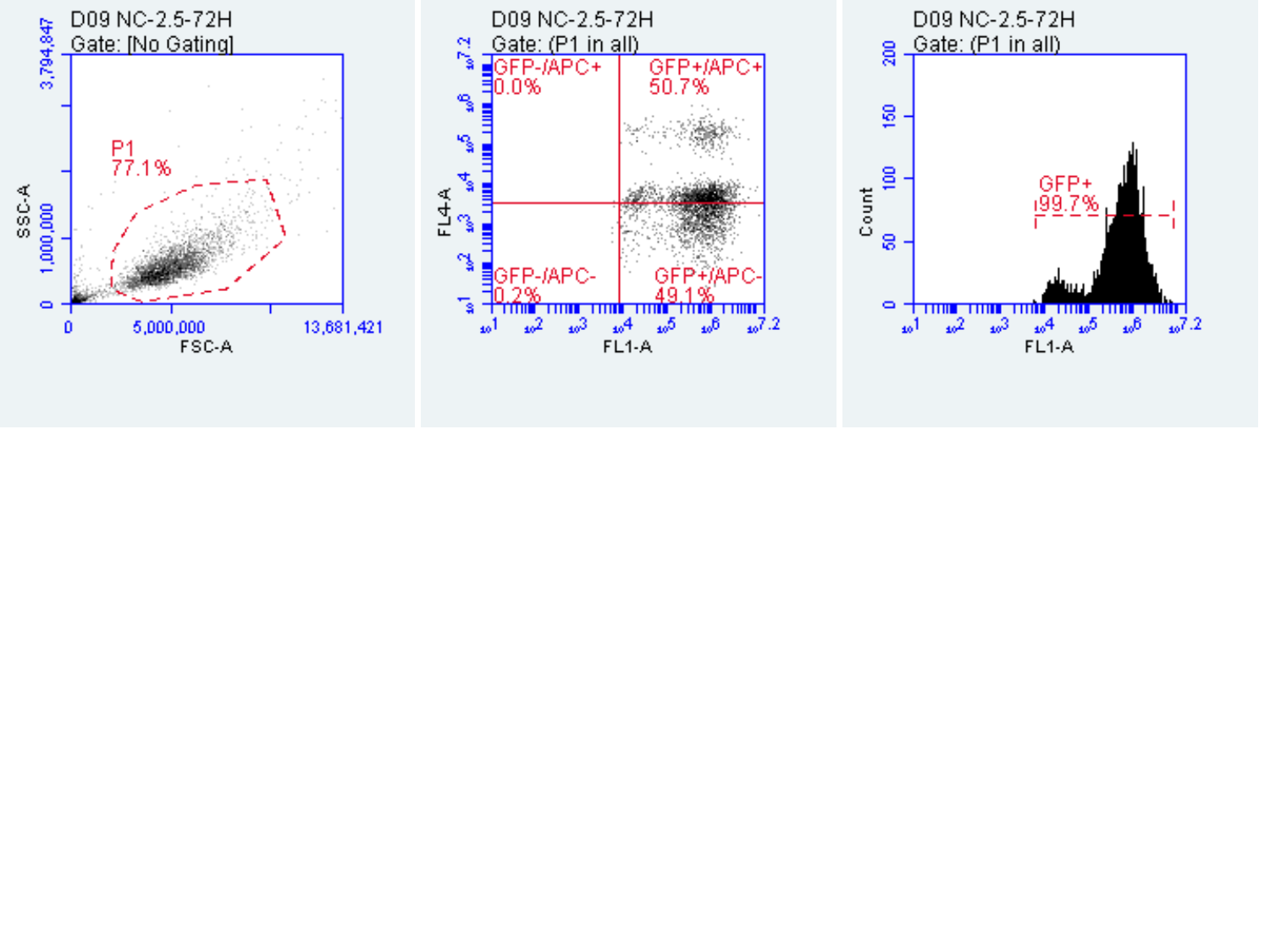

## Slide 11
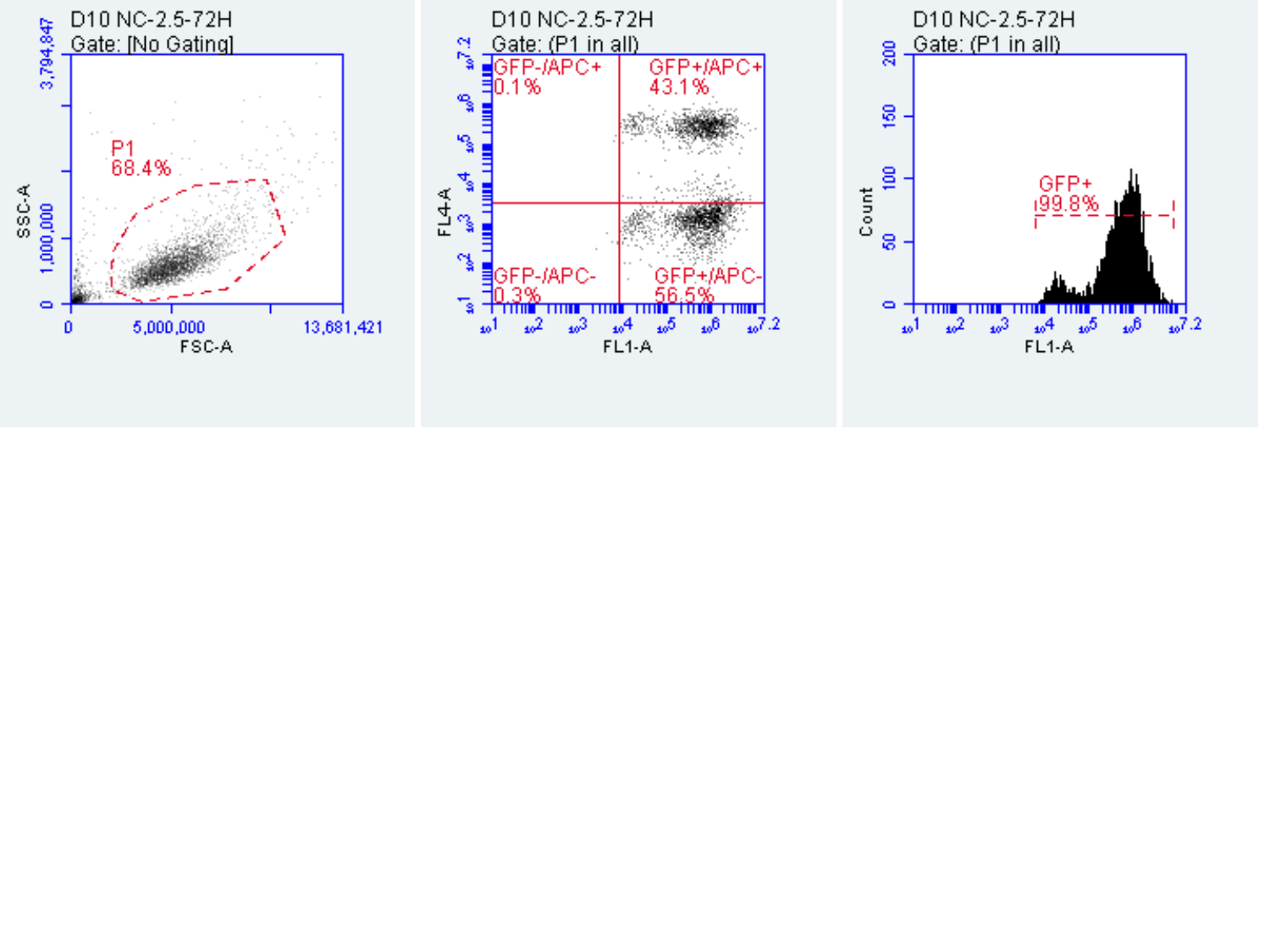

## Slide 12
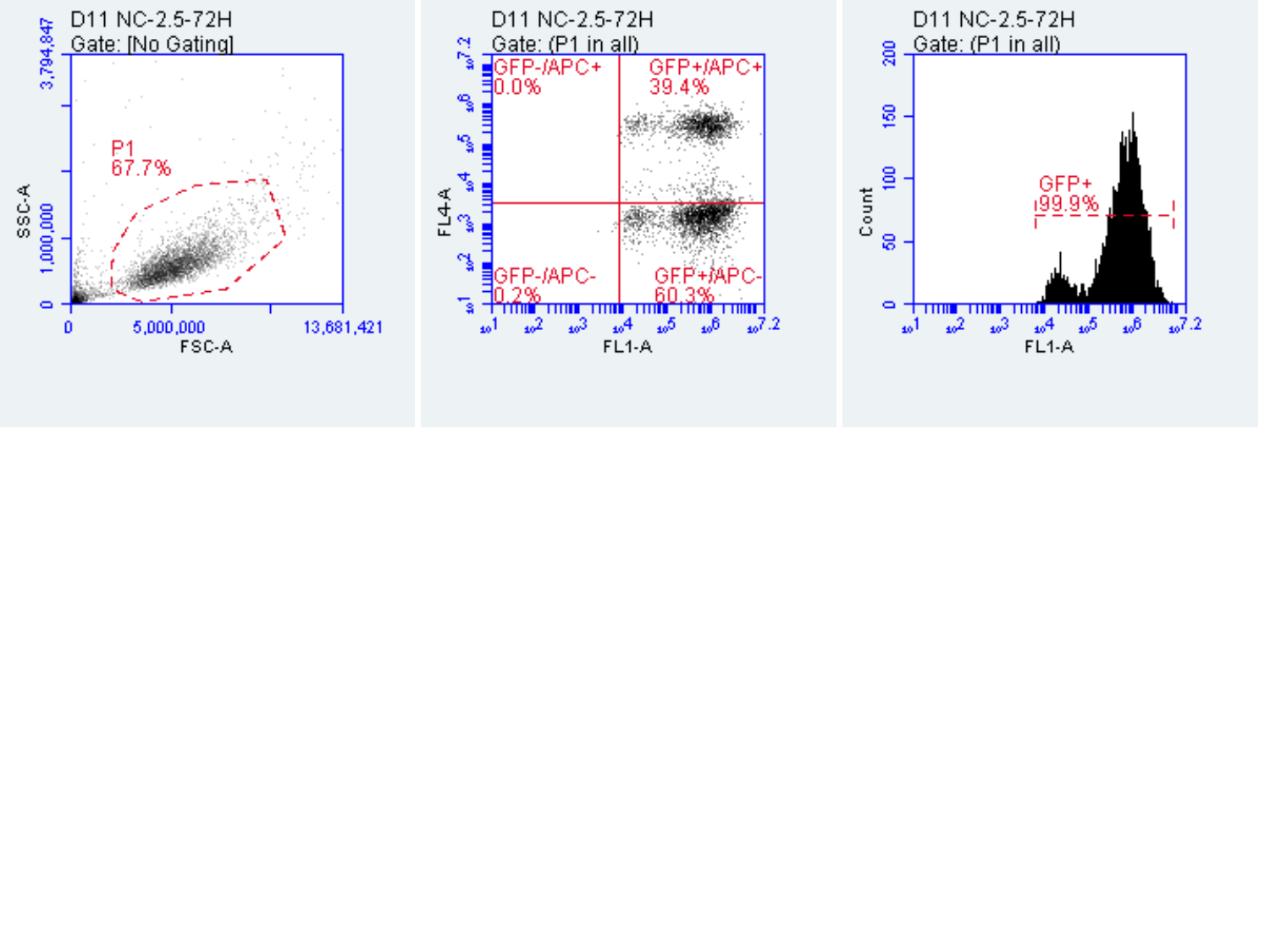

## Slide 13
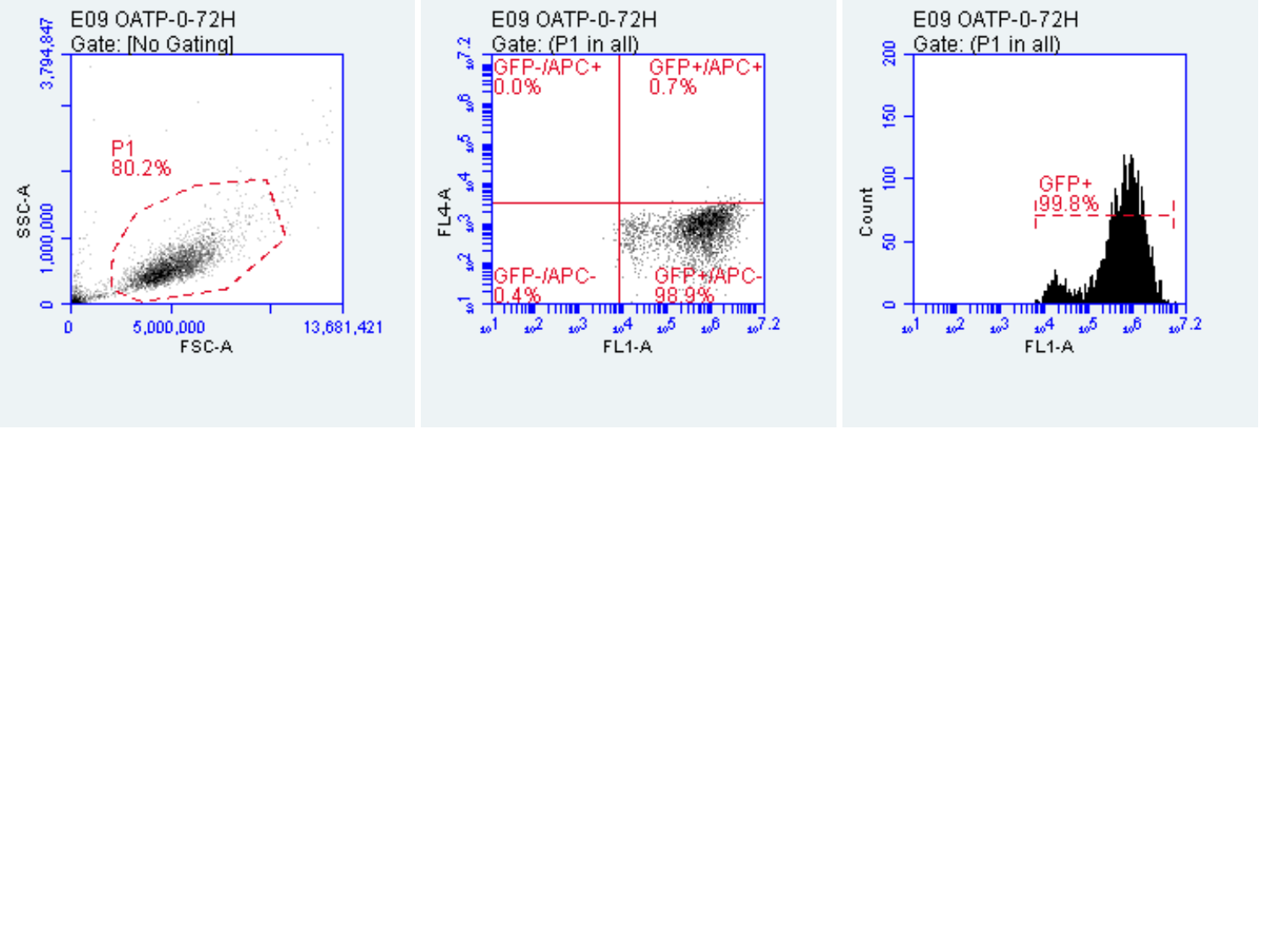

## Slide 14
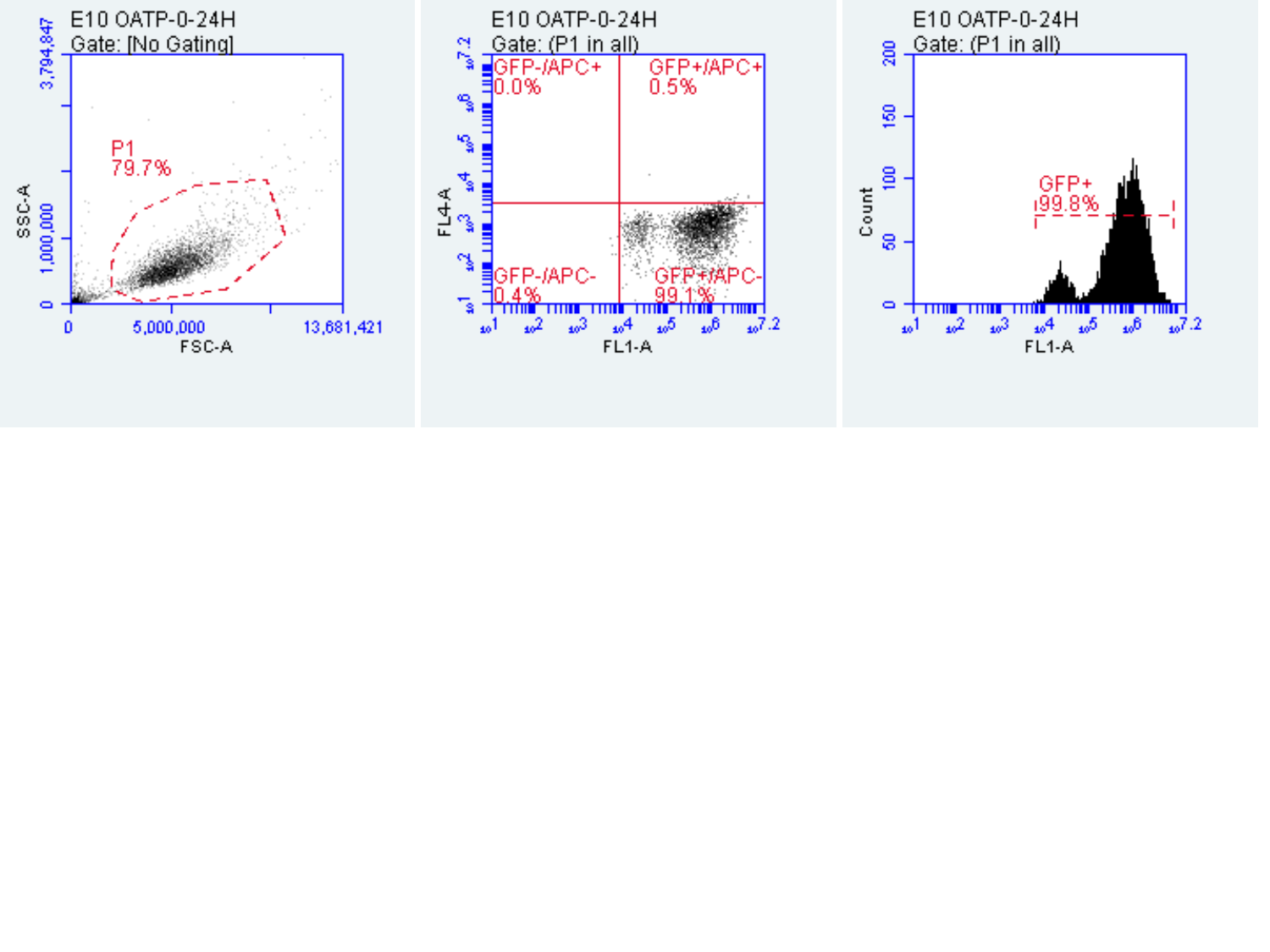

## Slide 15
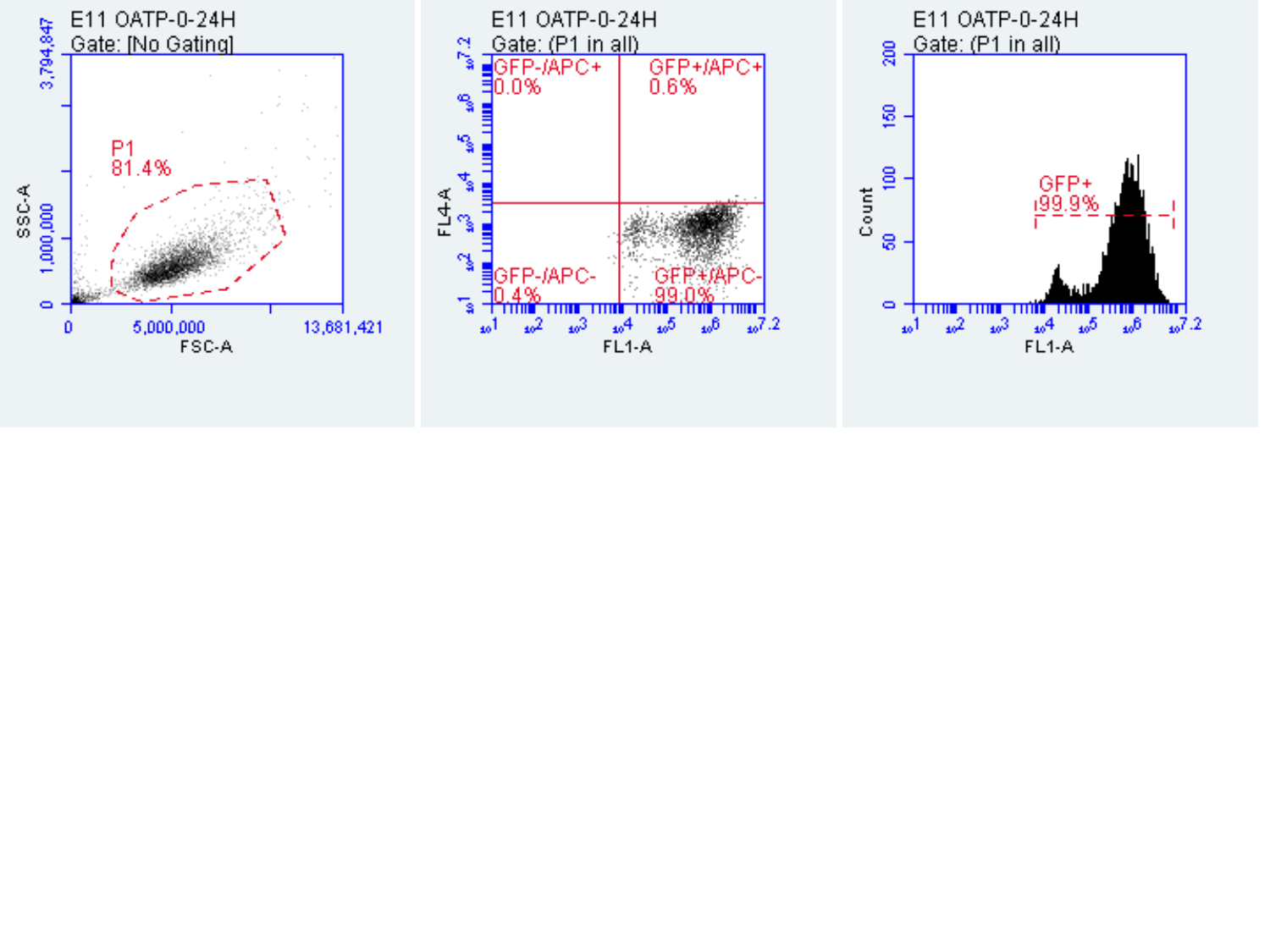

## Slide 16
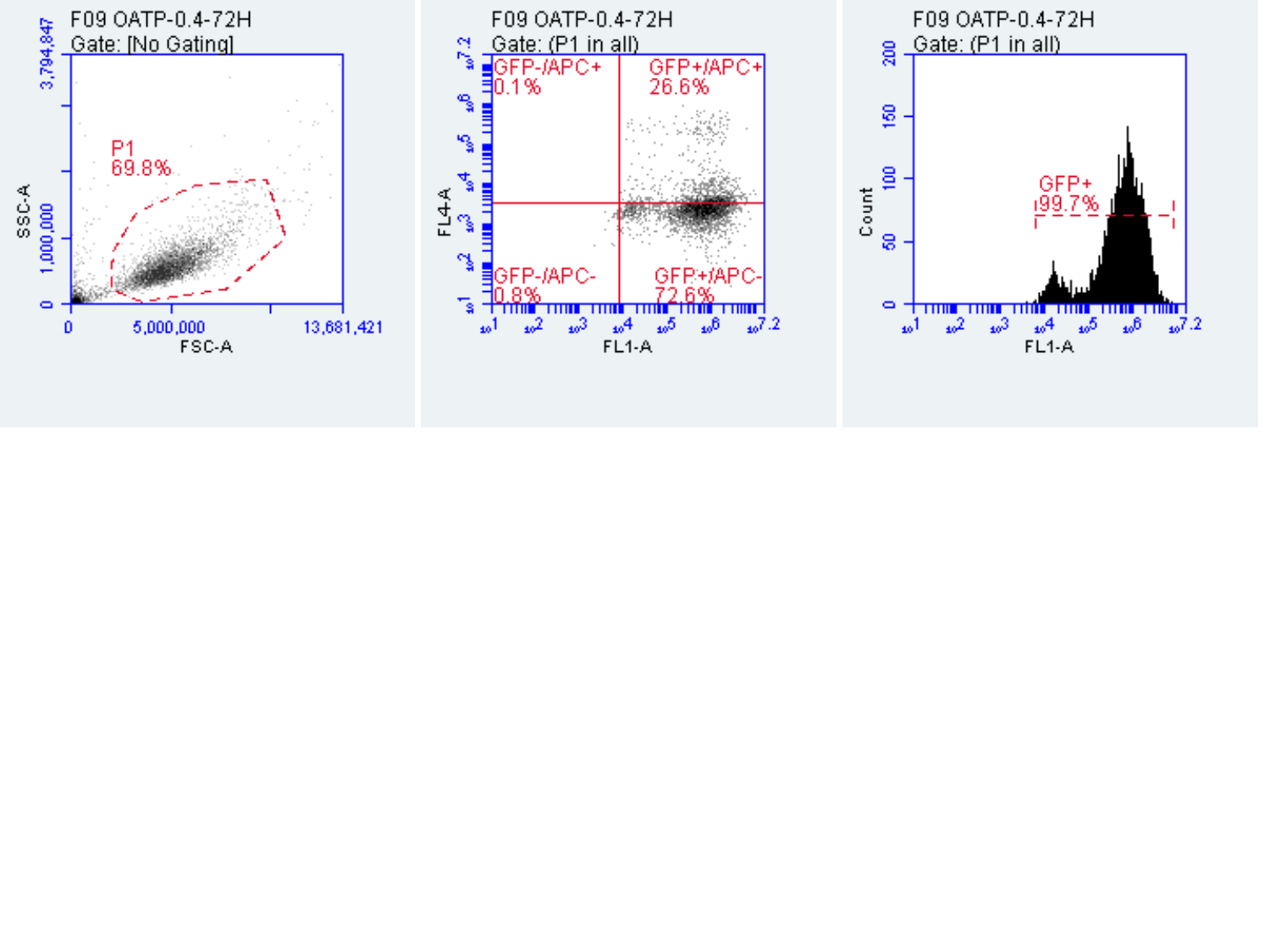

## Slide 17
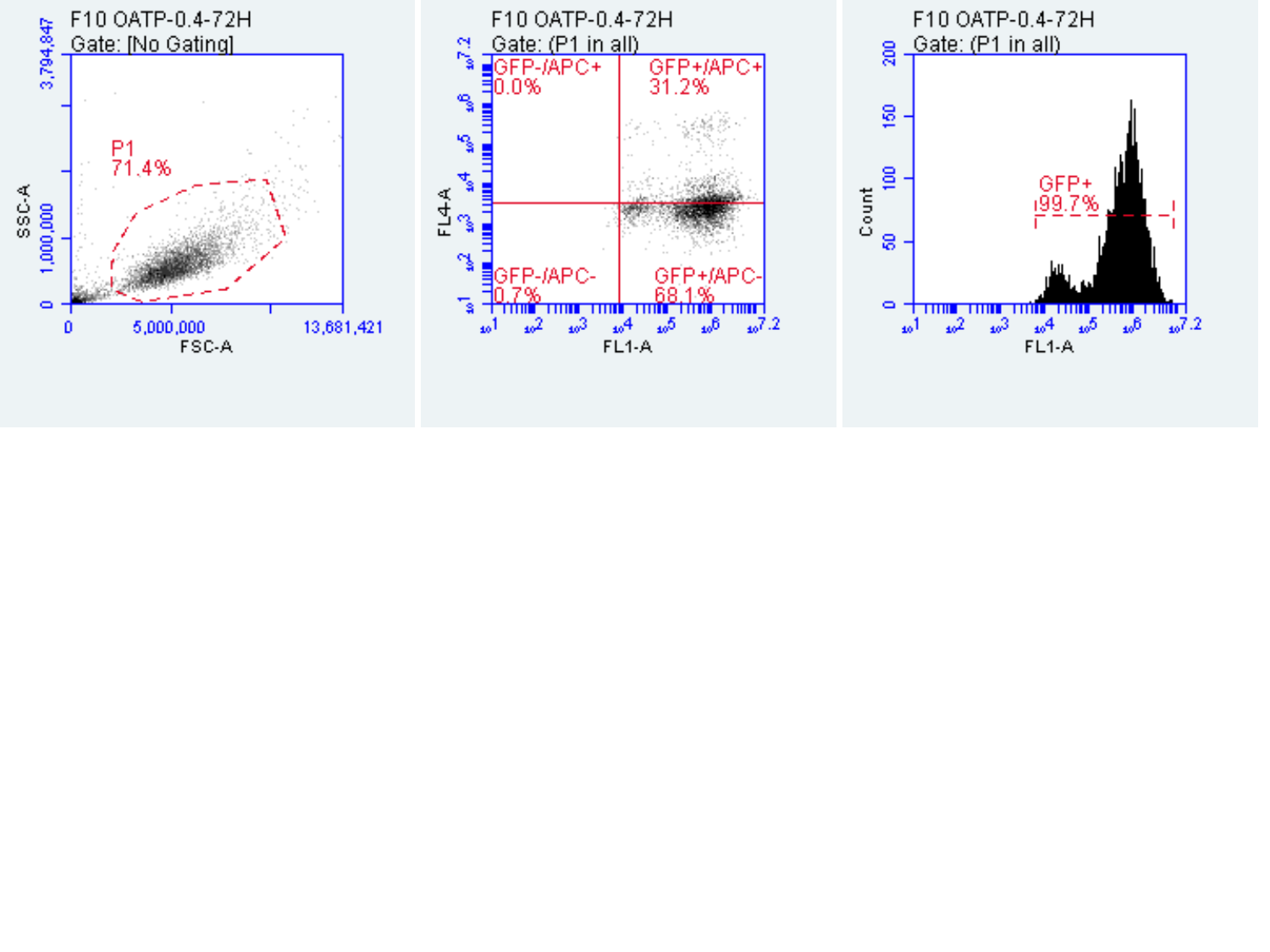

## Slide 18
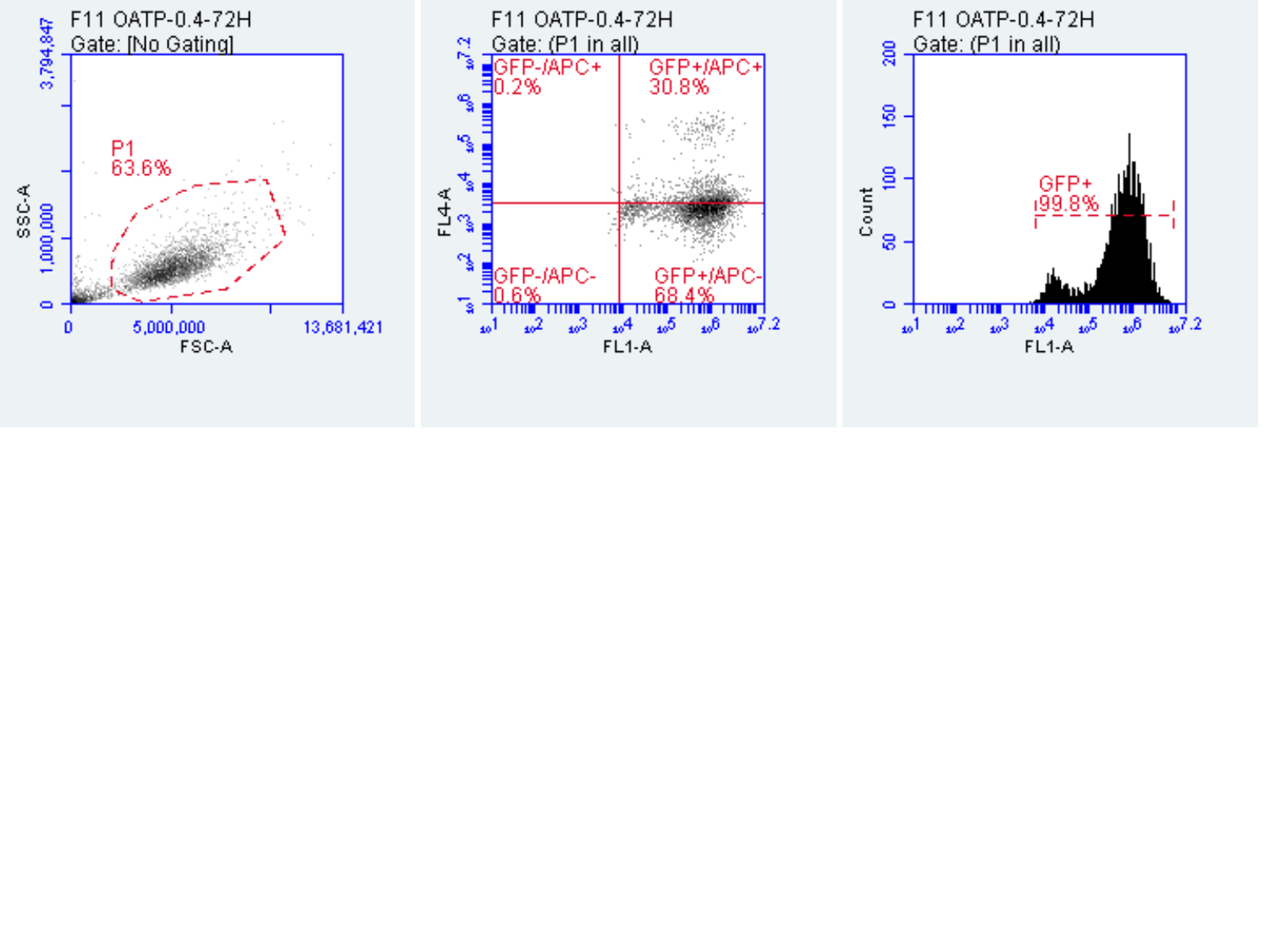

## Slide 19
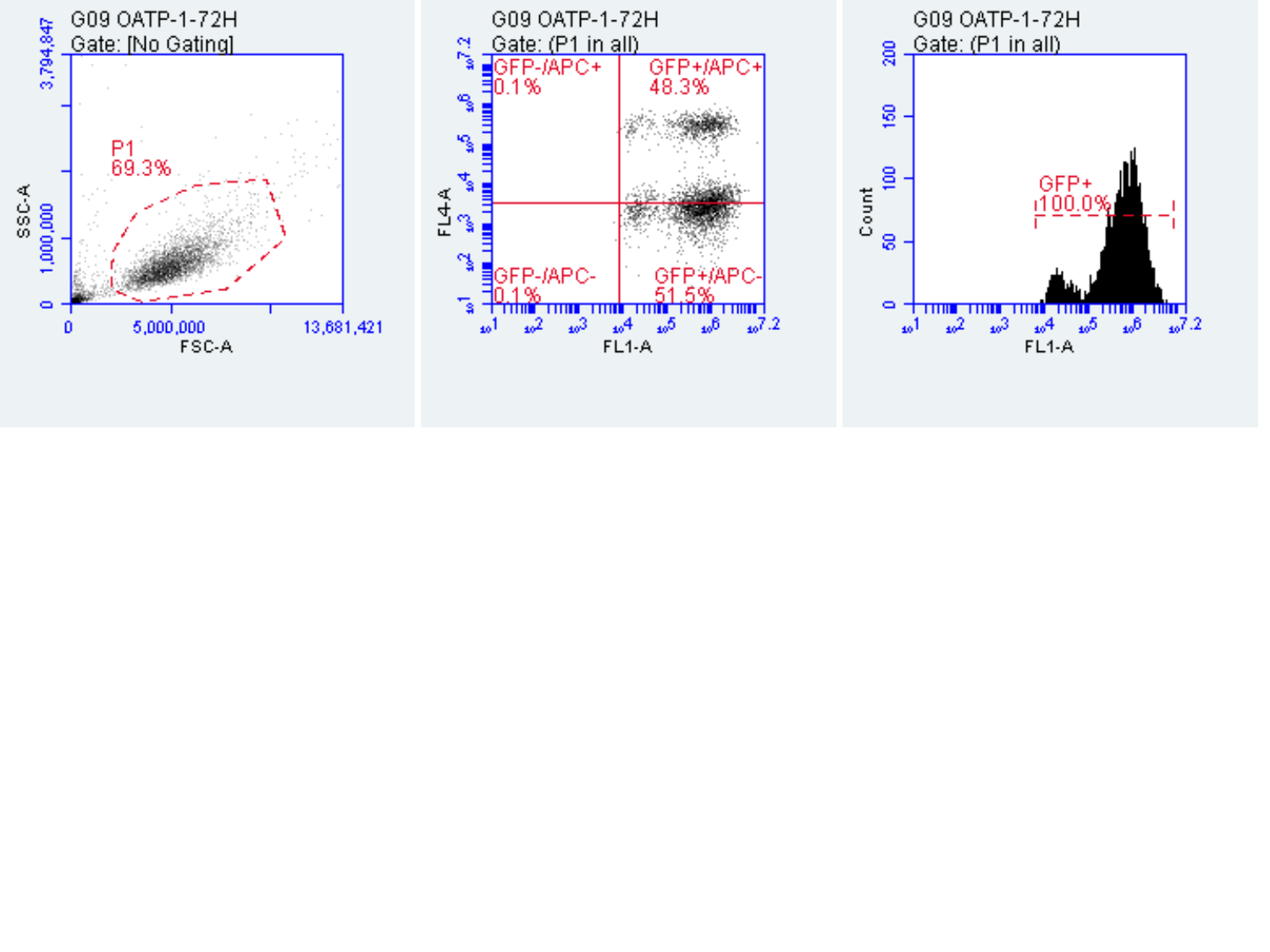

## Slide 20
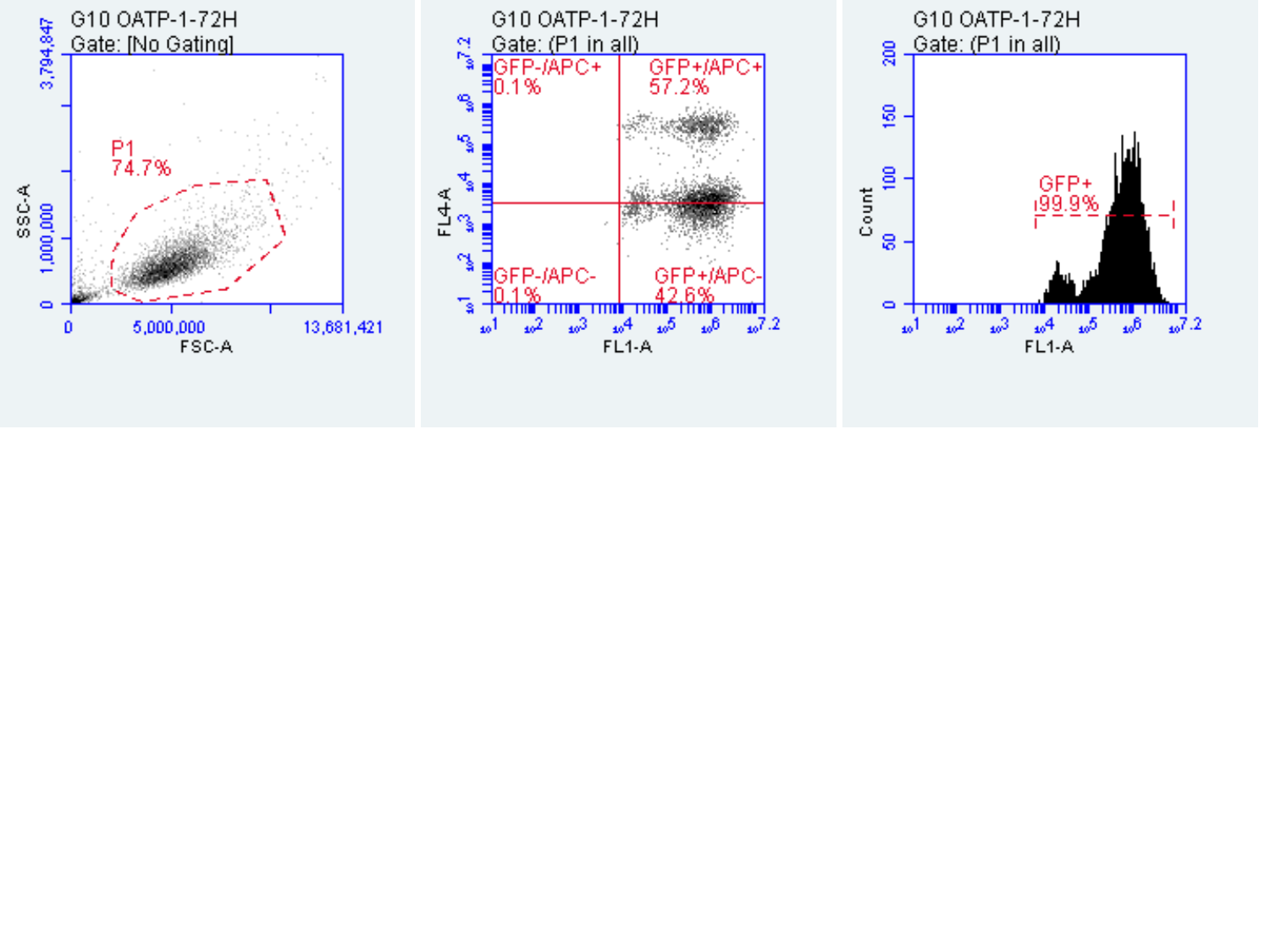

## Slide 21
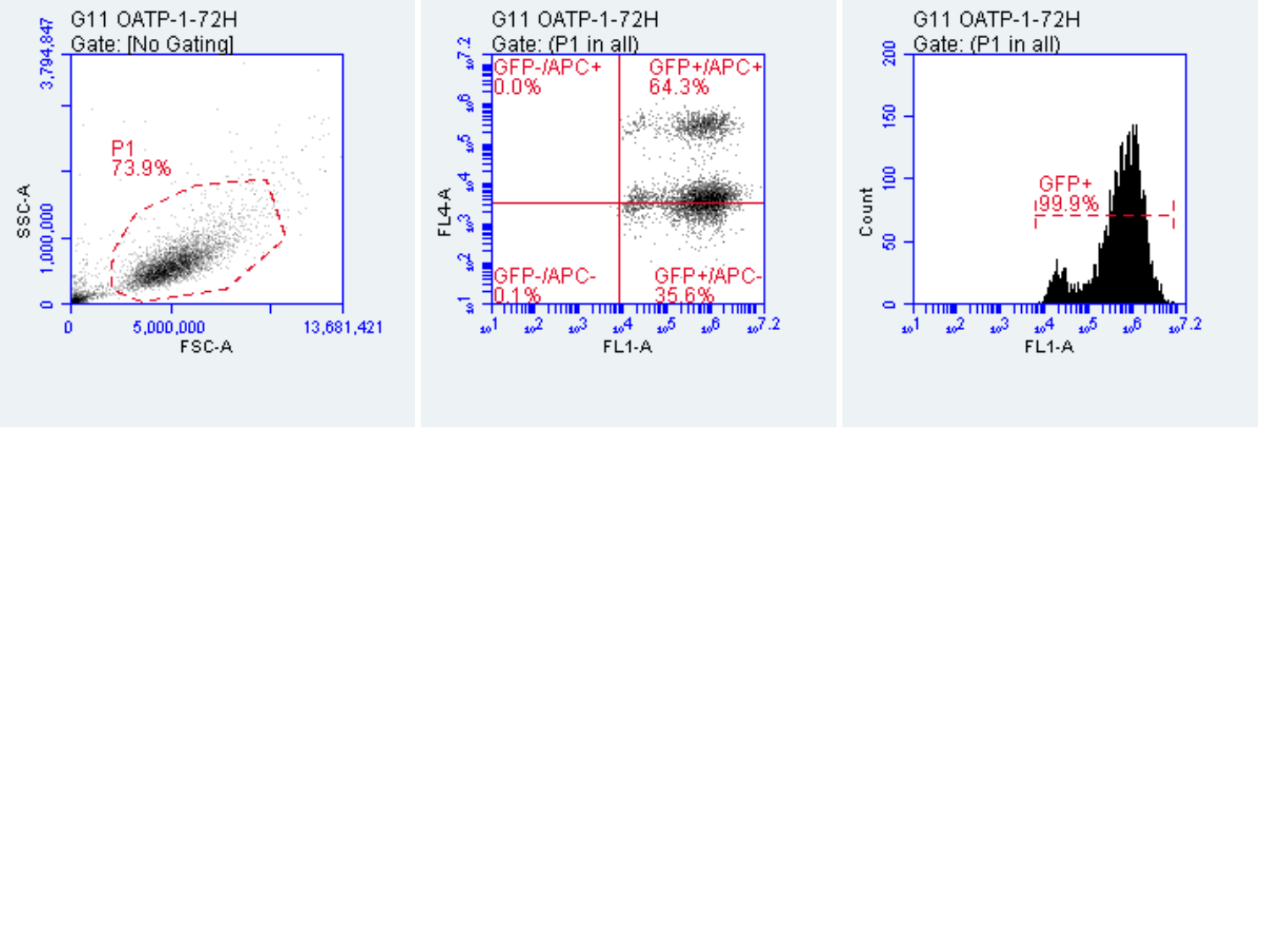

## Slide 22
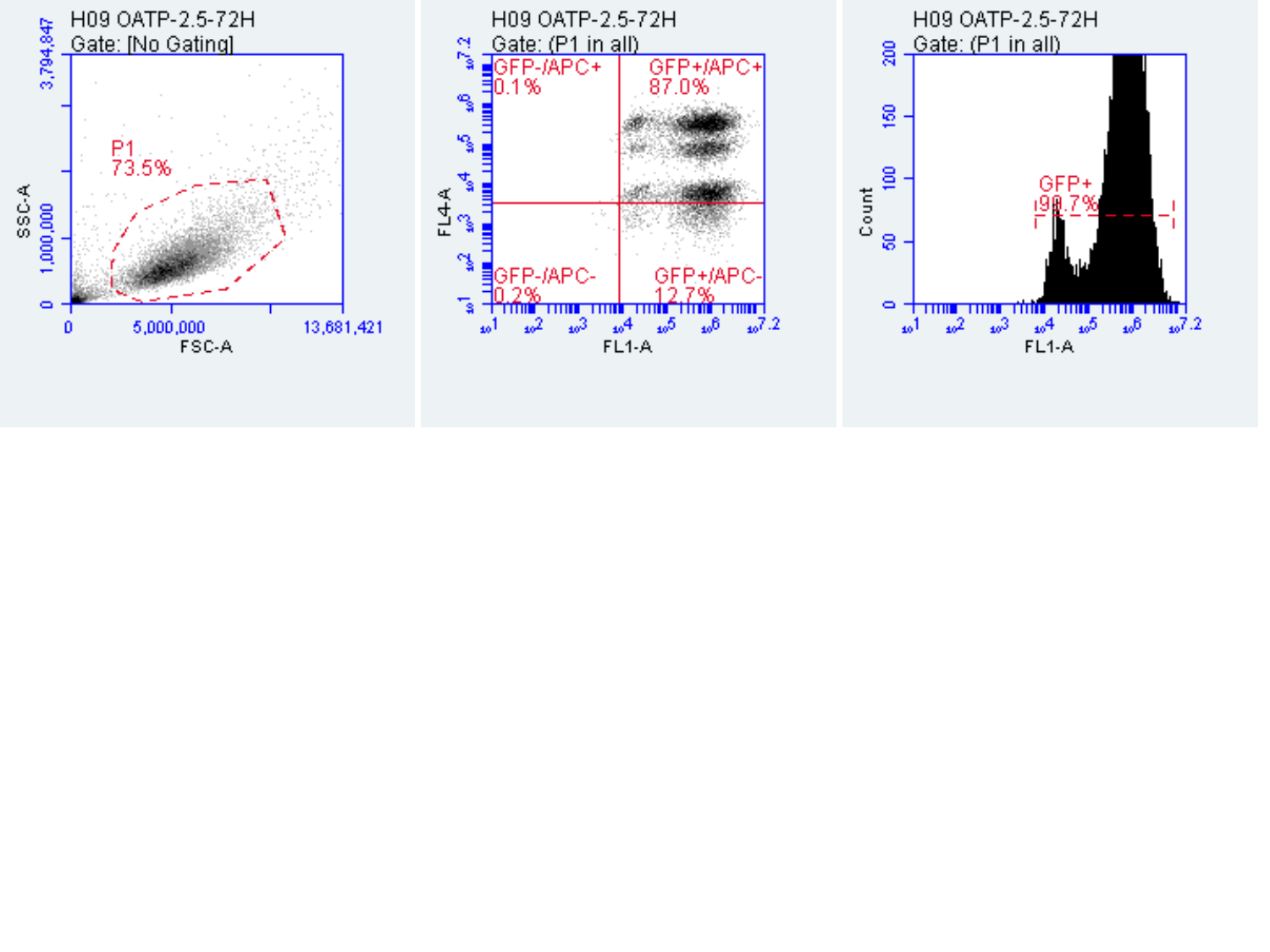

## Slide 23
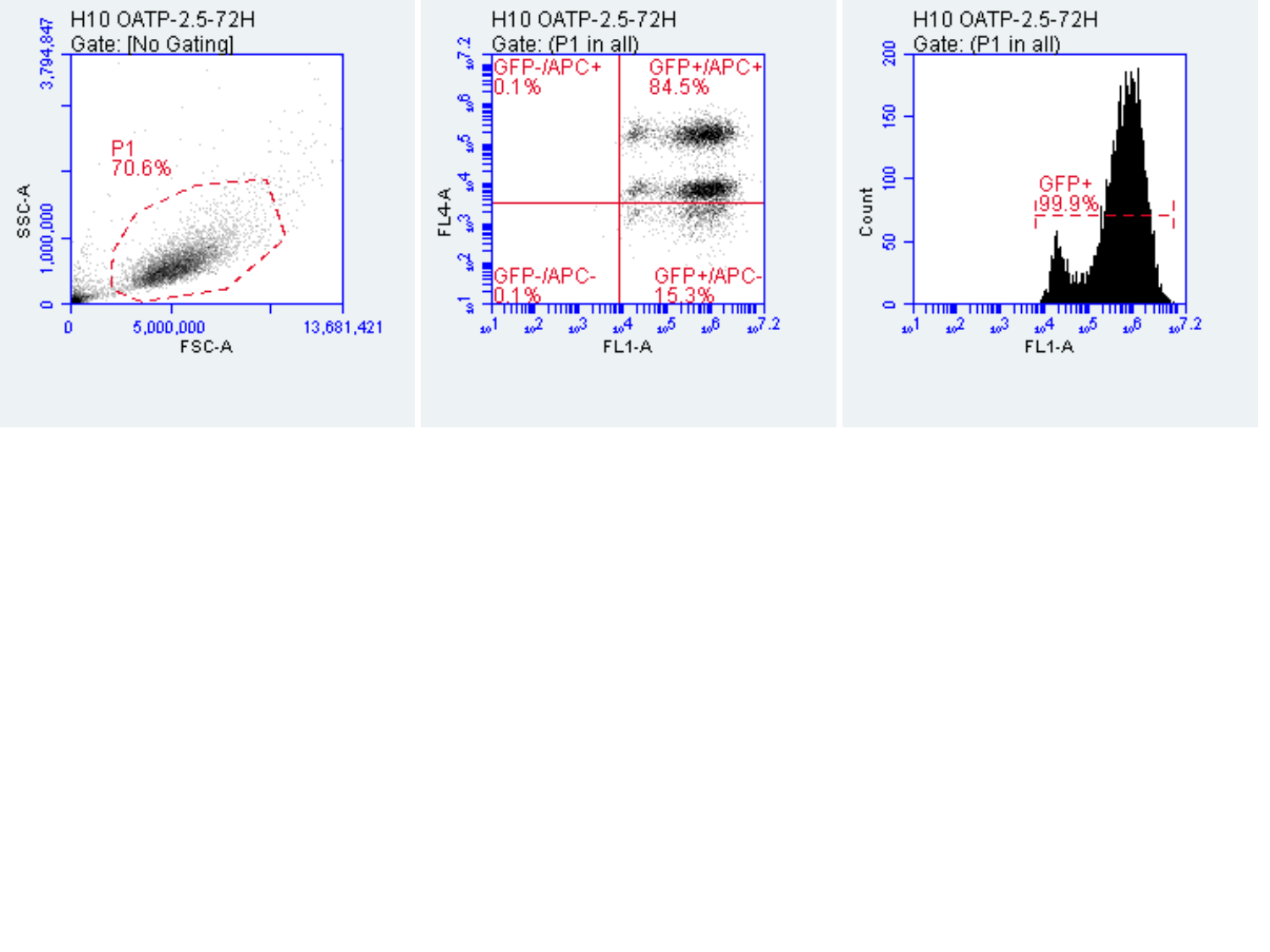

## Slide 24
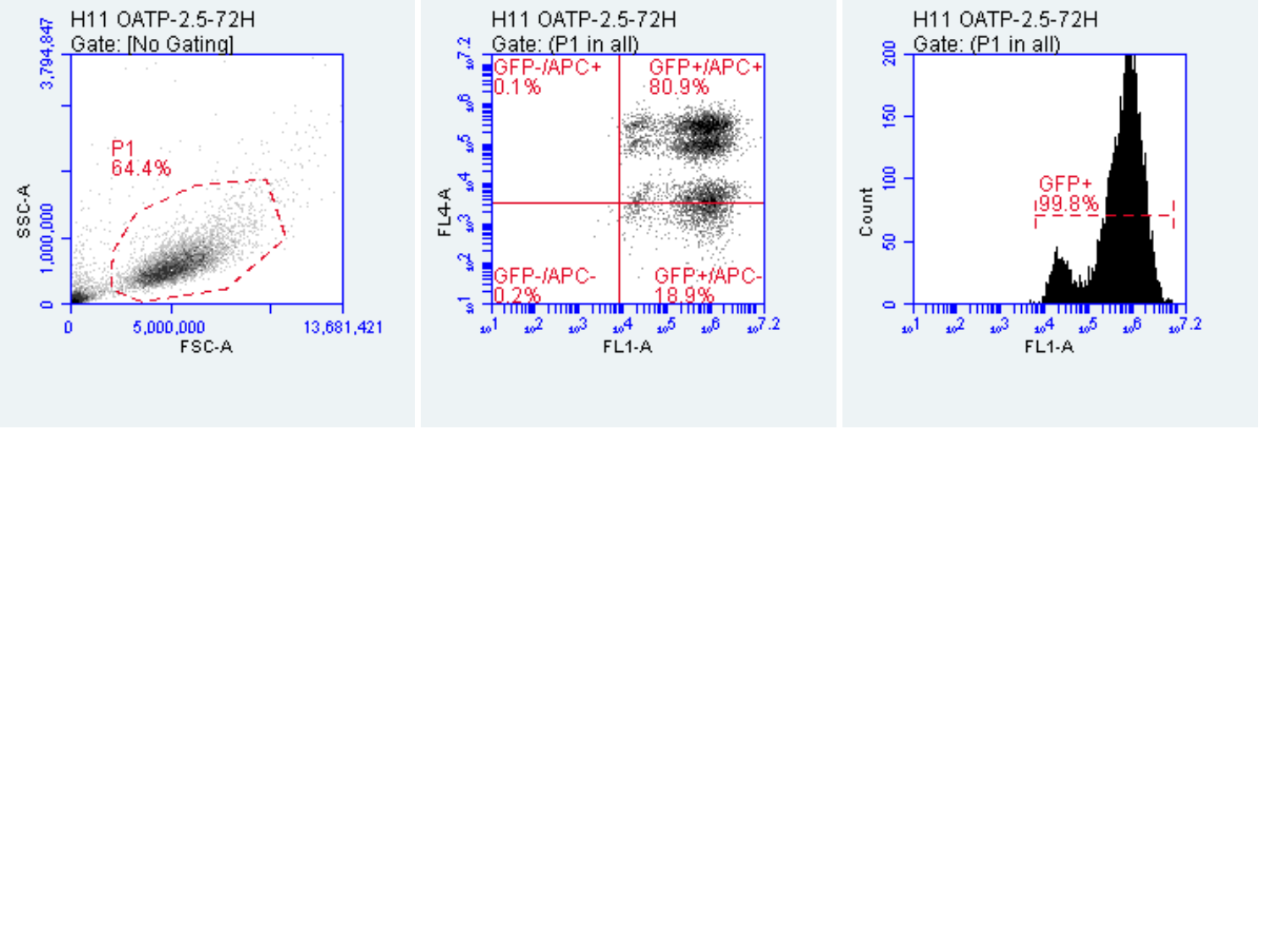

Supplement: Supplementary file 11 — Additional file 11: Raw Figures for flow cytometry after incubation for 72h in Figure 4. [file 12868_2021_658_MOESM11_ESM.ppt]
